# COVID-19 Hospitalization in Solid Organ Transplant Recipients on Immunosuppressive Therapy

**DOI:** 10.1001/jamanetworkopen.2023.42006

**Published:** 2023-11-07

**Authors:** Epiphane Kolla, Alain Weill, Mohamad Zaidan, Eleonora De Martin, Sylvie Colin De Verdiere, Laura Semenzato, Mahmoud Zureik, Lamiae Grimaldi

**Affiliations:** 1 EPI-PHARE, Scientific Interest Group in Epidemiology of Health Products, French National Agency for the Safety of Medicines and Health Products, French National Health Insurance, Saint-Denis, France; 2 Anti-Infective Evasion and Pharmacoepidemiology Team, INSERM UMR1018, School of Medicine Simone Veil, University Versailles Saint-Quentin-en-Yvelines, Paris-Saclay University, Montigny-Le-Bretonneux, France; 3 Department of Nephrology-Dialysis-Transplantation, Bicêtre University Hospital, Assistance Publique–Hôpitaux de Paris, Paris-Saclay University, Le Kremlin-Bicêtre, France; 4 Assistance Publique–Hôpitaux de Paris, Hepato-Biliary Centre, Paul Brousse Hospital, Unit INSERM 1193, Villejuif, France; 5 Departement of Lung Transplantation and Mucoviscidose Reference Centre, Foch Hospital, Suresnes, France; 6 Clinical Research Unit, Université Paris-Saclay, Direction of Clinical Research, Assistance Publique–Hôpitaux de Paris, Paris, France

## Abstract

**Importance:**

Solid organ transplant recipients are at high risk of severe infection with SARS-CoV-2 compared with the general population. However, factors associated with COVID-19–related severity in this population are still insufficiently explored in the literature.

**Objective:**

To examine which health conditions and immunosuppressive drugs for preventing graft rejection are associated with the risk of COVID-19–related hospitalization in solid organ transplant recipients.

**Design, Setting, and Participants:**

Using the French National Health Data System, this cohort study assessed patients of any age who received transplants between their date of birth and entry into the cohort on February 15, 2020. The cohort was followed up between February 15, 2020, and July 31, 2022.

**Exposures:**

Immunosuppressive drugs, including steroids, and health conditions (age, sex, and comorbidities).

**Main Outcomes and Measures:**

The main outcome was hospitalization for COVID-19, defined by main diagnostic *International Statistical Classification of Diseases and Related Health Problems, Tenth Revision* (*ICD-10*) codes. Factors associated with the outcome were identified with a nonconditional logistic regression. Confounding by indication was controlled using a multivariable model with adjustment for individual confounders. Each transplanted organ was examined separately.

**Results:**

Overall, 60 456 participants (median [IQR] age, 59 [47-67] years; 63.7% male) were included in the study, of whom 41 463 (68.6%) had kidney transplants, 14 464 (23.9%) had liver transplants, 5327 (8.8%) had heart transplants, and 2823 (4.6%) had lung transplants. Among them, 12.7% of kidney transplant recipients, 6.4% of liver transplant recipients, 12.9% of heart transplant recipients, and 18.0% of lung transplant recipients were hospitalized for COVID-19. In kidney transplant recipients, steroids (adjusted odds ratio [AOR], 1.60; 95% CI, 1.49-1.73) and mycophenolic acid (AOR, 1.37; 95% CI, 1.25-1.51) were associated with a high risk of hospitalization. In liver transplant recipients, tacrolimus (AOR, 0.77; 95% CI, 0.61-0.98) was associated with a decreased risk, and steroids (AOR, 1.60; 95% CI, 1.38-1.86) and mycophenolic acid (AOR, 1.61; 95% CI, 1.37-1.90) were associated with an increased risk of hospitalizations. In heart transplant recipients, cyclosporine (AOR, 0.67; 95% CI, 0.47-0.94) was associated with a decreased risk, and steroids (AOR, 1.42; 95% CI, 1.11-1.82), mycophenolic acid (AOR, 1.29; 95% CI, 1.02-1.64), sirolimus (AOR, 2.71; 95% CI, 1.20-6.09), and everolimus (AOR, 1.24; 95% CI, 1.01-1.51) were associated with an increased risk of hospitalization. Only steroids (AOR, 1.72; 95% CI, 1.19-2.48) were associated with a high risk of COVID-19 hospitalization in lung transplant recipients.

**Conclusions and Relevance:**

This study suggests that mycophenolic acid, sirolimus, and steroids are associated with an increased risk of COVID-19–related hospitalization in solid organ transplant recipients. These results should be considered by clinicians treating transplant recipients and may help inform epidemic-related decisions for this population in the future.

## Key Points

**Question:**

Does maintenance therapy with immunosuppressive drugs to prevent graft rejection increase the risk of COVID-19–related hospitalization in solid organ transplant recipients?

**Findings:**

In this cohort study of 60 456 solid organ transplant recipients, mycophenolic acid and steroids were associated with a higher risk of hospitalization (relative risk increase of 29%-72%), and tacrolimus and cyclosporine in liver and heart transplant patients were associated with a decreased risk of hospitalization (relative risk decrease of 23% for liver transplant patients and 33% for heart transplant patients) compared with the other immunosuppressive drugs.

**Meaning:**

These findings suggest that maintenance immunosuppressive drugs are associated with an increased risk of COVID-19 hospitalization in solid organ transplant recipients, who need to be closely monitored.

## Teaser

This cohort study assesses whether patient health conditions and immunosuppressive drug use for the prevention of graft rejection are associated with risk of COVID-19–related hospitalization in solid organ transplant recipients.

## Introduction

COVID-19, which is caused by SARS-CoV-2 infection, has affected every nation worldwide since the first cases appeared in November 2019 in Wuhan, China. According to the World Health Organization, more than 662 million confirmed cases of infection and 6 million deaths have been reported as of January 2023.^1^ SARS-CoV-2 infection is often asymptomatic but can sometimes progress to severe disease and death. Risk factors for progression to the severe form of COVID-19 include age older than 60 years, male sex, and comorbidities (eg, high blood pressure, diabetes, obesity, and immunosuppression).^2,3,4^

Solid organ transplant recipients (SOTRs) receive lifelong immunosuppressive drugs, drastically limiting the risk of transplant organ rejection.^5^ Because of their immunosuppression combined with comorbidities, SOTRs are considered to be at high risk for severe forms of COVID-19. Several studies have reported an increased risk of severe disease or death related to COVID-19 in SOTRs compared with the general population.^6,7,8,9,10^ In their studies of the French population, Semenzato et al^2,3^ report an increased risk of developing severe SARS-CoV-2 infection not only in SOTRs unvaccinated against SARS-CoV-2 in 2020 but also in those with complete vaccination in 2021.

However, most of these studies have several limitations, including a lack of power due to the small sample size and the failure to consider the different immunosuppressive therapies in the analyses. Indeed, immunosuppressive drugs to protect against graft rejection could be more or less associated with the risk of developing severe forms of COVID-19. In addition, factors of COVID-19–related severity in this population, such as the time since transplantation or immunosuppression, are insufficiently explored in the literature. Our study aims to identify factors, including immunosuppressive therapies such as tacrolimus, cyclosporine, azathioprine, mycophenolic acid, sirolimus, everolimus, and steroids, that are associated with COVID-19–related hospitalization in SOTRs treated in France.

## Methods

This cohort study was authorized by decree 2016-1871 from December 26, 2016, relating to the processing of personal data from the National Health Data System and French law articles 1461-13 and 14. The EPI-PHARE (Épidémiologie des Produits de Santé) team, which has permanent regulatory access to the data from the French National Health Data System (Système National des Données de Santé [SNDS]), was exempted from participant consent and institutional review board approval. The study was registered on the study register of EPI-PHARE concerning studies from SNDS data under reference T-2022-06-406. The French National Health Insurance Fund (Caisse nationale de l’Assurance Maladie/CNAM) recorded the data. This study followed the Strengthening the Reporting of Observational Studies in Epidemiology (STROBE) reporting guideline.

### Data Sources

We conducted a cohort study using the SNDS. The SNDS records all inpatient and outpatient claims and hospital discharges for 99.5% of the 67 million inhabitants of France. Each patient is given a unique anonymous identifier (encrypted twice in the SNDS) that is valid from birth (or immigration) to death (or emigration definitive). This identifier is linked to information from the DCIR (Datamart de Consommation Inter-Regimes, the national health insurance reimbursement database), and the PMSI (Programme de Médicalisation des Systèmes d’Information, the national hospital discharge database).

The DCIR database contains individual demographic information on beneficiaries, ambulatory medical care data, reimbursed drug delivery, and health care expenses for patients with long-term conditions.^11,12,13^ The PMSI database indicates all public and private hospital admissions in France and hospital and discharge diagnoses coded according to the *International Statistical Classification of Diseases and Related Health Problems, Tenth Revision* (*ICD-10*). Medical and surgical procedures are coded according to the French Classification of Medical Procedures. These databases were fully described elsewhere and are regularly used for drug monitoring.^14,15,16^

### Study Population

We identified SOTRs affiliated with a French health insurance scheme between the date of birth and entry into the cohort on February 15, 2020. We included SOTRs who received at least 1 reimbursement for an immunosuppressive drug (ie, tacrolimus, cyclosporine, azathioprine, mycophenolic acid, sirolimus, and everolimus) in the 6 months before the date of entry. Transplants were defined by *ICD-10* codes, the French Common Classification of Medical Procedures, and diagnosis-related groups (eTable 1 in Supplement 1). Kidney transplants in patients undergoing dialysis were excluded. Immunosuppressive drugs were defined by the Anatomical Therapeutic Chemical Classification System. Some patients in the study population received more than 1 transplant. The cohort was followed up from February 15, 2020, to July 31, 2022.

### Covariates

We considered the patient’s age, sex, and geographic area of residence. Age was defined from the patient’s year of birth as a categorical variable in 3 age groups, and the residence area was determined by telephone codes in France, delimiting 5 geographic regions. We used the Social Deprivation Index as a measure of socioeconomic status. This indicator ranges from quintiles 1 to 5 (with 1 indicating least deprived and 5 indicating most deprived) and is based on the median household income, the percentage of high school graduates in the population older than 15 years, the percentage of manual workers in the labor force, and the unemployment rate for the person’s town of residence.^17^ Comorbidities strongly related to COVID-19 were defined using mapping algorithms developed from the DCIR and PMSI databases, allowing for the identification of diseases.^18,19^ The mapping algorithms identified patients with several diseases in 2019 and were complemented by identifying patients with obesity, smokers, and people with alcohol use disorders (eTable 2 in Supplement 1). The difference between the date of transplant and the date of entry into the cohort determined the time since transplant, a categorical variable that included grouping together recent transplant recipients (who benefit from high immunosuppression in the first or second year after transplant). Steroid use was defined as reimbursement of prednisone, prednisolone, or methylprednisolone 6 months before entry into the cohort. We also assessed the dose of steroids (in prednisolone dose) and mycophenolic acid by calculating the quartiles corresponding to the cumulative daily dose. Markers of health status, such as the number of consultations and hospitalizations 6 months before entry into the cohort, were also taken into account.

### Primary Outcome

Our study was based on hospital discharge data available on July 31, 2022. We restricted the analyses to July 2022, the most extended follow-up we could obtain. The primary end point was hospitalization for COVID-19. The following *ICD-10* codes reflect a hospitalization for COVID-19 for the main or related diagnosis: U07.10 (COVID-19, respiratory form, virus identified), U07.11 (COVID-19, respiratory form, virus unidentified), U07.14 (COVID-19, other clinical forms, virus identified), and U07.15 (COVID-19, other clinical forms, virus unidentified).^13,20^ We excluded hospitalized patients with COVID-19 as an associated diagnosis (hospitalization for another indication during which the patient was also found to have COVID-19).

### Statistical Analysis

Differences in sociodemographic characteristics, comorbidities, immunosuppressive therapy, and steroid use proportions were described by transplant type and hospitalization groups. The χ^2^ test was used to analyze categorical variables and the *t* test to analyze quantitative variables with a normal distribution (Wilcoxon test for those deviating from normality). Nonconditional logistic regression was used to identify immunosuppressive therapies, including steroids, and other factors associated with the outcome in the main analysis. We calculated crude and adjusted odds ratios (AORs) and their 95% CIs. The adjustment factors for the multivariable analysis were all variables significantly associated with hospitalization for COVID-19 and those described in the literature as worsening factors for COVID-19 (age, sex, region, Social Deprivation Index, previous consultations and hospitalizations, comorbidities, time since transplant, and immunosuppressive drugs). Immunosuppressive drugs, including steroids, were considered in the main model as 6 binary variables by adjusting for each other. Sensitivity analyses were performed, excluding patients with more than 1 transplant, according to the time after transplant, the period before and after the start of vaccination, and the periods before, during, and after the highest epidemic wave (November 2021 to February 2022). We also performed exploratory analyses by considering medications as a categorical variable (combinations of immunosuppressive drugs, combinations of immunosuppressive drug classes, and treatment intensity). Treatment intensity was defined as the number of drugs the patient received during the 6 months before entry into the cohort. All statistical tests were 2-tailed, with a type I error of 5%. Statistical analyses were performed using SAS software, version 7.1 (SAS Institute Inc).

## Results

Our study included 60 456 SOTRs (median [IQR] age, 59 [47-67] years; 63.7% male and 36.3% female) on February 15, 2020, who were taking an immunosuppressive drug for the prevention of graft rejection that was reimbursed within the previous 6 months (Figure). Within this study population, 41 463 (68.6%) had kidney transplants, 14 464 (23.9%) had liver transplants, 5327 (8.8%) had heart transplants, and 2823 (4.6%) had lung transplants (eTable 3 in Supplement 1). The most common comorbidities found in our cohort were hypertension and diabetes. A history of alcohol consumption and smoking was reported for 53.3% of liver transplants recipients and 43.0% of lung transplant recipients. Tacrolimus was used at least once in 70.1% and mycophenolic acid in 72.7% of SOTRs, cyclosporine in 21.0%, and azathioprine in 6.9%. Sirolimus was used in 2.3% and everolimus in 11.6% of the study population. Everolimus was received by 28.8% of heart transplant recipients. More than 60% received steroids in each organ transplant subgroup except liver transplant recipients, of whom only 28.8% received steroids.

**Figure.**
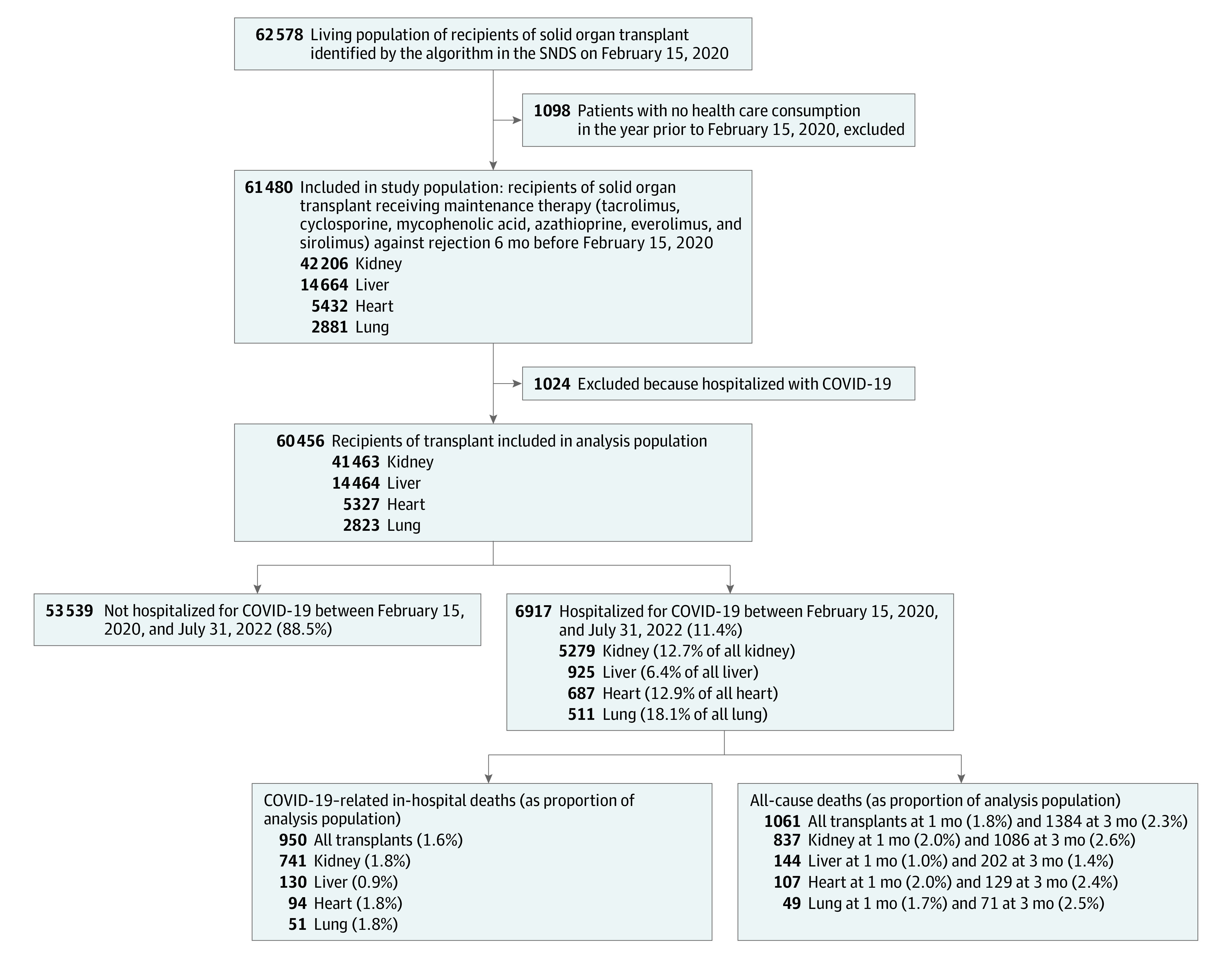
Study Flowchart Some patients received more than 1 transplant. SNDS indicates French National Health Data System (Système National des Données de Santé).

During the study period, 6917 SOTRs (11.4%) were hospitalized for COVID-19, and 950 died. The median (IQR) length of stay for those hospitalized was 5 (1-13) days. Hospitalization rates were 12.7% for kidney transplant recipients, 6.4% for liver transplant recipients, 12.9% for heart transplant recipients, and 18.0% for lung transplant recipients. We identified 7 epidemic peaks in France during our study period (eFigure 1 in Supplement 1). Baseline characteristics, comorbidities, and immunosuppressive drugs according to the groups of hospitalizations are presented in Table 1 and Table 2. Among kidney transplant patients, the median (IQR) age of those hospitalized was 60 (50-69) years, and 60.6% were male. Hospitalized kidney transplant patients were most likely to live in areas with greater deprivation (23.0% for Social Deprivation Index quintile 5) and had more comorbidities compared with nonhospitalized patients, including obesity (25.6% vs 17.9%), hypertension (85.9% vs 81.0%), cardiovascular issues (36.1% vs 27.1%), diabetes (39.6% vs 26.1%), and respiratory issues (11.3% vs 7.8%). They were more likely to be recent transplant recipients (<2 years: 17.6% vs 14.1%). Frequency of immunosuppressive drug use was similar between hospitalized and nonhospitalized kidney transplant patients, except for mycophenolic acid (80.6% vs 74.8%) and steroids (79.3% vs 67.4%). Hospitalized liver transplant recipients had a median (IQR) age of 64 (54-70) years and had the same comorbidities as kidney transplant recipients compared with nonhospitalized patients, in addition to dialysis (2.3% vs 0.6%), tacrolimus use (73.1% vs 76.9%), mycophenolic acid use (72.3% vs 61.5%), and steroids use (40.2% vs 28.0%). Heart and lung transplant recipients’ characteristics are also presented in Table 1 and Table 2. Descriptive analysis according to the immunosuppressive drugs is presented in eTables 4, 6, 9, and 11 in Supplement 1. Immunosuppressive drug use according to the time since transplant and treatment regimens are described in eFigures 2-6 in Supplement 1.

**Table 1.** Baseline Characteristics of Solid Organ Transplant Recipients in France According to COVID-19 Hospitalization Groups^a^

Characteristic	Whole transplant cohort (N = 60 456)		Kidney transplant (n = 41 463)		Liver transplant (n = 14 464)		Heart transplant (n = 5327)		Lung transplant (n = 2823)	
	Not hospitalized (n = 53 539)	Hospitalized (n = 6917)	Not hospitalized (n = 36 184)	Hospitalized (n = 5279)	Not hospitalized(n = 13 539)	Hospitalized (n = 925)	Not hospitalized (n = 4640)	Hospitalized (n = 687)	Not hospitalized (n = 2312)	Hospitalized (n = 511)
Age, mean (SD), y	55.7 (15.7)	58.2 (14)	55.8 (15.1)	58.5 (13.7)	56.3 (17)	60.4 (14)	55 (16.2)	56.5 (14.9)	50.3 (15.1)	50.8 (14.2)
Age, median (IQR), y	58 (47-67)	60 (50-68)	58 (46-67)	60 (50-69)	61 (50-68)	64 (54-70)	58 (46-67)	59 (47-68)	53 (38-63)	54 (39-62)
Age group, y										
1-45	12 467 (23.3)	1258 (18.2)	8621 (23.8)	925 (17.5)	2719 (20.1)	130 (14.1)	1147 (24.7)	152 (22.1)	851 (36.8)	174 (34.1)
46-65	24 852 (46.4)	3319 (48.0)	16 805 (46.4)	2557 (48.4)	6198 (45.8)	405 (43.8)	2148 (46.3)	317 (46.1)	1082 (46.8)	260 (50.9)
≥66	16 220 (30.3)	2340 (33.8)	10 758 (29.7)	1797 (34.0)	4622 (34.1)	390 (42.2)	1345 (29.0)	218 (31.7)	379 (16.4)	77 (15.1)
Age category, y										
1-17	1253 (2.3)	37 (0.5)	506 (1.4)	19 (0.4)	678 (5.0)	9 (1.0)	127 (2.7)	8 (1.2)	33 (1.4)	3 (0.6)
18-29	2443 (4.6)	221 (3.2)	1501 (4.1)	131 (2.5)	630 (4.7)	29 (3.1)	260 (5.6)	33 (4.8)	221 (9.6)	48 (9.4)
30-49	12 345 (23.1)	1451 (21.0)	9302 (25.7)	1130 (21.4)	2032 (15.0)	137 (14.8)	1054 (22.7)	163 (23.7)	753 (32.6)	158 (30.9)
50-59	12 371 (23.1)	1616 (23.4)	8598 (23.8)	1275 (24.2)	2898 (21.4)	161 (17.4)	1063 (22.9)	146 (21.3)	523 (22.6)	140 (27.4)
60-69	14 999 (28.0)	2091 (30.2)	9249 (25.6)	1523 (28.9)	4608 (34.0)	351 (37.9)	1329 (28.6)	208 (30.3)	620 (26.8)	135 (26.4)
70-79	8673 (16.2)	1258 (18.2)	5918 (16.4)	1002 (19.0)	2419 (17.9)	206 (22.3)	681 (14.7)	106 (15.4)	152 (6.6)	26 (5.1)
≥80	1455 (2.7)	243 (3.5)	1110 (3.1)	199 (3.8)	274 (2.0)	32 (3.5)	126 (2.7)	23 (3.3)	10 (0.4)	1 (0.2)
Sex										
Male	34 173 (63.8)	4316 (62.4)	22 373 (61.8)	3197 (60.6)	9103 (61.2)	638 (69.0)	3411 (73.5)	505 (73.5)	1213 (52.5)	228 (53.4)
Female	19 366 (36.2)	2601 (37.6)	13 811 (38.2)	2082 (39.4)	4436 (32.8)	287 (31.0)	1229 (26.5)	182 (26.5)	1099 (47.5)	238 (46.6)
Region of residence										
Île-de-France	9692 (18.1)	1902 (27.5)	6427 (17.8)	1517 (28.7)	2704 (20.0)	221 (23.9)	769 (16.6)	169 (24.6)	377 (16.3)	119 (23.3)
Northwest	12 035 (22.5)	1033 (14.9)	8133 (22.5)	777 (14.7)	2854 (21.1)	121 (13.1)	1177 (25.4)	116 (16.9)	485 (21.0)	89 (17.4)
Northeast	10 544 (19.7)	1587 (22.9)	6923 (19.1)	1141 (21.6)	2664 (19.7)	204 (22.1)	974 (21.0)	196 (28.5)	587 (25.4)	157 (30.7)
Southeast	13 501 (25.2)	1590 (23.0)	9041 (25.0)	1208 (22.9)	3576 (26.4)	272 (29.4)	1135 (24.5)	121 (17.6)	568 (24.6)	97 (19.0)
Southwest	7767 (14.5)	805 (11.6)	5660 (15.6)	636 (12.0)	1741 (12.9)	107 (11.6)	585 (12.6)	85 (12.4)	295 (12.8)	49 (9.6)
Social Deprivation Index quintile										
1 (Least deprived)	9590 (17.9)	1319 (19.1)	6380 (17.6)	995 (18.8)	2521 (18.6)	182 (19.7)	859 (18.5)	131 (19.1)	444 (19.2)	107 (20.9)
2	10 333 (19.3)	1221 (17.7)	6997 (19.3)	916 (17.4)	2696 (19.9)	182 (19.7)	848 (18.3)	135 (19.7)	473 (20.5)	94 (18.4)
3	10 541 (19.7)	1211 (17.5)	7259 (20.1)	915 (17.3)	2529 (18.7)	171 (18.5)	880 (19.0)	117 (17.0)	460 (19.9)	84 (16.4)
4	10 716 (20.0)	1259 (18.2)	7170 (19.8)	966 (18.3)	2704 (20.0)	172 (18.6)	976 (21.0)	121 (17.6)	472 (20.4)	88 (17.2)
5 (Most deprived)	10 620 (19.8)	1573 (22.7)	7098 (19.6)	1216 (23.0)	2715 (20.1)	181 (19.6)	942 (20.3)	149 (21.7)	424 (18.3)	125 (24.5)
Unknown	1739 (3.2)	334 (4.8)	1280 (3.5)	271 (5.1)	374 (2.8)	37 (4.0)	135 (2.9)	34 (4.9)	39 (1.7)	13 (2.5)
Comorbidities										
Alcohol	9279 (17.3)	795 (11.5)	2079 (5.7)	313 (5.9)	7258 (53.6)	456 (49.3)	480 (10.3)	74 (10.8)	224 (9.7)	52 (10.2)
Smoking	12 295 (23.0)	1550 (22.4)	6847 (18.9)	975 (18.5)	3936 (29.1)	245 (26.5)	1431 (30.8)	225 (32.8)	964 (41.7)	250 (48.9)
Obesity	10 434 (19.5)	1777 (25.7)	6481 (17.9)	1352 (25.6)	3272 (24.2)	258 (27.9)	963 (20.8)	191 (27.8)	305 (13.2)	92 (18.0)
Hypertension	40 452 (75.6)	5694 (82.3)	29304 (81.0)	4536 (85.9)	8383 (61.9)	674 (72.9)	3631 (78.3)	561 (81.7)	1390 (60.1)	301 (58.9)
Cardiovascular^b^	16 517 (30.9)	2805 (40.6)	9823 (27.1)	1908 (36.1)	3201 (23.6)	337 (36.4)	3974 (85.6)	615 (89.5)	811 (35.1)	200 (39.1)
Statin use	20 352 (38.0)	3092 (44.7)	14773 (40.8)	2357 (44.6)	2928 (21.6)	286 (30.9)	3189 (68.7)	514 (74.8)	631 (27.3)	154 (30.1)
Diabetes	14 732 (27.5)	2751 (39.8)	9440 (26.1)	2091 (39.6)	4547 (33.6)	473 (51.1)	1084 (23.4)	217 (31.6)	840 (36.3)	214 (41.9)
Dialysis	185 (0.3)	55 (0.8)	0	0	83 (0.6)	21 (2.3)	83 (1.8)	30 (4.4)	25 (1.1)	9 (1.8)
Respiratory^c^	5592 (10.4)	1038 (15.0)	2824 (7.8)	597 (11.3)	1475 (10.9)	128 (13.8)	586 (12.6)	108 (15.7)	1171 (50.6)	303 (59.3)
Cancer	4968 (9.3)	543 (7.9)	2808 (7.8)	374 (7.1)	1896 (14.0)	111 (12.0)	399 (8.6)	62 (9.0)	228 (9.9)	42 (8.2)
Psychiatric	2835 (5.3)	397 (5.7)	1456 (4.0)	254 (4.8)	1003 (7.4)	72 (7.8)	368 (7.9)	54 (7.9)	230 (9.9)	51 (10.0)
Antidepressant use	3197 (6.0)	490 (7.1)	2053 (5.7)	341 (6.5)	795 (5.9)	73 (7.9)	357 (7.7)	54 (7.9)	242 (10.5)	63 (12.3)
Anxiolytic use	4931 (9.2)	718 (10.4)	3022 (8.4)	491 (9.3)	1391 (10.3)	99 (10.7)	566 (12.2)	101 (14.7)	283 (12.2)	85 (16.6)
No. of consultations (within 6 mo before index date)										
0	4386 (8.2)	541 (7.8)	2795 (7.7)	397 (7.5)	1170 (8.6)	65 (7.0)	471 (10.2)	74 (10.8)	255 (11)	41 (8.0)
1-3	20 687 (38.6)	2425 (35.1)	13 699 (37.9)	1799 (34.1)	5191 (38.3)	314 (33.9)	1964 (42.3)	273 (39.7)	1010 (43.7)	204 (39.9)
4-6	14 880 (27.8)	1822 (26.3)	10140 (28.0)	1402 (26.6)	3683 (27.2)	232 (25.1)	1243 (26.8)	173 (25.2)	636 (27.5)	144 (28.2)
≥7	13 586 (25.4)	2129 (30.8)	9550 (26.4)	1681 (31.8)	3495 (25.8)	314 (33.9)	962 (20.7)	167 (24.3)	411 (17.8)	122 (23.9)
No. of hospitalizations (within 6 mo before index date)										
0	40 933 (76.5)	4692 (67.8)	28 018 (77.4)	3660 (69.3)	10 379 (76.7)	623 (67.4)	3318 (71.5)	431 (62.7)	1371 (59.3)	275 (53.8)
1-3	11 204 (20.9)	1936 (28.0)	7224 (20.0)	1412 (26.7)	2825 (20.9)	256 (27.7)	1202 (25.9)	226 (32.9)	792 (34.3)	192 (37.6)
4-6	1078 (2.0)	225 (3.3)	707 (2.0)	168 (3.2)	269 (2.0)	33 (3.6)	87 (1.9)	19 (2.8)	121 (5.2)	34 (6.7)
≥7	324 (0.6)	64 (0.9)	235 (0.6)	39 (0.7)	66 (0.5)	13 (1.4)	33 (0.7)	11 (1.6)	28 (1.2)	10 (2.0)
Time since transplant, y										
<2	7913 (14.8)	1258 (18.2)	5085 (14.1)	929 (17.6)	2050 (15.1)	150 (16.2)	532 (11.5)	95 (13.8)	434 (18.8)	123 (24.1)
2-5	11 939 (22.3)	1734 (25.1)	7810 (21.6)	1284 (24.3)	3074 (22.7)	209 (22.6)	929 (20.0)	168 (24.5)	647 (28.0)	164 (32.1)
5-10	15 062 (28.1)	1896 (27.4)	9927 (27.4)	1463 (27.7)	3989 (29.5)	263 (28.4)	1307 (28.2)	173 (25.2)	741 (32.1)	136 (26.6)
≥10	18 625 (34.8)	2029 (29.3)	13362 (36.9)	1603 (30.4)	4426 (32.7)	303 (32.8)	1872 (40.3)	251 (36.5)	490 (21.2)	88 (17.2)

^a^ Data are presented as number (percentage) of patients unless otherwise indicated. Some patients received more than 1 transplant.

^b^ Except for heart transplant.

^c^ Except for mucoviscidosis and lung transplant.

**Table 2.** Description of Reimbursements for Immunosuppressants Among Solid Organ Transplant Recipients According to COVID-19 Hospitalization Groups

Immunosuppressant	No. (%) of patients									
	Whole transplant cohort (n = 60 456)		Kidney transplant (n = 41 463)		Liver transplant (n = 14 464)		Heart transplant (n = 5327)		Lung transplant (n = 2823)	
	Not hospitalized (n = 53 539)	Hospitalized (n = 6917)	Not hospitalized (n = 36 183)	Hospitalized (n = 5279)	Not hospitalized (n = 13 539)	Hospitalized (n = 925)	Not hospitalized (n = 4640)	Hospitalized (n = 687)	Not hospitalized (n = 2312)	Hospitalized (n = 511)
Steroids	31 675 (59.2)	5251 (75.9)	24 378 (67.4)	4187 (79.3)	3793 (28.0)	372 (40.2)	3634 (78.3)	588 (85.6)	1966 (85.0)	471 (92.2)
Cumulative DDD for prednisolone										
0	21 864 (40.8)	1666 (24.1)	11 806 (32.6)	1092 (20.7)	9746 (72.0)	553 (59.8)	1006 (21.7)	99 (14.4)	346 (15.0)	40 (7.8)
1-75	8467 (15.8)	1122 (16.2)	6299 (17.4)	862 (16.3)	1570 (11.6)	120 (13.0)	750 (16.2)	122 (17.8)	457 (19.8)	98 (19.2)
76-99	7709 (14.4)	1195 (17.3)	6373 (17.6)	1018 (19.3)	794 (5.9)	77 (8.3)	658 (14.2)	92 (13.4)	381 (16.5)	95 (18.6)
100-150	8142 (15.2)	1519 (22.0)	6277 (17.3)	1243 (23.5)	757 (5.6)	98 (10.6)	1179 (25.4)	188 (27.4)	488 (21.1)	107 (20.9)
≥151	7357 (13.7)	1415 (20.5)	5429 (15.0)	1064 (20.2)	672 (5.0)	77 (8.3)	1047 (22.6)	186 (27.1)	640 (27.7)	171 (33.5)
Tacrolimus	37504 (70.0)	4850 (70.1)	25218 (69.7)	3714 (70.4)	10418 (76.9)	676 (73.1)	2266 (48.8)	348 (50.7)	1980 (85.6)	458 (89.6)
Cyclosporine	11223 (21.0)	1455 (21.0)	7804 (21.6)	1111 (21.0)	1438 (10.6)	99 (10.7)	2204 (47.5)	290 (42.2)	307 (13.3)	45 (8.8)
Mycophenolic acid	38476 (71.9)	5495 (79.4)	27049 (74.8)	4254 (80.6)	8321 (61.5)	669 (72.3)	3566 (76.9)	543 (79.0)	1640 (70.9)	395 (77.3)
Cumulative DDD for mycophenolic acid										
0	15 063 (28.1)	1422 (20.6)	9135 (25.2)	1025 (19.4)	5218 (38.5)	256 (27.7)	1074 (23.1)	144 (21.0)	672 (29.1)	116 (22.7)
1-65	11 010 (20.6)	1388 (20.1)	8316 (23.0)	1141 (21.6)	2202 (16.3)	174 (18.8)	790 (17.0)	113 (16.4)	408 (17.6)	74 (14.5)
66-100	11 522 (21.5)	1523 (22.0)	8429 (23.3)	1257 (23.8)	2597 (19.2)	193 (20.9)	759 (16.4)	103 (15.0)	373 (16.1)	72 (14.1)
101-140	7142 (13.3)	1162 (16.8)	5180 (14.3)	947 (17.9)	1384 (10.2)	109 (11.8)	636 (13.7)	98 (14.0)	299 (12.9)	76 (14.9)
≥141	8802 (16.4)	1422 (20.6)	5124 (14.2)	909 (17.2)	2138 (15.8)	193 (20.9)	1381 (29.8)	231 (33.6)	560 (24.2)	173 (33.9)
Azathioprine	3763 (7.0)	398 (5.8)	3231 (8.9)	343 (6.5)	328 (2.4)	20 (2.2)	174 (3.8)	28 (4.1)	225 (9.7)	34 (6.7)
Sirolimus	1228 (2.3)	135 (2.0)	1035 (2.9)	109 (2.1)	209 (1.5)	23 (2.5)	25 (0.5)	10 (1.5)	18 (0.8)	6 (1.2)
Everolimus	6233 (11.6)	769 (11.1)	2887 (8.0)	393 (7.4)	2113 (15.6)	129 (13.9)	1313 (28.3)	222 (32.3)	429 (18.6)	97 (19.0)
Treatment regimen										
Tacrolimus, mycophenolic acid, and steroids	15 330 (28.6)	2815 (40.7)	12 324 (34.1)	2326 (44.1)	1816 (13.4)	193 (20.9)	1111 (23.9)	189 (27.5)	1071 (46.3)	303 (59.3)
Tacrolimus and mycophenolic acid	10 487 (19.6)	854 (12.3)	6277 (17.3)	595 (11.3)	4172 (30.8)	268 (29.0)	305 (6.6)	25 (3.6)	187 (8.1)	20 (3.9)
Cyclosporine, mycophenolic acid, and steroids	4124 (7.7)	720 (10.4)	2973 (8.2)	566 (10.7)	190 (1.4)	38 (4.1)	1032 (22.2)	138 (20.1)	156 (6.7)	27 (5.3)
Cyclosporine and mycophenolic acid	2844 (5.3)	290 (4.2)	2127 (5.9)	245 (4.6)	506 (3.7)	24 (2.6)	276 (5.9)	28 (4.1)	28 (1.2)	4 (0.8)
Tacrolimus only	3000 (5.6)	127 (1.8)	693 (1.9)	46 (0.9)	2441 (18.0)	86 (9.3)	36 (0.8)	3 (0.4)	33 (1.4)	2 (0.4)
Tacrolimus and steroids	2692 (5.0)	337 (4.9)	1927 (5.3)	271 (5.1)	735 (5.4)	54 (5.8)	147 (3.2)	17 (2.5)	124 (5.4)	15 (2.9)
Tacrolimus, sirolimus-everolimus, and steroids	1487 (2.8)	229 (3.3)	1012 (2.8)	144 (2.7)	217 (1.6)	16 (1.7)	198 (4.3)	43 (6.3)	211 (9.1)	53 (10.4)
Tacrolimus, azathioprine, and steroids	1400 (2.6)	163 (2.4)	1184 (3.3)	135 (2.6)	121 (0.9)	7 (0.8)	59 (1.3)	11 (1.6)	138 (6.0)	23 (4.5)
Cyclosporine and steroids	1288 (2.4)	130 (1.9)	1020 (2.8)	109 (2.1)	139 (1.0)	6 (0.6)	161 (3.5)	20 (2.9)	31 (1.3)	4 (0.8)
Mycophenolic acid and steroids	1027 (1.9)	212 (3.1)	952 (2.6)	201 (3.8)	89 (0.7)	14 (1.5)	12 (0.3)	9 (1.3)	7 (0.3)	1 (0.2)
Sirolimus-everolimus and mycophenolic acid	1081 (2.0)	77 (1.1)	440 (1.2)	30 (0.6)	636 (4.7)	46 (5.0)	59 (1.3)	9 (1.3)	3 (0.1)	1 (0.2)
Sirolimus-everolimus, mycophenolic acid, and steroids	938 (1.8)	157 (2.3)	750 (2.1)	115 (2.2)	115 (0.8)	16 (1.7)	113 (2.4)	32 (4.7)	15 (0.6)	3 (0.6)
Others	7841 (14.6)	806 (11.7)	4505 (12.5)	496 (9.4)	2362 (17.4)	157 (17.0)	1131 (24.4)	163 (23.7)	308 (13.3)	55 (10.8)
Treatment regimen classes										
Treatment combinations										
CNI, antimetabolites, and steroids	21 814 (40.7)	3831 (55.4)	17 305 (47.8)	3150 (59.7)	2173 (16.0)	239 (25.8)	2289 (49.3)	347 (50.5)	1413 (61.1)	358 (70.1)
CNI, mTORi, and steroids	1846 (3.4)	274 (4.0)	1174 (3.2)	168 (3.2)	229 (1.7)	19 (2.1)	386 (8.3)	62 (9.0)	234 (10.1)	57 (11.2)
Antimetabolite, mTORi, and steroids	998 (1.9)	161 (2.3)	796 (2.2)	118 (2.2)	126 (0.9)	16 (1.7)	117 (2.5)	34 (4.9)	17 (0.7)	3 (0.6)
CNI, antimetabolite, and mTORi	488 (0.9)	36 (0.5)	110 (0.3)	7 (0.1)	238 (1.8)	12 (1.3)	131 (2.8)	17 (2.5)	32 (1.4)	4 (0.8)
CNI and antimetabolites	14 369 (26.8)	1214 (17.6)	9286 (25.7)	900 (17.0)	4803 (35.5)	300 (32.4)	622 (13.4)	58 (8.4)	245 (10.6)	26 (5.1)
CNI and mTORi	919 (1.7)	69 (1.0)	427 (1.2)	41 (0.8)	430 (3.2)	22 (2.4)	118 (2.5)	10 (1.5)	20 (0.9)	5 (1.0)
CNI and steroids	3991 (7.5)	467 (6.8)	2952 (8.2)	380 (7.2)	877 (6.5)	60 (6.5)	311 (6.7)	37 (5.4)	155 (6.7)	19 (3.7)
Antimetabolite and steroids	1360 (2.5)	254 (3.7)	1273 (3.5)	241 (4.6)	102 (0.8)	16 (1.7)	15 (0.3)	9 (1.3)	9 (0.4)	2 (0.4)
Antimetabolite and mTORi	1109 (2.1)	81 (1.2)	463 (1.3)	34 (0.6)	641 (4.7)	46 (5.0)	59 (1.3)	9 (1.3)	3 (0.1)	1 (0.2)
mTORi and steroids	523 (1.0)	65 (0.9)	414 (1.1)	58 (1.1)	110 (0.8)	10 (1.1)	17 (0.4)	2 (0.3)	16 (0.7)	1 (0.2)
CNI	3883 (7.3)	170 (2.5)	1116 (3.1)	68 (1.3)	2903 (21.4)	110 (11.9)	63 (1.4)	4 (0.6)	42 (1.8)	2 (0.4)
Antimetabolite	666 (1.2)	78 (1.1)	333 (0.9)	39 (0.7)	361 (2.7)	48 (5.2)	2 (0.0)	0 (0.0)	2 (0.1)	1 (0.2)
mTORi	430 (0.8)	18 (0.3)	71 (0.2)	3 (0.1)	370 (2.7)	15 (1.6)	11 (0.2)	1 (0.1)	2 (0.1)	1 (0.2)
Others	1143 (2.1)	199 (2.9)	464 (1.3)	72 (1.4)	176 (1.3)	12 (1.3)	499 (10.8)	97 (14.1)	122 (5.3)	31 (6.1)
Immunosuppression intensity (including steroids)										
Single regimen	4979 (9.3)	266 (3.8)	1520 (4.2)	110 (2.1)	3634 (26.8)	173 (18.7)	76 (1.6)	5 (0.7)	46 (2.0)	4 (0.8)
Double regimen	22 271 (41.6)	2150 (31.1)	14815 (40.9)	1654 (31.3)	6963 (51.4)	454 (49.1)	1142 (24.6)	125 (18.2)	448 (19.4)	54 (10.6)
Triple regimen	26 289 (49.1)	4501 (65.1)	19 849 (54.9)	3515 (66.6)	2942 (21.7)	298 (32.2)	3422 (73.8)	557 (81.1)	1818 (78.6)	453 (88.6)

Abbreviations: CNI, calcineurin inhibitor; DDD, defined daily dose; mTORi, mammalian target of rapamycin inhibitors.

The AORs of factors associated with the risk of hospitalization for COVID-19 are displayed in Table 3 and Table 4. Crude odds ratios are displayed in eTables 5, 7, 10, and 12 in Supplement 1. In kidney transplant recipients, the factors associated with an increased risk of hospitalization included age older than 66 years vs 1 to 45 years (AOR, 1.40; 95% CI, 1.28-1.55), high Social Deprivation Index (fifth vs first quintile: AOR, 1.34; 95% CI, 1.21-1.48), obesity (AOR, 1.19; 95% CI, 1.10-1.28), hypertension (AOR, 1.14; 95% CI, 1.04-1.24), diabetes (AOR, 1.46; 95% CI, 1.36-1.55), steroids (AOR, 1.60; 95% CI, 1.49-1.73), and mycophenolic acid (AOR, 1.37; 95% CI, 1.25-1.51). Compared with patients who had a transplant less than 2 years ago, those with a transplant of 2 to 5 years (AOR, 1.06; 95% CI, 0.96-1.17) or 5 to 10 years (AOR, 1.00; 95% CI, 0.90-1.10) were not different, but those with a transplant of more than 10 years had a lower risk of hospitalization (AOR, 0.86; 95% CI, 0.78-0.95). In liver transplant recipients, the factors associated with an increased risk of hospitalization included age older than 66 years vs 1 to 45 years (AOR, 1.42; 95% CI, 1.11-1.82), hypertension (AOR, 1.21; 95% CI, 1.02-1.43), diabetes (AOR, 1.70; 95% CI, 1.47-1.98), dialysis (AOR, 2.85; 95% CI, 1.67-4.85), steroids (AOR, 1.60; 95% CI, 1.38-1.86), and mycophenolic acid (AOR, 1.61; 95% CI, 1.37-1.90). Tacrolimus (AOR, 0.77; 95% CI, 0.61-0.98) was associated with a decreased risk of hospitalization. In heart transplant recipients, cyclosporine (AOR, 0.67; 95% CI, 0.47-0.94) was associated with a decreased risk of hospitalization. Obesity (AOR, 1.25; 95% CI, 1.02-1.52), diabetes (AOR, 1.28; 95% CI, 1.06-1.56), dialysis (AOR, 2.17; 95% CI, 1.37-3.46), steroids (AOR, 1.42; 95% CI, 1.11-1.82), mycophenolic acid (AOR, 1.29; 95% CI, 1.02-1.64), sirolimus (AOR, 2.71; 95% CI, 1.20-6.09), and everolimus (AOR, 1.24; 95% CI, 1.01-1.51) were associated with an increased risk of hospitalization. Only steroids (AOR, 1.72; 95% CI, 1.19-2.48) were associated with a high risk of COVID-19 hospitalization in lung transplant recipients.

**Table 3.** Identification of Factors Associated With COVID-19–Related Hospitalization in Multivariable Models Among Solid Organ Transplant Recipients

	AOR (95% CI)^a^				
	Whole cohort	Kidney transplant	Liver transplant	Heart transplant	Lung transplant
Age group, y					
1-45	1 [Reference]	1 [Reference]	1 [Reference]	1 [Reference]	1 [Reference]
46-65	1.23 (1.14-1.33)	1.30 (1.20-1.42)	1.22 (0.95-1.50)	0.97 (0.77-1.21)	0.87 (0.67-1.15)
≥66	1.35 (1.24-1.47)	1.40 (1.28-1.55)	1.42 (1.11-1.82)	1.17 (0.91-1.51)	0.75 (0.52-1.07)
Sex					
Male	1 [Reference]	1 [Reference]	1 [Reference]	1 [Reference]	1 [Reference]
Female	1.03 (0.98-1.09)	1.04 (0.98-1.11)	0.94 (0.81-1.11)	1.06 (0.87-1.30)	0.96 (0.78-1.18)
Social Deprivation Index quintile					
1 (Least deprived)	1 [Reference]	1 [Reference]	1 [Reference]	1 [Reference]	1 [Reference]
2	1.05 (0.96-1.14)	1.06 (0.96-1.17)	1.01 (0.81-1.27)	1.16 (0.88-1.53)	0.94 (0.67-1.31)
3	1.09 (0.99-1.19)	1.10 (0.99-1.22)	1.07 (0.85-1.36)	1.10 (0.82-1.47)	0.94 (0.66-1.34)
4	1.13 (1.03-1.24)	1.20 (1.08-1.33)	1.01 (0.80-1.29)	0.96 (0.72-1.28)	0.89 (0.62-1.26)
5 (Most deprived)	1.27 (1.16-1.38)	1.34 (1.21-1.48)	1.05 (0.83-1.33)	1.03 (0.78-1.37)	1.27 (0.91-1.79)
Comorbidities					
Alcohol	0.66 (0.60-0.71)	0.90 (0.79-1.03)	0.72 (0.61-0.84)	0.96 (0.73-1.27)	0.92 (0.66-1.30)
Smoking	0.87 (0.81-0.93)	0.84 (0.78-0.91)	0.79 (0.67-0.94)	0.94 (0.78-1.15)	1.16 (0.91-1.49)
Obesity	1.16 (1.09-1.26)	1.19 (1.10-1.28)	0.98 (0.83-1.15)	1.25 (1.02-1.52)	1.23 (0.93-1.62)
Hypertension	1.17 (1.09-1.26)	1.14 (1.04-1.24)	1.21 (1.02-1.43)	1.12 (0.90-1.40)	0.97 (0.78-1.21)
Cardiovascular^b^	1.21 (1.14-1.28)	1.21 (1.13-1.30)	1.37 (1.17-1.61)	1.19 (0.90-1.56)	1.06 (0.85-1.33)
Statin use	1.04 (0.98-1.10)	0.97 (0.91-1.03)	1.17 (0.99-1.38)	1.22 (1.01-1.48)	1.08 (0.85-1.36)
Diabetes	1.46 (1.38-1.55)	1.46 (1.36-1.55)	1.70 (1.47-1.98)	1.28 (1.06-1.56)	1.14 (0.92-1.41)
Dialysis	2.13 (1.55-2.93)	NA	2.85 (1.67-4.85)	2.17 (1.37-3.46)	1.57 (0.67-3.63)
Respiratory^c^	1.31 (1.21-1.41)	1.28 (1.16-1.41)	1.13 (0.92-1.39)	1.11 (0.87-1.41)	1.30 (0.99-1.69)
Cancer	0.84 (0.76-0.93)	0.90 (0.80-1.01)	0.72 (0.57-0.90)	1.01 (0.75-1.37)	0.80 (0.55-1.16)
Psychiatric	1.11 (0.99-1.25)	1.19 (1.03-1.37)	1.08 (0.83-1.40)	0.91 (0.66-1.25)	0.88 (0.62-1.25)
Antidepressant use	1.11 (0.99-1.23)	1.08 (0.95-1.22)	1.23 (0.95-1.60)	0.86 (0.63-1.19)	1.06 (0.77-1.47)
Anxiolytic use	1.08 (0.99-1.18)	1.06 (0.95-1.18)	0.97 (0.77-1.22)	1.26 (0.98-1.61)	1.30 (0.98-1.74)
No. of consultations (within 6 mo before index date)					
0	1 [Reference]	1 [Reference]	1 [Reference]	1 [Reference]	1 [Reference]
1-3	1.09 (0.99-1.21)	1.11 (0.98-1.25)	1.19 (0.89-1.58)	0.90 (0.67-1.20)	1.25 (0.86-1.81)
4-6	1.10 (0.99-1.23)	1.15 (1.01-1.30)	1.14 (0.84-1.53)	0.88 (0.64-1.20)	1.40 (0.94-2.07)
≥7	1.26 (1.12-1.40)	1.29 (1.13-1.47)	1.49 (1.10-2.01)	0.94 (0.68-1.30)	1.92 (1.28-2.90)
No. of hospitalizations (within 6 mo before index date)					
0	1 [Reference]	1 [Reference]	1 [Reference]	1 [Reference]	1 [Reference]
1-3	1.21 (1.13-1.29)	1.18 (1.10-1.27)	1.20 (1.02-1.43)	1.24 (1.03-1.49)	1.06 (0.85-1.33)
4-6	1.33 (1.14-1.55)	1.31 (1.10-1.58)	1.52 (1.02-2.26)	1.20 (0.69-2.07)	1.14 (0.74-1.75)
≥7	1.30 (0.98-1.72)	0.99 (0.70-1.42)	1.80 (0.94-3.46)	2.02 (0.96-4.27)	1.52 (0.67-3.43)
Time since transplant, y					
<2	1 [Reference]	1 [Reference]	1 [Reference]	1 [Reference]	1 [Reference]
2-5	1.10 (1.01-1.19)	1.06 (0.96-1.17)	1.07 (0.84-1.35)	1.09 (0.81-1.45)	0.95 (0.71-1.27)
5-10	1.01 (0.93-1.09)	1.00 (0.90-1.10)	1.01 (0.80-1.27)	0.86 (0.64-1.15)	0.78 (0.57-1.07)
≥10	0.90 (0.83-0.98)	0.86 (0.78-0.95)	0.97 (0.76-1.23)	0.88 (0.66-1.18)	0.84 (0.58-1.20)

Abbreviations: AOR, adjusted odds ratio; NA, not applicable.

^a^ Adjusted for tacrolimus, cyclosporine, mycophenolic acid, azathioprine, sirolimus, everolimus, and region of residence.

^b^ Except for heart transplantation.

^c^ Except for mucoviscidose and lung transplantation.

**Table 4.** Association of Immunosuppressive Drugs and Risk of Hospitalization for COVID-19 in Multivariable Models Among Solid Organ Transplant Recipients

	AOR (95% CI)				
	Whole cohort	Kidney transplant	Liver transplant	Heart transplant	Lung transplant
Immunosuppressive drugs^a^					
Steroids	1.80 (1.69-1.92)	1.60 (1.49-1.73)	1.60 (1.38-1.86)	1.42 (1.11-1.82)	1.72 (1.19-2.48)
Tacrolimus	0.99 (0.89-1.09)	0.97 (0.86-1.08)	0.77 (0.61-0.98)	0.86 (0.60-1.22)	0.89 (0.48-1.63)
Cyclosporine	0.96 (0.87-1.07)	1.01 (0.89-1.14)	0.82 (0.61-1.12)	0.67 (0.47-0.94)	0.64 (0.34-1.22)
Mycophenolic acid	1.48 (1.37-1.59)	1.37 (1.25-1.51)	1.61 (1.37-1.90)	1.29 (1.02-1.64)	1.33 (0.96-1.82)
Azathioprine	0.99 (0.88-1.12)	0.90 (0.78-1.03)	0.98 (0.61-1.59)	1.34 (0.84-2.13)	0.79 (0.50-1.25)
Sirolimus	0.92 (0.75-1.12)	0.81 (0.65-1.02)	1.15 (0.71-1.87)	2.71 (1.20-6.09)	1.76 (0.62-4.96)
Everolimus	1.01 (0.92-1.10)	0.93 (0.82-1.05)	0.82 (0.64-1.05)	1.24 (1.01-1.51)	1.16 (0.85-1.58)
Cumulative DDD for prednisolone^b^					
0	1 [Reference]	1 [Reference]	1 [Reference]	1 [Reference]	1 [Reference]
1-75	1.51 (1.39-1.64)	1.30 (1.18-1.44)	1.33 (1.08-1.65)	1.42 (1.05-1.91)	1.58 (1.04-2.40)
76-99	1.71 (1.58-1.86)	1.50 (1.36-1.65)	1.64 (1.26-2.13)	1.18 (0.86-1.63)	1.85 (1.21-2.83)
100-150	2.03 (1.88-2.20)	1.86 (1.69-2.03)	1.93 (1.51-2.46)	1.51 (1.15-1.99)	1.54 (1.01-2.33)
≥151	2.00 (1.84-2.17)	1.80 (1.63-1.98)	1.84 (1.41-2.41)	1.49 (1.12-1.99)	1.93 (1.28-2.91)
Cumulative DDD for mycophenolic acid^c^					
0	1 [Reference]	1 [Reference]	1 [Reference]	1 [Reference]	1 [Reference]
1-65	1.25 (1.13-1.39)	1.54 (1.24-1.90)	1.24 (0.92-1.66)	1.17 (0.80-1.71)	1.25 (1.13-1.39)
66-100	1.33 (1.20-1.48)	1.51 (1.23-1.86)	1.14 (0.84-1.55)	1.13 (0.76-1.68)	1.33 (1.20-1.48)
101-140	1.60 (1.43-1.80)	1.57 (1.22-2.01)	1.31 (0.96-1.80)	1.42 (0.95-2.13)	1.60 (1.43-1.80)
≥141	1.51 (1.34-1.69)	1.94 (1.56-2.41)	1.45 (1.10-1.91)	1.65 (1.15-2.38)	1.51 (1.34-1.69)

Abbreviations: AOR, adjusted odds ratio; DDD, defined daily dose.

^a^ Adjusted for age, sex, Social Deprivation Index, each of the comorbidities, number of consultations, number of hospitalizations, time since transplant, and region of residence.

^b^ Adjusted for age, sex, Social Deprivation Index, each of the comorbidities, number of consultations, number of hospitalizations, time since transplant, region of residence, tacrolimus, cyclosporin, mycophenolic acid, azathioprine, sirolimus, and everolimus.

^c^ Adjusted for age, sex, Social Deprivation Index, each of the comorbidities, number of consultations, number of hospitalizations, time since transplant, region of residence, tacrolimus, cyclosporin, azathioprine, sirolimus, everolimus, and steroids.

The AOR of the main analysis with steroids and mycophenolic acid doses showed a dose-related association with the risk of hospitalization (Table 4). Consistent with the main analysis, sensitivity analyses showed an increased risk of hospitalization associated with obesity, diabetes, steroids, and mycophenolic acid use (eTables 13-16 in Supplement 1). In an exploratory analysis (treatment regimen types and treatment intensity), low immunosuppression was associated with a reduced risk of hospitalization (eTables 8 and 17 in Supplement 1).

## Discussion

This cohort study analyzed 60 456 SOTRs receiving immunosuppressive therapy, of whom 11.4% were hospitalized for COVID-19. We observed an increased risk of COVID-19 hospitalization associated with using sirolimus in heart transplant recipients and using mycophenolic acid in kidney and liver transplant recipients independent of other drugs. Our study is the first, to our knowledge, to show this sirolimus association with COVID-19 hospitalization. Steroids were also associated with an increased risk of hospitalization for COVID-19 in each transplant subgroup. Tacrolimus and cyclosporine were conversely associated with a low risk of hospitalization in liver and heart transplant recipients, respectively.

The pharmacologic mechanism of action of these drugs may explain these results. Both mycophenolic acid and SARS-CoV-2 have a cytostatic effect, notably on lymphocytes for the mycophenolic acid.^21,22,23^ These synergistic effects lead to significant lymphopenia, which reduces the immune response and may worsen disease outcomes.^24,25^ This effect has been demonstrated with other viruses.^26^ In 2020, several experts in solid organ transplantation recommended reducing or stopping mycophenolate treatment in transplant patients hospitalized for COVID-19.^27,28^ As in our study, a Spanish study^29^ and a Danish study^30^ found an increased risk of mortality and hospitalization associated with long-term use of steroids. Glucocorticoids have also been reported to most often facilitate various infections.^22,31^ Tacrolimus has the effect of suppressing the early phase of T-cell activation, reducing the production of cytokines (including proinflammatory cytokines). This mechanism could thus mitigate the cytokine storm that characterizes severe COVID-19.^32^ Belli et al,^33^ in their study of liver transplant recipients with COVID-19, reported that using tacrolimus was associated with better survival.^34^

We found an inverse association between a longer time since transplant (>10 years) and the risk of being hospitalized for COVID-19 in kidney transplant patients. The hypothesis underlying this result would be that a very high level of immunosuppression is recommended during at least the first year after transplant (to reduce the risk of acute rejection), after which the aim is to decrease the intensity of the drugs in the direction of a less potent treatment. A description of immunosuppressive drugs according to the time since transplant is presented in eFigure 2 in Supplement 1 and is consistent with our hypothesis. We also identified increasing age, obesity, hypertension, diabetes, dialysis, and chronic respiratory disease as risk factors associated with hospitalization for COVID-19 among SOTRs, as in the general population.^2,35^

### Strengths and Limitations

Our study has several strengths. First, it is, to our knowledge, the first community-based study assessing factors associated with the risk of COVID-19 hospitalization among SOTRs at the national level. Access to data from the SNDS database allowed us to conduct an exhaustive cohort study, limiting selection bias. Indeed, we found estimates of the number of living kidney, liver, heart, and lung transplant recipients in our study population similar to those described in the 2019 medical and scientific report of the national agency that registers all transplants (Agence de Biomédecine).^36,37^ Second, our study spans a significant period that covers all COVID-19 epidemic waves and virus variants that could be described. Third, our study shows rates of immunosuppressant use consistent with results reported by a French study on kidney transplant patients in 2018 and those reported worldwide.^8,10,38,39,40,41^ Fourth, the medical algorithms used in the study to identify comorbidities include associated diagnoses that make them all the more effective as this population is systematically hospitalized.

Our study also has some limitations. Data on the functional level of the grafts and the results of biological and clinical examinations are not available in the SNDS database. Therefore, residual confounding may remain due to these unmeasured variables in our analysis. However, such confounding is unlikely to have changed our results as many factors were adjusted for in our analysis, and our results are consistent with what has been found in clinical practice.^24,29,30,33^ The estimated proportions of drugs reimbursed monthly in the 6 months before the date of entry into the cohort were similar, potentially suggesting good adherence to treatment. The failure to show associations between the different factors and the risk of COVID-19 hospitalization in lung transplant patients may be related to a lack of power. We considered hospitalization as an indicator of COVID-19 severity. We could not assess intensive care unit admission because data were poorly documented, and we could not assess death because we had too few events. Vaccination status was not considered in the primary analysis. However, the sensitivity analysis of the vaccination periods did not reveal any significant differences from the main results. In addition, Semenzato et al^2,3^ reported an increased risk of severe COVID-19 in SOTRs during the first wave in 2020 and a residual risk in the period after vaccination. Belatacept, a hospital-based immunosuppressant, was not included in our analyses because its reimbursement data are not provided in the SNDS. Given that these patients are very close to the hospital system and, therefore, more likely to be hospitalized and that this drug is often administered with mycophenolic acid and steroids without calcineurin inhibitors, we believe that we have covered these patients in our cohort through reimbursement for care and medications.

## Conclusions

In this cohort study, mycophenolic acid and steroids were associated with increased risk of hospitalization for COVID-19 among SOTRs, as were advanced age and most comorbidities. Heart transplant recipients treated with sirolimus were more at risk of COVID-19 hospitalization compared with those taking other immunosuppressive drugs. Tacrolimus in liver transplant patients was associated with a decreased risk of COVID-19 hospitalization compared with other immunosuppressive drugs. Health care professionals should consider these results in treating SOTRs with SARS-CoV-2 infection by reducing doses or modifying medications in some cases. These findings could also help health authorities make appropriate decisions for this population during future epidemics. Nevertheless, further studies would be advisable to consolidate these findings.

## Notes

Supplement 1. **eTable 1.** Transplantations and Immunosuppressive Drugs Algorithms **eTable 2.** Chronic Disease Identification Algorithms **eTable 3.** Baseline Characteristics of SOTRs According to the Transplanted Organ **eTable 4.** Baseline Characteristics of KTRs According to Immunosuppressive Drugs Groups **eTable 5.** Association Between KTRs Characteristics and Risk of Hospitalization for Covid-19 in Univariate Analysis **eTable 6.** Baseline Characteristics of LTRs According to Immunosuppressive Drugs Groups **eTable 7.** Association Between LTRs Characteristics and Risk of Hospitalization for Covid-19 in Univariate Analysis **eTable 8.** Association Between Immunosuppressive Treatment Regimens and Risk of Hospitalization for Covid-19 in a Multivariate Analysis Among LTRs (Change in Reference Class) **eTable 9.** Baseline Characteristics of HTRs According to Immunosuppressive Drugs Groups **eTable 10.** Association Between HTRs Characteristics and Risk of Hospitalization for Covid-19 in Univariate Analysis **eTable 11.** Baseline Characteristics of Lung Transplant Recipients According to Immunosuppressive Drugs Groups **eTable 12.** Association Between Lung Transplant Recipients Characteristics and Risk of Hospitalization for Covid-19 in Univariate Analysis **eTable 13.** Factors Associated With Hospitalization for Covid-19 in the Multivariable Model (Sensitivity Analysis: Exclusion of Patients With More Than One Transplant) **eTable 14.** Factors Associated With Hospitalization for Covid-19 in the Multivariable Model (Sensitivity Analysis of the Largest Epidemic Wave November 2021-February 2022, Before the Largest Epidemic Wave, and After the Largest Epidemic Wave) **eTable 15.** Comparison of Risk Factors Associated With Hospitalization for Covid-19 During the Period Before the Start of Vaccination (15/02/2020-31/12/2020) and the Period After the Start of Vaccination (01/04/2021*-31/07/2022) in the Multivariable Model (Sensitivity Analysis) **eTable 16.** Comparison of Risk Factors Associated With Hospitalization for Covid-19 According to the Time After Transplant in the Multivariable Model (Sensitivity Analysis) **eTable 17.** Association Between Immunosuppressive Treatment Regimens and Risk of Hospitalization for Covid-19 in Multivariate Analysis Among SOTRs (Exploratory Analysis) **eFigure 1.** Kinetics of Hospitalizations for Covid-19 Among SOTRs **eFigure 2.** Immunosuppressive Drugs Use According to the Time Since Transplant in KTRs **eFigure 3.** Immunosuppressive Drugs Use According to the Time Since Transplant in LTRs **eFigure 4.** Immunosuppressive Drugs Use According to the Time Since Transplant in HTRs **eFigure 5.** Immunosuppressive Drugs Use According to the Time Since Transplant in Lung Transplant Recipients **eFigure 6.** Description of Treatment Regimens in SOTRs (Whole Population N = 60456)

Supplement 2.
Data Sharing Statement
