# Supplementary material for: COVID-19 Hospitalization in Solid Organ Transplant Recipients on Immunosuppressive Therapy
Source: JAMA Netw Open. 2023 Nov 7;6(11):e2342006. doi: 10.1001/jamanetworkopen.2023.42006 (PMC10630896; doi:10.1001/jamanetworkopen.2023.42006)
Supplement: Supplement 1. — eTable 1. Transplantations and Immunosuppressive Drugs Algorithms eTable 2. Chronic Disease Identification Algorithms eTable 3. Baseline Characteristics of SOTRs According to the Transplanted Organ eTable 4. Baseline Characteristics of KTRs According to Immunosuppressive Drugs Groups eTable 5. Association Between KTRs Characteristics and Risk of Hospitalization for Covid-19 in Univariate Analysis eTable 6. Baseline Characteristics of LTRs According to Immunosuppressive Drugs Groups eTable 7. Association Between LTRs Characteristics and Risk of Hospitalization for Covid-19 in Univariate Analysis eTable 8. Association Between Immunosuppressive Treatment Regimens and Risk of Hospitalization for Covid-19 in a Multivariate Analysis Among LTRs (Change in Reference Class) eTable 9. Baseline Characteristics of HTRs According to Immunosuppressive Drugs Groups eTable 10. Association Between HTRs Characteristics and Risk of Hospitalization for Covid-19 in Univariate Analysis eTable 11. Baseline Characteristics of Lung Transplant Recipients According to Immunosuppressive Drugs Groups eTable 12. Association Between Lung Transplant Recipients Characteristics and Risk of Hospitalization for Covid-19 in Univariate Analysis eTable 13. Factors Associated With Hospitalization for Covid-19 in the Multivariable Model (Sensitivity Analysis: Exclusion of Patients With More Than One Transplant) eTable 14. Factors Associated With Hospitalization for Covid-19 in the Multivariable Model (Sensitivity Analysis of the Largest Epidemic Wave November 2021-February 2022, Before the Largest Epidemic Wave, and After the Largest Epidemic Wave) eTable 15. Comparison of Risk Factors Associated With Hospitalization for Covid-19 During the Period Before the Start of Vaccination (15/02/2020-31/12/2020) and the Period After the Start of Vaccination (01/04/2021*-31/07/2022) in the Multivariable Model (Sensitivity Analysis) eTable 16. Comparison of Risk Factors Associated With Hospitalization for Covid-19 Acc [file jamanetwopen-e2342006-s001.pdf]

Supplementary Online Content

Kolla E, Weill A, Zaidan M, et al. COVID-19 hospitalization in solid organ transplant recipients on immunosuppressive therapy. *JAMA Netw Open*. 2023;6(11):e2342006.
doi:10.1001/jamanetworkopen.2023.42006

eTable 1. Transplantations and Immunosuppressive Drugs Algorithms

eTable 2. Chronic Disease Identification Algorithms

eTable 3. Baseline Characteristics of SOTRs According to the Transplanted Organ

eTable 4. Baseline Characteristics of KTRs According to Immunosuppressive Drugs Groups

eTable 5. Association Between KTRs Characteristics and Risk of Hospitalization for Covid-19 in Univariate Analysis

eTable 6. Baseline Characteristics of LTRs According to Immunosuppressive Drugs Groups

eTable 7. Association Between LTRs Characteristics and Risk of Hospitalization for Covid-19 in Univariate Analysis

eTable 8. Association Between Immunosuppressive Treatment Regimens and Risk of Hospitalization for Covid-19 in a Multivariate Analysis Among LTRs (Change in Reference Class)

eTable 9. Baseline Characteristics of HTRs According to Immunosuppressive Drugs Groups

eTable 10. Association Between HTRs Characteristics and Risk of Hospitalization for Covid-19 in Univariate Analysis

eTable 11. Baseline Characteristics of Lung Transplant Recipients According to Immunosuppressive Drugs Groups

eTable 12. Association Between Lung Transplant Recipients Characteristics and Risk of Hospitalization for Covid-19 in Univariate Analysis

eTable 13. Factors Associated With Hospitalization for Covid-19 in the Multivariable Model (Sensitivity Analysis: Exclusion of Patients With More Than One Transplant)

eTable 14. Factors Associated With Hospitalization for Covid-19 in the Multivariable Model (Sensitivity Analysis of the Largest Epidemic Wave November 2021-February 2022, Before the Largest Epidemic Wave, and After the Largest Epidemic Wave)

eTable 15. Comparison of Risk Factors Associated With Hospitalization for Covid-19 During the Period Before the Start of Vaccination (15/02/2020-31/12/2020) and the Period After the Start of Vaccination (01/04/2021*-31/07/2022) in the Multivariable Model (Sensitivity Analysis)

eTable 16. Comparison of Risk Factors Associated With Hospitalization for Covid-19 According to the Time After Transplant in the Multivariable Model (Sensitivity Analysis)

eTable 17. Association Between Immunosuppressive Treatment Regimens and Risk of Hospitalization for Covid-19 in Multivariate Analysis Among SOTRs (Exploratory Analysis)

eFigure 1. Kinetics of Hospitalizations for Covid-19 Among SOTRs

eFigure 2. Immunosuppressive Drugs Use According to the Time Since Transplant in KTRs

eFigure 3. Immunosuppressive Drugs Use According to the Time Since Transplant in LTRs

eFigure 4. Immunosuppressive Drugs Use According to the Time Since Transplant in HTRs

eFigure 5. Immunosuppressive Drugs Use According to the Time Since Transplant in Lung Transplant Recipients

eFigure 6. Description of Treatment Regimens in SOTRs (Whole Population N=60456)

This supplementary material has been provided by the authors to give readers additional information about their work.

eTable 1. Transplantations and immunosuppressive drugs algorithms

	**Identification algorithms**
Kidney transplantation	Hospitalization for kidney transplantation (ICD-10 code Z940) or<br>kidney and pancreas transplantation by laparotomy in 2019 (CCAM code JAEA003 and HNEA002) or<br>Diagnosis related groups code 27C06Z, 27C061, 27C062, 27C063, 27C064, 24M39Z, 11M171, 11M172, 11M173, 11M174
Heart transplantation	Hospitalization for heart transplantation (ICD-10 code Z941) or<br>Heart transplantation CCAM code (DZEA001 to DZEA004) or<br>Diagnosis related groups code : 27C051, 27C052, 27C053, 27C054, 05M191, 05M192, 05M193, 05M194, 05K15J, 05K151, 05K152, 05K153, 05K154
Liver transplantation	Hospitalization for liver transplantation (ICD-10 code Z944) or<br>Liver transplantation CCAM code (HLEA001, HLEA002) or<br>Diagnosis related groups code 27C021, 27C022, 27C023, 27C024, 07M121, 07M122, 07M123, 07M124
Lung transplantation	Hospitalization for lung transplantation (ICD-10 code Z942) or<br>Lung transplantation CCAM code (GFEA001 to GFEA007) or<br>Diagnosis related groups code 27C041, 27C042, 27C043, 27C044, 04M211, 04M212, 04M213, 04M214
Tacrolimus (ATC code L04AD02)	CIP Code 3400949501007, 3400930223864, 3400930001264, 3400930001226, 3400930001196, 3400922102566, 3400930223840, 3400922101965, 3400922098852, 3400930223826, 3400930223802, 3400930009291, 3400930223789, 3400949500918, 3400935895882, 3400935895714, 3400935875723, 3400949500765, 3400938069914, 3400938069624, 3400938069273, 3400939534626, 3400939594996, 3400939594828, 3400949501014, 3400937485616, 3400937485326, 3400937415477
Ciclosporin (ATC code L04AD01)	CIP code 3400949002672, 3400949001095, 3400949001071, 3400949002719, 3400949002689, 3400949002443, 3400949002436, 3400934633157, 3400934630026, 3400934630774, 3400934630606, 3400934630545, 3400934630484, 3400934630316, 3400934630255, 3400934630194
Azathioprine (ATC code L04AX01)	CIP code 3400949003815, 3400936414907, 3400936414327, 3400937409384, 3400935451316, 3400937920070, 3400949001620, 3400938549904, 3400949002474, 3400949002283, 3400930525005
Mycophenolic acid (ATC code L04AA06)	CIP code 3400935952936, 3400949510924, 3400949212866, 3400949004218, 3400936308718, 3400937400398, 3400936309197, 3400938500417, 3400949793921, 3400935952585, 3400935952707, 3400939416250, 3400921791679, 3400949141456, 3400949179596, 3400949003020, 3400939533155, 3400949918034, 3400939574257, 3400949244515, 3400939212616, 3400939989501, 3400938500646, 3400939574028, 3400949504480, 3400949917891, 3400930009468, 3400949212118, 3400949297153, 3400939611259, 3400949466368, 3400949456192, 3400949456482, 3400949511006, 3400949465996, 3400949504541, 3400930009512
Sirolimus (ATC code L04AA10)	CIP code 3400949304578, 3400935953018, 3400935688477, 3400936156821
Everolimus (ATC code L04AA18)	CIP code 3400936411135, 3400936411593, 3400936411883, 3400936410305, 3400936410824, 3400930136676
Steroids (prednisone, prednisolone, methyprednisolone) : AT code H02AB04, H02AB06, H02AB07	CIP code 3400930655955, 3400933189846, 3400937232425, 3400930515068, 3400932014668, 3400930975206, 3400934936708, 3400932820399, 3400934936876, 3400935740557, 3400936087651, 3400936087712, 3400936117389, 3400936087422, 3400936136830, 3400936137141, 3400935740496, 3400936137080, 3400936087590, 3400936087880, 3400936481732,3400936136779, 3400936806795, 3400936747913, 3400935740328, 3400939108209, 3400936196513, 3400936196452, 3400939109619, 3400939109329, 3400939108667, 3400939106885, 3400939107486, 3400939103464, 3400939102405, 3400939106595, 3400939108957, 3400939107196, 3400928099990, 3400928099976, 3400928099983, 3400928099969, 3400930050354, 3400938558609, 3400938559897, 3400938558838, 3400938560039, 3400938559958, 3400938559729, 3400938559439, 3400938559200, 3400938559378, 3400936665385, 3400936665095, 3400936521407, 3400936523128, 3400936522817, 3400936521865, 3400936519794, 3400936519565, 3400936518735, 3400936518506, 3 400936523708, 3400936523418, 3400936519916, 3400936521117, 3400936518384, 3400936520806, 3400936487307, 3400936522527, 3400936519275, 3400936665675, 3400936665217, 3400936757738, 3400934641251, 3400934641190, 3400934641022, 3400933283858, 3400930259054, 3400930258972

CIP Code : Presentation Identifier Code

© 2023 Kolla E et al. *JAMA Network Open.*

eTable 2. Chronic disease identification algorithms

	Identification algorithms
**Smoking**	Hospitalization or ALD with ICD-10: Z716, F17, T652, Z720 since 2006 or<br>reimbursement for ATC codes N06AX12 or N07BA since 2014 or<br>hospitalization with ICD-10: I731, J41, J42, J43, J44, T652 and reimbursement for ATC codes R03AC18, R03AC19, R03BB04, R03BB05, R03BB06, R03BB07, R03AL04, R03AL05, R03AK04 since 2014 or<br>Reimbursement of nicotine replacement therapy or smoking cessation consultation since 2006
**Alcohol use disorders**	Hospitalization or ALD with ICD-10: E244, E512, F10, G312, G621, G721, I426, K292, K70, K860, R780, T51, X45, X65, Y15, Y90, Y91, Y573, Z502, Z714, Z721 since 2006 or<br>Reimbursement for ATC codes N07BB01, N07BB03, N07BB04, N07BB05 since 2014
**Obesity**	Hospitalization with ICD-10: E66 (excluding codes E6603, E6613, E6683, E6693) since 2006 or<br>Bariatric surgery in the previous 5 years: CCAM codes HFCA001, HFCC003, HFFA001, HFFA011, HFFC004, HFFC018, HFGC900, HFKA001, HFKA002, HFKC001, HFMA009, HFMA010, HFMA011, HFMC006, HFMC007, HFMC008, HGCA009, HGCC027
**Diabetes**	Hospitalization or ALD with ICD-10: E10 to E14 during the previous 2 years or<br>3 reimbursements of a specific treatment, ATC codes A10 (excluding A10BX06) over 12 months for at least one of the previous 2 years or<br>Hospitalization with ICD-10: G59.0, G63.2, G73.0, G99.0, H28.0, H36.0, I79.2, L97, M14.2, M14.6, N08.3 in diabetic patients
**Hypertension**	At least 3 reimbursements for antihypertensive drugs in 2019
**Cardiovascular**	
Coronary heart disease	ALD with ICD-10: I20, I21 to I24 in 2019 or hospitalization with ICD-10: I20, I200-0, I21 to I24 in the previous 5 years
Peripheral artery disease	Hospitalization with ICD-10: I70.2, I73.9, I74.0, I74.3, I74.4, I74.5 in the previous 5 years or ALD with ICD-10: I70, I73, I74 in 2019
Cardiac arrhythmias and conduction disorders	Hospitalization with ICD-10: I44, I45, I47 to I49 in the previous 5 years or ALD with ICD-10: I44, I45, I47 to I49 in 2019
Heart failure	Hospitalization with ICD-10: I50, I11.0, I13.0, I13.2, I13.9, IK76.1, J81 in the previous 5 years or ALD with ICD-10: I50, I11, I13 in 2019
Valvular heart disease	Hospitalization with ICD-10: I05 to I08, I34 to I39 in the previous 5 years or ALD with ICD-10: I05 to I08, I34 to I39 in 2019
Stroke	Hospitalization with ICD-10: I60 to I64, I67 to I69 in the previous 5 years or ALD with ICD-10: I60 to I64, I67 to I69 in 2019
**Statin use**	At least 3 reimbursements for ATC codes C10AA05, C10AA04, C10AA03, C10AA07, C10AA01 in 2019
**Mucoviscidose**	Hospitalization with ICD-10: E84 in the previous 5 years or ALD with ICD-10 E84 in 2019
**Respiratory**	
Chronic respiratory diseases (excluding cystic fibrosis)	Hospitalization with ICD-10: J40 to J47, J96 (excluding J96.0 or J96.9), J98 in the previous 5 years or ALD with ICD-10: J40 to J47, J96, J98 in 2019 or at least 3 reimbursements of an ATC code R03 in 2019
Pulmonary embolism	Hospitalization with ICD-10: I26 in the previous 5 years
**Cancer**	
Female breast cancer (active)	Hospitalization with ICD-10: C50, D01 in the previous 2 years or ALD with ICD-10: C50, D01 starting in 2019 or 2018.
Colorectal cancer (active)	Hospitalization with ICD-10: C18 to C20, D01.0, D01.1, D01.2 in the previous 2 years or ALD with ICD-10: C18 to C20 starting in 2019 or 2018.
Lung cancer (active)	Hospitalization with ICD-10: C33, C34, D02.1, D02.2 in the previous 2 years or ALD with ICD-10: C33, C34 starting in 2019 or 2018.
Prostate cancer (active)	Hospitalization with ICD-10: C61, D07.5 in the previous 2 years or ALD with ICD-10: C61 starting in 2019 or 2018 or 3 reimbursement of hormone therapy in 2019 or 2018 (ATC codes G03HA01, L01CD04, L01XX11, L02AA01, L02AA04, L02AE01, L02AE02, L02AE03, L02AE04, L02AE05, L02BB01, L02BB02, L02BB03, L02BB04, L02BX02, L02BX03, V10BX01, V10XX).
Other cancers (active)	Hospitalization with codes ICD-10 beginning with "C" (excluding female C50, C18, C19, C20, C61, C33, C34) or D00 to D09 (excluding female D05, D01.0, D01.1, D01.2, D07.5, D02.1, D02.2) in the previous 2 years or ALD with ICD-10 codes beginning with "C" (excluding female C50, C18, C19, C20, C61, C33, C34) or D00 to D09 (excluding female D05) beginning in 2019 or 2018.
**Psychiatric diseases**	
Neurotic and mood disorders	Hospitalization with ICD-10: F30 to F34, F38 to F45, F48 in the previous two years or ALD with ICD-10: F30 to F34, F38 to F45, F48 in 2019
Psychotic disorders	Hospitalization with ICD-10: F20 to F25, F28, F29 in the previous two years or ALD with ICD-10: F20 to F25, F28, F29 in 2019
Childhood mental illness	Hospitalization with ICD-10: F80 to F84, F88 to F95, F98 in the previous two years or ALD with ICD-10: F80 to F84, F88 to F95, F98 in 2019.
Mental retardation	Hospitalization with ICD-10: F70 to F73, F78, F79 in the previous 2 years or ALD with ICD-10: F70 to F73, F78, F79 in 2019.
**Antidepressant use**	3 reimbursements of an antidepressant ATC code N06A (excluding CIP13 3400933338022), Lithium ATC code N05AN01, Dépakote® CIP13 3400934876233, 3400934876691, 340093544427 or Dépamide® ATC code N03AG02 in 2019
**Anxiolytic use**	3 reimbursements of an anxiolytic ATC code N05BA01, N05BA04, N05BA05, N05BA06, N05BA08, N05BA09, N05BA11, N05BA12, N05BA16, N05BA18, N05BA21, N05BA23, N05BB01, N05BB02, N05BC01, N05BE01, N05BX03 in 2019.
**Chronic dialysis**	45 days of haemodialysis or at least 1 day of peritoneal dialysis in 2019 or at least 1 day of haemodialysis (and less than 45 days) in 2019 and at least 45 days of haemodialysis or at least 1 day of peritoneal dialysis in 2018 without kidney transplantation or followed by kidney transplantation in 2019.

eTable 3. Baseline characteristics of SOTRs according to the transplanted organ.

Effective	Whole study population 60456	Kidney transplant[1] 41463	Liver transplant 14464	Heart transplant 5327	Lung transplant 2823
**Age mean (SD)**	56 (15.5)	56.2 (15.0)	56.6 (16.9)	55.2 (16.0)	50.3 (14.9)
**Age median (IQR)**	59 (47-67)	58 (47-67)	61 (50-68)	58 (46-67)	53 (38-63)
**Age groups**					
01-45	13725 (22.7)	9546 (23.0)	2849 (19.7)	1299 (24.4)	1025 (36.3)
46-65	28171 (46.6)	19362 (46.7)	6603 (45.7)	2465 (46.3)	1342 (47.5)
66 and up	18560 (30.7)	12555 (30.3)	5012 (34.7)	1563 (29.3)	456 (16.2)
**Age categories**					
01-17	1290 (2.1)	525 (1.3)	687 (4.7)	135 (2.5)	36 (1.3)
18-29	2664 (4.4)	1632 (3.9)	659 (4.6)	293 (5.5)	269 (9.5)
30-49	13796 (22.8)	10432 (25.2)	2169 (15.0)	1217 (22.8)	911 (32.3)
50-59	13987 (23.1)	9873 (23.8)	3059 (21.1)	1209 (22.7)	663 (23.5)
60-69	17090 (28.3)	10772 (26.0)	4959 (34.3)	1537 (28.9)	755 (26.7)
70-79	9931 (16.4)	6920 (16.7)	2625 (18.1)	787 (14.8)	178 (6.3)
80 and up	1698 (2.8)	1309 (3.2)	306 (2.1)	149 (2.8)	11 (0.4)
**Sex (Female)**	21967 (36.3)	15893 (38.3)	4723 (32.7)	1411 (26.5)	1337 (47.4)
**region**					
Île-de-France	11594 (19.2)	7944 (19.2)	2925 (20.2)	938 (17.6)	496 (17.6)
Centre-Val de Loire	2746 (4.5)	1858 (4.5)	653 (4.5)	276 (5.2)	91 (3.2)
Bourgogne-Franche-Comté	2512 (4.2)	1662 (4.0)	596 (4.1)	263 (4.9)	144 (5.1)
Normandie	3057 (5.1)	2082 (5.0)	697 (4.8)	304 (5.7)	139 (4.9)
Hauts-de-France	4601 (7.6)	3089 (7.5)	1124 (7.8)	419 (7.9)	234 (8.3)
Grand Est	5018 (8.3)	3313 (8.0)	1148 (7.9)	488 (9.2)	366 (13.0)
Pays de la Loire	3453 (5.7)	2372 (5.7)	746 (5.2)	357 (6.7)	160 (5.7)
Bretagne	3117 (5.2)	2034 (4.9)	769 (5.3)	326 (6.1)	169 (6.0)
Nouvelle-Aquitaine	5298 (8.8)	3729 (9.0)	1157 (8.0)	434 (8.1)	220 (7.8)
Occitanie	5252 (8.7)	3740 (9.0)	1351 (9.3)	378 (7.1)	206 (7.3)
Auvergne-Rhône-Alpes	7673 (12.7)	5214 (12.6)	1978 (13.7)	737 (13.8)	311 (11.0)
Provence-Alpes-Côte d’Azur	4437 (7.3)	3075 (7.4)	1027 (7.1)	295 (5.5)	252 (8.9)
Corse	199 (0.3)	145 (0.3)	45 (0.3)	10 (0.2)	10 (0.4)
DOM-TOM	1499 (2.5)	1206 (2.9)	248 (1.7)	102 (1.9)	25 (0.9)
**Social deprivation index**					
1 (least deprived)	10909 (18.0)	7375 (17.8)	2703 (18.7)	990 (18.6)	551 (19.5)

© 2023 Kolla E et al. *JAMA Network Open.*

	Whole study population	Kidney transplant[1]	Liver transplant	Heart transplant	Lung transplant
2	11554 (19.1)	7913 (19.1)	2878 (19.9)	983 (18.5)	567 (20.1)
3	11752 (19.4)	8174 (19.7)	2700 (18.7)	997 (18.7)	544 (19.3)
4	11975 (19.8)	8136 (19.6)	2876 (19.9)	1097 (20.6)	560 (19.8)
5 (most deprived)	12193 (20.2)	8314 (20.1)	2896 (20.0)	1091 (20.5)	549 (19.4)
Unknown	2073 (3.4)	1551 (3.7)	411 (2.8)	169 (3.2)	52 (1.8)
**Comorbidities**					
Alcohol	10074 (16.7)	2392 (5.8)	7714 (53.3)	554 (10.4)	276 (9.8)
Smoking	13845 (22.9)	7822 (18.9)	4181 (28.9)	1656 (31.1)	1214 (43.0)
Obesity	12211 (20.2)	7833 (18.9)	3530 (24.4)	1154 (21.7)	397 (14.1)
Hypertension	46146 (76.3)	33840 (81.6)	9057 (62.6)	4192 (78.7)	1691 (59.9)
Cardiovascular[2]	19322 (32.0)	11731 (28.3)	3538 (24.5)	4589 (86.1)	1011 (35.8)
Statin use	23444 (38.8)	17130 (41.3)	3214 (22.2)	3703 (69.5)	785 (27.8)
Diabetes	17483 (28.9)	11531 (27.8)	5020 (34.7)	1301 (24.4)	1054 (37.3)
Dialysis	240 (0.4)	0 (0.0)	104 (0.7)	113 (2.1)	34 (1.2)
Respiratory[3]	6630 (11.0)	3421 (8.3)	1603 (11.1)	694 (13.0)	-
Cancer	5511 (9.1)	3182 (7.7)	2007 (13.9)	461 (8.7)	270 (9.6)
Psychiatric	3232 (5.3)	1710 (4.1)	1075 (7.4)	422 (7.9)	281 (10.0)
Antidepressants use	3687 (6.1)	2394 (5.8)	868 (6.0)	411 (7.7)	305 (10.8)
Anxiolytics use	5649 (9.3)	3513 (8.5)	1490 (10.3)	667 (12.5)	368 (13.0)
**Age of Transplantation**					
Under 2 years	9171 (15.2)	6014 (14.5)	2200 (15.2)	627 (11.8)	557 (19.7)
2-5 years	13673 (22.6)	9094 (21.9)	3283 (22.7)	1097 (20.6)	811 (28.7)
5-10 years	16958 (28.1)	11390 (27.5)	4252 (29.4)	1480 (27.8)	877 (31.1)
10 years and up	20654 (34.2)	14965 (36.1)	4729 (32.7)	2123 (39.9)	578 (20.5)
**Immunosuppressive drugs**					
**Steroids**	36926 (61.1)	28565 (68.9)	4165 (28.8)	4222 (79.3)	2437 (86.3)
**Cumulative DDD for prednisolone**					
0	23530 (38.9)	12898 (31.1)	10299 (71.2)	1105 (20.7)	386 (13.7)
1-75	9589 (15.9)	7161 (17.3)	1690 (11.7)	872 (16.4)	555 (19.7)
76-99	8904 (14.7)	7391 (17.8)	871 (6.0)	750 (14.1)	476 (16.9)
100-150	9661 (16.0)	7520 (18.1)	855 (5.9)	1367 (25.7)	595 (21.1)
151 and up	8772 (14.5)	6493 (15.7)	749 (5.2)	1233 (23.1)	811 (28.7)
**Tacrolimus**	42354 (70.1)	28932 (69.8)	11094 (76.7)	2614 (49.1)	2438 (86.4)
**Ciclosporin**	12678 (21.0)	8915 (21.5)	1537 (10.6)	2494 (46.8)	352 (12.5)
**Mycophenolic Acid**	43971 (72.7)	31303 (75.5)	8990 (62.2)	4109 (77.1)	2035 (72.1)

© 2023 Kolla E et al. *JAMA Network Open.*

	Whole study population	Kidney transplant[1]	Liver transplant	Heart transplant	Lung transplant
**Cumulative DDD for mycophenolic acid[3]**					
0	16485 (27.3)	10160 (24.5)	5474 (37.8)	1218 (22.9)	788 (27.9)
1-65	12398 (20.5)	9457 (22.8)	2376 (16.4)	903 (17.0)	482 (17.1)
66-100	13045 (21.6)	9686 (23.4)	2790 (19.3)	862 (16.2)	445 (15.8)
101-140	8304 (13.7)	6127 (14.8)	1493 (10.3)	732 (13.7)	375 (13.3)
141 and up	10224 (16.9)	6033 (14.6)	2331 (16.1)	1612 (30.3)	733 (26.0)
**Azathioprine**	4161 (6.9)	3574 (8.6)	348 (2.4)	202 (3.8)	259 (9.2)
**Sirolimus**	1363 (2.3)	1144 (2.8)	232 (1.6)	35 (0.7)	24 (0.9)
**Everolimus**	7002 (11.6)	3280 (7.9)	2242 (15.5)	1535 (28.8)	526 (18.6)

[1]149 kidney transplant patients had mucoviscidose.
[2]Except for heart transplantation
[3]Except for mucoviscidose and lung transplantation
DDD: Define Daily Dose

© 2023 Kolla E et al. *JAMA Network Open.*

1- Kidney transplant recipients (KTRs): N = 41463

eTable 4. Baseline characteristics of KTRs according to immunosuppressive drugs groups.

	Tacrolimus		Ciclosporin		Mycophenolic Acid		Azathioprine		Sirolimus		Everolimus	
	No	Yes	No	Yes	No	Yes	No	Yes	No	Yes	No	Yes
**Effective**	**12531**	**28932**	**32548**	**8915**	**10160**	**31303**	**37889**	**3574**	**40319**	**1144**	**38183**	**3280**
**Age mean (SD)**	60.2 (13.7)	54.4 (15.1)	55.1 (15.1)	60.1 (13.9)	57.8 (15.8)	55.7 (14.6)	56.6 (14.7)	52 (16.8)	56 (15)	61.4 (12.9)	56 (15.0)	58.5 (14.4)
**Age median (IQR)**	62 (52-70)	56 (45-66)	57 (45-67)	62 (52-70)	60 (47-70)	57 (46-67)	58 (47-68)	53 (39-66)	58 (46-67)	63 (54-71)	58 (46-67)	61 (50-69)
**Age groups**												
01-45	1825 (14.6)	7721 (26.7)	8225 (25.3)	1321 (14.8)	2237 (22.0)	7309 (23.3)	8226 (21.7)	1320 (36.9)	9413 (23.3)	133 (11.6)	8951 (23.4)	595 (18.1)
46-65	5683 (45.4)	13679 (47.3)	15318 (47.1)	4044 (45.4)	4152 (40.9)	15210 (48.6)	18026 (47.6)	1336 (37.4)	18851 (46.8)	511 (44.7)	17857 (46.8)	1505 (45.9)
66 and up	5023 (40.1)	7532 (26.0)	9005 (27.7)	3550 (39.8)	3771 (37.1)	8784 (28.1)	11637 (30.7)	918 (25.7)	12055 (29.9)	500 (43.7)	11375 (29.8)	1180 (36.0)
**Age categories**												
01-17	63 (0.5)	462 (1.6)	469 (1.4)	56 (0.6)	168 (1.7)	357 (1.1)	436 (1.2)	89 (2.5)	520 (1.3)	5 (0.4)	495 (1.3)	30 (0.9)
18-29	275 (2.2)	1357 (4.7)	1435 (4.4)	197 (2.2)	345 (3.4)	1287 (4.1)	1401 (3.7)	231 (6.5)	1617 (4.0)	15 (1.3)	1543 (4.0)	89 (2.7)
30-49	2267 (18.1)	8165 (28.2)	8791 (27.0)	1641 (18.4)	2360 (23.2)	8072 (25.8)	9184 (24.2)	1248 (34.9)	10253 (25.4)	179 (15.6)	9745 (25.5)	687 (20.9)
50-59	2821 (22.5)	7052 (24.4)	7856 (24.1)	2017 (22.6)	1985 (19.5)	7888 (25.2)	9224 (24.3)	649 (18.2)	9625 (23.9)	248 (21.7)	9146 (24.0)	727 (22.2)
60-69	3628 (29.0)	7144 (24.7)	8223 (25.3)	2549 (28.6)	2673 (26.3)	8099 (25.9)	10030 (26.5)	742 (20.8)	10431 (25.9)	341 (29.8)	9802 (25.7)	970 (29.6)
70-79	2831 (22.6)	4089 (14.1)	4952 (15.2)	1968 (22.1)	2099 (20.7)	4821 (15.4)	6410 (16.9)	510 (14.3)	6615 (16.4)	305 (26.7)	6253 (16.4)	667 (20.3)
80 and up	646 (5.2)	663 (2.3)	822 (2.5)	487 (5.5)	530 (5.2)	779 (2.5)	1204 (3.2)	105 (2.9)	1258 (3.1)	51 (4.5)	1199 (3.1)	110 (3.4)
**Sex (Female)**	4454 (35.5)	11439 (39.5)	12697 (39.0)	3196 (35.8)	4502 (44.3)	11391 (36.4)	13901 (36.7)	1992 (55.7)	15522 (38.5)	371 (32.4)	14748 (38.6)	1145 (34.9)
**Region of residence**												
Île-de-France	2364 (18.9)	5580 (19.3)	6515 (20.0)	1429 (16.0)	1851 (18.2)	6093 (19.5)	7105 (18.8)	839 (23.5)	7781 (19.3)	163 (14.2)	7276 (19.1)	668 (20.4)
Northwest	2720 (21.7)	6190 (21.4)	7014 (21.5)	1896 (21.3)	1986 (19.5)	6924 (22.1)	8290 (21.9)	620 (17.3)	8612 (21.4)	298 (26)	8374 (21.9)	536 (16.3)
Northeast	2984 (23.8)	5080 (17.6)	5809 (17.8)	2255 (25.3)	2171 (21.4)	5893 (18.8)	7265 (19.2)	799 (22.4)	7771 (19.3)	293 (25.6)	7308 (19.1)	756 (23.0)
Southeast	2486 (19.8)	7763 (26.8)	8472 (26.0)	1777 (19.9)	2727 (26.8)	7522 (24.0)	9293 (24.5)	956 (26.7)	10025 (24.9)	224 (19.6)	9607 (25.2)	642 (19.6)
Southwest	1977 (15.8)	4319 (14.9)	4738 (14.6)	1558 (17.5)	1425 (14.0)	4871 (15.6)	5936 (15.7)	360 (10.1)	6130 (15.2)	166 (14.5)	5618 (14.7)	678 (20.7)
**Social deprivation index**												
1 (least deprived)	2244 (17.9)	5131 (17.7)	5898 (18.1)	1477 (16.6)	1876 (18.5)	5499 (17.6)	6660 (17.6)	715 (20.0)	7173 (17.8)	202 (17.7)	6717 (17.6)	658 (20.1)
2	2315 (18.5)	5598 (19.3)	6324 (19.4)	1589 (17.8)	2026 (19.9)	5887 (18.8)	7237 (19.1)	676 (18.9)	7685 (19.1)	228 (19.9)	7211 (18.9)	702 (21.4)
3	2402 (19.2)	5772 (20.0)	6449 (19.8)	1725 (19.3)	2013 (19.8)	6161 (19.7)	7523 (19.9)	651 (18.2)	7919 (19.6)	255 (22.3)	7584 (19.9)	590 (18.0)
4	2464 (19.7)	5672 (19.6)	6353 (19.5)	1783 (20.0)	1998 (19.7)	6138 (19.6)	7477 (19.7)	659 (18.4)	7929 (19.7)	207 (18.1)	7488 (19.6)	648 (19.8)
5 (most deprived)	2612 (20.8)	5702 (19.7)	6378 (19.6)	1936 (21.7)	1974 (19.4)	6340 (20.3)	7573 (20.0)	741 (20.7)	8081 (20.0)	233 (20.4)	7707 (20.2)	607 (18.5)

© 2023 Kolla E et al. *JAMA Network Open.*

	Tacrolimus		Ciclosporin		Mycophenolic Acid		Azathioprine		Sirolimus		Everolimus	
	**No**	**Yes**	**No**	**Yes**	**No**	**Yes**	**No**	**Yes**	**No**	**Yes**	**No**	**Yes**
**Effective**	**12531**	**28932**	**32548**	**8915**	**10160**	**31303**	**37889**	**3574**	**40319**	**1144**	**38183**	**3280**
Unknown	494 (3.9)	1057 (3.7)	1146 (3.5)	405 (4.5)	273 (2.7)	1278 (4.1)	1419 (3.7)	132 (3.7)	1532 (3.8)	19 (1.7)	1476 (3.9)	75 (2.3)
**Comorbidities**												
Alcohol	652 (5.2)	1740 (6.0)	1962 (6.0)	430 (4.8)	604 (5.9)	1788 (5.7)	2276 (6.0)	116 (3.2)	2345 (5.8)	47 (4.1)	2142 (5.6)	250 (7.6)
Smoking	2015 (16.1)	5807 (20.1)	6448 (19.8)	1374 (15.4)	1707 (16.8)	6115 (19.5)	7281 (19.2)	541 (15.1)	7663 (19.0)	159 (13.9)	7129 (18.7)	693 (21.1)
Obesity	2393 (19.1)	5440 (18.8)	6078 (18.7)	1755 (19.7)	1669 (16.4)	6164 (19.7)	7286 (19.2)	547 (15.3)	7690 (19.1)	143 (12.5)	7245 (19.0)	588 (17.9)
Hypertension	10814 (86.3)	23026 (79.6)	26072 (80.1)	7768 (87.1)	8165 (80.4)	25675 (82.0)	31221 (82.4)	2619 (73.3)	32836 (81.4)	1004 (87.8)	31055 (81.3)	2785 (84.9)
Cardiovascular[1]	3840 (30.6)	7891 (27.3)	9066 (27.9)	2665 (29.9)	3119 (30.7)	8612 (27.5)	10957 (28.9)	774 (21.7)	11354 (28.2)	377 (33.0)	10639 (27.9)	1092 (33.3)
Statin use	6253 (49.9)	10877 (37.6)	12773 (39.2)	4357 (48.9)	4093 (40.3)	13037 (41.6)	15924 (42.0)	1206 (33.7)	16442 (40.8)	688 (60.1)	15590 (40.8)	1540 (47.0)
Diabetes	3006 (24.0)	8525 (29.5)	9467 (29.1)	2064 (23.2)	2730 (26.9)	8801 (28.1)	10727 (28.3)	804 (22.5)	11227 (27.8)	304 (26.6)	10505 (27.5)	1026 (31.3)
Respiratory[2]	1083 (8.6)	2338 (8.1)	2677 (8.2)	744 (8.3)	864 (8.5)	2557 (8.2)	3178 (8.4)	243 (6.8)	3320 (8.2)	101 (8.8)	3133 (8.2)	288 (8.8)
Cancer	1291 (10.3)	1891 (6.5)	2434 (7.5)	748 (8.4)	1369 (13.5)	1813 (5.8)	2965 (7.8)	217 (6.1)	2988 (7.4)	194 (17.0)	2559 (6.7)	623 (19)
Psychiatric	517 (4.1)	1193 (4.1)	1326 (4.1)	384 (4.3)	446 (4.4)	1264 (4.0)	1548 (4.1)	162 (4.5)	1685 (4.2)	25 (2.2)	1587 (4.2)	123 (3.8)
Antidepressants use	782 (6.2)	1612 (5.6)	1852 (5.7)	542 (6.1)	661 (6.5)	1733 (5.5)	2160 (5.7)	234 (6.5)	2317 (5.7)	77 (6.7)	2214 (5.8)	180 (5.5)
Anxiolytics use	1129 (9.0)	2384 (8.2)	2750 (8.4)	763 (8.6)	954 (9.4)	2559 (8.2)	3205 (8.5)	308 (8.6)	3407 (8.5)	106 (9.3)	3231 (8.5)	282 (8.6)
**Number of consulations (6 months prior to index date)**												
0	987 (7.9)	2205 (7.6)	2582 (7.9)	610 (6.8)	726 (7.1)	2466 (7.9)	2879 (7.6)	313 (8.8)	3110 (7.7)	82 (7.2)	3000 (7.9)	192 (5.9)
1-3	4696 (37.5)	10802 (37.3)	12221 (37.5)	3277 (36.8)	3480 (34.3)	12018 (38.4)	14200 (37.5)	1298 (36.3)	15105 (37.5)	393 (34.4)	14442 (37.8)	1056 (32.2)
4-6	3637 (29.0)	7905 (27.3)	8925 (27.4)	2617 (29.4)	2913 (28.7)	8629 (27.6)	10523 (27.8)	1019 (28.5)	11181 (27.7)	361 (31.6)	10637 (27.9)	905 (27.6)
7 and up	3211 (25.6)	8020 (27.7)	8820 (27.1)	2411 (27.0)	3041 (29.9)	8190 (26.2)	10287 (27.2)	944 (26.4)	10923 (27.1)	308 (26.9)	10104 (26.5)	1127 (34.4)
**Number of hospitalizations (6 months prior to index date)**												
0	9823 (78.4)	21855 (75.5)	24735 (76.0)	6943 (77.9)	7546 (74.3)	24132 (77.1)	28850 (76.1)	2828 (79.1)	30789 (76.4)	889 (77.7)	29361 (76.9)	2317 (70.6)
1-3	2441 (19.5)	6195 (21.4)	6874 (21.1)	1762 (19.8)	2208 (21.7)	6428 (20.5)	7974 (21.0)	662 (18.5)	8406 (20.8)	230 (20.1)	7768 (20.3)	868 (26.5)
4-6	190 (1.5)	685 (2.4)	725 (2.2)	150 (1.7)	263 (2.6)	612 (2.0)	810 (2.1)	65 (1.8)	852 (2.1)	23 (2.0)	797 (2.1)	78 (2.4)
7 and up	77 (0.6)	197 (0.7)	214 (0.7)	60 (0.7)	143 (1.4)	131 (0.4)	255 (0.7)	19 (0.5)	272 (0.7)	2 (0.2)	257 (0.7)	17 (0.5)
**Age of Transplantation**												
Under 2 years	918 (7.3)	5096 (17.6)	5246 (16.1)	768 (8.6)	914 (9.0)	5100 (16.3)	5708 (15.1)	306 (8.6)	5988 (14.9)	26 (2.3)	5331 (14.0)	683 (20.8)
2-5 years	1888 (15.1)	7206 (24.9)	7789 (23.9)	1305 (14.6)	2060 (20.3)	7034 (22.5)	8362 (22.1)	732 (20.5)	9031 (22.4)	63 (5.5)	8161 (21.4)	933 (28.4)
5-10 years	3059 (24.4)	8331 (28.8)	9265 (28.5)	2125 (23.8)	2640 (26.0)	8750 (28.0)	10403 (27.5)	987 (27.6)	11135 (27.6)	255 (22.3)	10562 (27.7)	828 (25.2)
10 years and up	6666 (53.2)	8299 (28.7)	10248 (31.5)	4717 (52.9)	4546 (44.7)	10419 (33.3)	13416 (35.4)	1549 (43.3)	14165 (35.1)	800 (69.9)	14129 (37.0)	836 (25.5)

[1]Except for heart transplantation
[2]Except for mucoviscidosis and lung transplantation

© 2023 Kolla E et al. *JAMA Network Open.*

eTable 5. Association between KTRs characteristics and risk of hospitalization for Covid-19 in univariate analysis.

Effective	Not hospitalized<br>36184	Hospitalized for Covid-19<br>5279	Crude Odds Ratios OR [IC95%]
**Age groups**			
01-45	8621 (23.8)	925 (17.5)	1
46-65	16805 (46.4)	2557 (48.4)	1.42 [1.31-1.54]
66 and up	10758 (29.7)	1797 (34)	1.56 [1.43-1.69]
**Sex (Female)**	13811 (38.2)	2082 (39.4)	1.05 [0.99-1.12]
**Social deprivation index**			
1 (least deprived)	6380 (17.6)	995 (18.8)	1
2	6997 (19.3)	916 (17.4)	0.84 [0.76-0.92]
3	7259 (20.1)	915 (17.3)	0.81 [0.73-0.89]
4	7170 (19.8)	966 (18.3)	0.86 [0.79-0.95]
5 (most deprived)	7098 (19.6)	1216 (23)	1.10 [1.00-1.20]
**Comorbidities**			
Alcohol	2079 (5.7)	313 (5.9)	1.03 [0.91-1.17]
Smoking	6847 (18.9)	975 (18.5)	0.97 [0.90-1.05]
Obesity	6481 (17.9)	1352 (25.6)	1.58 [1.48-1.69]
Hypertension	29304 (81.0)	4536 (85.9)	1.43 [1.32-1.56]
Cardiovascular[1]	9823 (27.1)	1908 (36.1)	1.52 [1.43-1.61]
Statin use	14773 (40.8)	2357 (44.6)	1.17 [1.10-1.24]
Diabetes	9440 (26.1)	2091 (39.6)	1.86 [1.75-1.97]
Respiratory[2]	2824 (7.8)	597 (11.3)	1.51 [1.37-1.65]
Cancer	2808 (7.8)	374 (7.1)	0.91 [0.81-1.01]
Psychiatric	1456 (4.0)	254 (4.8)	1.21 [1.05-1.38]
Antidepressants use	2053 (5.7)	341 (6.5)	1.15 [1.02-1.29]
Anxiolytics use	3022 (8.4)	491 (9.3)	1.13 [1.02-1.24]
**Number of consulations (6 months prior to index date)**			
0	2795 (7.7)	397 (7.5)	1
1-3	13699 (37.9)	1799 (34.1)	0.92 [0.82-1.04]
4-6	10140 (28.0)	1402 (26.6)	0.97 [0.86-1.10]
7 and up	9550 (26.4)	1681 (31.8)	1.24 [1.10-1.39]
**Number of hospitalizations (6 months prior to index date)**			
0	28018 (77.4)	3660 (69.3)	1
1-3	7224 (20.0)	1412 (26.7)	1.50 [1.40-1.60]
4-6	707 (2.0)	168 (3.2)	1.82 [1.53-2.16]
7 and up	235 (0.6)	39 (0.7)	1.27 [0.90-1.79]
**Age of Transplantation**			
Under 2 years	5085 (14.1)	929 (17.6)	1
2-5 years	7810 (21.6)	1284 (24.3)	0.90 [0.82-0.98]
6-10 years	9927 (27.4)	1463 (27.7)	0.80 [0.73-0.88]
10 years and up	13362 (36.9)	1603 (30.4)	0.65 [0.60-0.71]
**Immunosuppressive drugs**			
Steroids	24378 (67.4)	4187 (79.3)	1.86 [1.73-1.99]
Tacrolimus	25218 (69.7)	3714 (70.4)	1.03 [0.97-1.10]
Ciclosporin	7804 (21.6)	1111 (21.0)	0.97 [0.90-1.04]

© 2023 Kolla E et al. *JAMA Network Open.*

Effective	Not hospitalized 36184	Hospitalized for Covid-19 5279	Crude Odds Ratios OR [IC95%]
Mycophenolic Acid	27049 (74.8)	4254 (80.6)	1.40 [1.30-1.51]
Azathioprine	3231 (8.9)	343 (6.5)	0.71 [0.63-0.80]
Sirolimus	1035 (2.9)	109 (2.1)	0.72 [0.59-0.87]
Everolimus	2887 (8.0)	393 (7.4)	0.93 [0.83-1.04]
**Treatment regimen**			
Tacrolimus-Mycophenolic Acid-Steroids	12324 (34.1)	2326 (44.1)	1
Tacrolimus-Mycophenolic Acid	6277 (17.3)	595 (11.3)	0.50 [0.46-0.55]
Ciclosporin-Mycophenolic Acid-Steroids	2973 (8.2)	566 (10.7)	1.01 [0.91-1.12]
Ciclosporin-Mycophenolic Acid	2127 (5.9)	245 (4.6)	0.61 [0.53-0.70]
Tacrolimus only	693 (1.9)	46 (0.9)	0.35 [0.26-0.48]
Tacrolimus-Steroids	1927 (5.3)	271 (5.1)	0.75 [0.65-0.85]
Tacrolimus-Sirolimus/Everolimus-Steroids	1012 (2.8)	144 (2.7)	0.75 [0.63-0.90]
Tacrolimus-Azathioprine-Steroids	1184 (3.3)	135 (2.6)	0.60 [0.50-0.73]
Ciclosporin-Steroids	1020 (2.8)	109 (2.1)	0.57 [0.46-0.69]
Mycophenolic Acid-Steroids	952 (2.6)	201 (3.8)	1.12 [0.95-1.31]
Sirolimus/Everolimus-Mycophenolic Acid	440 (1.2)	30 (0.6)	0.36 [0.25-0.52]
Sirolimus/Everolimus-Mycophenolic Acid-Steroids	750 (2.1)	115 (2.2)	0.81 [0.66-0.99]
Others	4505 (12.5)	496 (9.4)	0.58 [0.53-0.65]
**Treatment regimen classes**			
CNI + Antimétabolites + Steroids	17305 (47.8)	3150 (59.7)	1
CNI + mTORi + Steroids	1174 (3.2)	168 (3.2)	0.78 [0.66-0.92]
Antimetabolite + mTORi + Steroids	796 (2.2)	118 (2.2)	0.81 [0.66-0.99]
CNI + Antimetabolite + mTORi	110 (0.3)	7 (0.1)	0.35 [0.16-0.75]
CNI + Antimétabolites	9286 (25.7)	900 (17.0)	0.53 [0.49-0.57]
CNI + mTORi	427 (1.2)	41 (0.8)	0.52 [0.38-0.72]
CNI + Steroids	2952 (8.2)	380 (7.2)	0.70 [0.63-0.79]
Antimetabolite + Steroids	1273 (3.5)	241 (4.6)	1.04 [0.90-1.20]
Antimetabolite + mTORi	463 (1.3)	34 (0.6)	0.40 [0.28-0.57]
mTORi + Steroids	414 (1.1)	58 (1.1)	0.77 [0.58-1.01]
CNI	1116 (3.1)	68 (1.3)	0.33 [0.26-0.42]
Antimetabolite	333 (0.9)	39 (0.7)	0.64 [0.46-0.89]
mTORi	71 (0.2)	3 (0.1)	0.23 [0.07-0.73]
Others	464 (1.3)	72 (1.4)	0.85 [0.66-1.09]
**Immunosuppression intensity (including steroids)**			
Triple regimen	19849 (54.9)	3515 (66.6)	1
Double regimen	14815 (40.9)	1654 (31.3)	0.63 [0.59-0.67]
Single regimen	1520 (4.2)	110 (2.1)	0.40 [0.33-0.49]

CNI: Calcineurin Inhibitors / mTORi: mammalian target of rapamycin inhibitors.
[1]Except for heart transplantation
[2]Except for mucoviscidosis and lung transplantation

© 2023 Kolla E et al. *JAMA Network Open.*

2- Liver transplant recipients (LTRs): N = 14464

eTable 6. Baseline characteristics of LTRs according to immunosuppressive drugs groups.

	Tacrolimus		Ciclosporin		Mycophenolic Acid		Azathioprine		Sirolimus		Everolimus	
	No	Yes	No	Yes	No	Yes	No	Yes	No	Yes	No	Yes
**Effective**	**3370**	**11094**	**12927**	**1537**	**5474**	**8990**	**14116**	**348**	**14232**	**232**	**12222**	**2242**
**Age mean (SD)**	60.1 (16.8)	55.5 (16.7)	56.7 (16.4)	55.7 (20.7)	54.2 (20.2)	58 (14.3)	56.9 (16.7)	42.9 (19.0)	56.6 (16.8)	59.1 (18.2)	55.8 (17.5)	61.1 (11.6)
**Age median (IQR)**	64 (55-71)	60 (49-67)	61 (50-68)	62 (48-70)	61 (44-69)	61 (52-68)	61 (51-68)	42 (30-58)	61 (50-68)	65 (54-71)	60 (48-68)	63 (56-69)
**Age groups**												
01-45	476 (14.1)	2373 (21.4)	2489 (19.3)	360 (23.4)	1457 (26.6)	1392 (15.5)	2657 (18.8)	192 (55.2)	2813 (19.8)	36 (15.5)	2646 (21.6)	203 (9.1)
46-65	1356 (40.2)	5247 (47.3)	6037 (46.7)	566 (36.8)	2032 (37.1)	4571 (50.8)	6501 (46.1)	102 (29.3)	6515 (45.8)	88 (37.9)	5472 (44.8)	1131 (50.4)
66 and up	1538 (45.6)	3474 (31.3)	4401 (34.0)	611 (39.8)	1985 (36.3)	3027 (33.7)	4958 (35.1)	54 (15.5)	4904 (34.5)	108 (46.6)	4104 (33.6)	908 (40.5)
**Age categories**												
01-17	161 (4.8)	526 (4.7)	535 (4.1)	152 (9.9)	489 (8.9)	198 (2.2)	647 (4.6)	40 (11.5)	673 (4.7)	14 (6.0)	675 (5.5)	12 (0.5)
18-29	121 (3.6)	538 (4.8)	566 (4.4)	93 (6.1)	351 (6.4)	308 (3.4)	614 (4.3)	45 (12.9)	650 (4.6)	9 (3.9)	612 (5.0)	47 (2.1)
30-49	304 (9.0)	1865 (16.8)	2005 (15.5)	164 (10.7)	831 (15.2)	1338 (14.9)	2037 (14.4)	132 (37.9)	2146 (15.1)	23 (9.9)	1938 (15.9)	231 (10.3)
50-59	572 (17.0)	2487 (22.4)	2799 (21.7)	260 (16.9)	907 (16.6)	2152 (23.9)	3010 (21.3)	49 (14.1)	3021 (21.2)	38 (16.4)	2572 (21.0)	487 (21.7)
60-69	1193 (35.4)	3766 (33.9)	4522 (35.0)	437 (28.4)	1631 (29.8)	3328 (37.0)	4903 (34.7)	56 (16.1)	4890 (34.4)	69 (29.7)	3969 (32.5)	990 (44.2)
70-79	880 (26.1)	1745 (15.7)	2273 (17.6)	352 (22.9)	1098 (20.1)	1527 (17.0)	2602 (18.4)	23 (6.6)	2552 (17.9)	73 (31.5)	2176 (17.8)	449 (20.0)
80 and up	139 (4.1)	167 (1.5)	227 (1.8)	79 (5.1)	167 (3.1)	139 (1.5)	303 (2.1)	3 (0.9)	300 (2.1)	6 (2.6)	280 (2.3)	26 (1.2)
**Sex (Female)**	998 (29.6)	3725 (33.6)	4202 (32.5)	521 (33.9)	2134 (39.0)	2589 (28.8)	4529 (32.1)	194 (55.7)	4642 (32.6)	81 (34.9)	4164 (34.1)	559 (24.9)
**Region of residence**												
Île-de-France	550 (16.3)	2375 (21.4)	2628 (20.3)	297 (19.3)	1077 (19.7)	1848 (20.6)	2844 (20.1)	81 (23.3)	2881 (20.2)	44 (19.0)	2638 (21.6)	287 (12.8)
Northwest	702 (20.8)	2273 (20.5)	2577 (19.9)	398 (25.9)	1067 (19.5)	1908 (21.2)	2928 (20.7)	47 (13.5)	2954 (20.8)	21 (9.1)	2514 (20.6)	461 (20.6)
Northeast	975 (28.9)	1893 (17.1)	2578 (19.9)	290 (18.9)	1062 (19.4)	1806 (20.1)	2790 (19.8)	78 (22.4)	2760 (19.4)	108 (46.6)	2231 (18.3)	637 (28.4)
Southeast	793 (23.5)	3055 (27.5)	3507 (27.1)	341 (22.2)	1591 (29.1)	2257 (25.1)	3748 (26.6)	100 (28.7)	3809 (26.8)	39 (16.8)	3313 (27.1)	535 (23.9)
Southwest	350 (10.4)	1498 (13.5)	1637 (12.7)	211 (13.7)	677 (12.4)	1171 (13.0)	1806 (12.8)	42 (12.1)	1828 (12.8)	20 (8.6)	1526 (12.5)	322 (14.4)
**Social deprivation index**												
1 (least deprived)	558 (16.6)	2145 (19.3)	2423 (18.7)	280 (18.2)	1046 (19.1)	1657 (18.4)	2630 (18.6)	73 (21.0)	2671 (18.8)	32 (13.8)	2346 (19.2)	357 (15.9)
2	691 (20.5)	2187 (19.7)	2572 (19.9)	306 (19.9)	1041 (19.0)	1837 (20.4)	2797 (19.8)	81 (23.3)	2833 (19.9)	45 (19.4)	2389 (19.5)	489 (21.8)
3	609 (18.1)	2091 (18.8)	2443 (18.9)	257 (16.7)	1053 (19.2)	1647 (18.3)	2646 (18.7)	54 (15.5)	2654 (18.6)	46 (19.8)	2284 (18.7)	416 (18.6)
4	702 (20.8)	2174 (19.6)	2535 (19.6)	341 (22.2)	1069 (19.5)	1807 (20.1)	2817 (20.0)	59 (17.0)	2830 (19.9)	46 (19.8)	2388 (19.5)	488 (21.8)
5 (most deprived)	716 (21.2)	2180 (19.7)	2587 (20.0)	309 (20.1)	1131 (20.7)	1765 (19.6)	2831 (20.1)	65 (18.7)	2841 (20.0)	55 (23.7)	2451 (20.1)	445 (19.8)
Unknown	94 (2.8)	317 (2.9)	367 (2.8)	44 (2.9)	134 (2.4)	277 (3.1)	395 (2.8)	16 (4.6)	403 (2.8)	8 (3.4)	364 (3.0)	47 (2.1)
**Comorbidities**												

© 2023 Kolla E et al. *JAMA Network Open.*

	**Tacrolimus**		**Ciclosporin**		**Mycophenolic Acid**		**Azathioprine**		**Sirolimus**		**Everolimus**	
	**No**	**Yes**	**No**	**Yes**	**No**	**Yes**	**No**	**Yes**	**No**	**Yes**	**No**	**Yes**
**Effective**	**3370**	**11094**	**12927**	**1537**	**5474**	**8990**	**14116**	**348**	**14232**	**232**	**12222**	**2242**
Alcohol	1786 (53)	5928 (53.4)	7089 (54.8)	625 (40.7)	2360 (43.1)	5354 (59.6)	7649 (54.2)	65 (18.7)	7637 (53.7)	77 (33.2)	6209 (50.8)	1505 (67.1)
Smoking	914 (27.1)	3267 (29.4)	3819 (29.5)	362 (23.6)	1307 (23.9)	2874 (32)	4126 (29.2)	55 (15.8)	4135 (29.1)	46 (19.8)	3339 (27.3)	842 (37.6)
Obesity	814 (24.2)	2716 (24.5)	3202 (24.8)	328 (21.3)	1130 (20.6)	2400 (26.7)	3485 (24.7)	45 (12.9)	3493 (24.5)	37 (15.9)	2934 (24.0)	596 (26.6)
Hypertension	2447 (72.6)	6610 (59.6)	8023 (62.1)	1034 (67.3)	3168 (57.9)	5889 (65.5)	8891 (63.0)	166 (47.7)	8878 (62.4)	179 (77.2)	7434 (60.8)	1623 (72.4)
Cardiovascular[1]	969 (28.8)	2569 (23.2)	3138 (24.3)	400 (26.0)	1268 (23.2)	2270 (25.3)	3484 (24.7)	54 (15.5)	3467 (24.4)	71 (30.6)	2875 (23.5)	663 (29.6)
Statin use	1076 (31.9)	2138 (19.3)	2854 (22.1)	360 (23.4)	1144 (20.9)	2070 (23.0)	3163 (22.4)	51 (14.7)	3113 (21.9)	101 (43.5)	2454 (20.1)	760 (33.9)
Diabetes	1200 (35.6)	3820 (34.4)	4554 (35.2)	466 (30.3)	1675 (30.6)	3345 (37.2)	4930 (34.9)	90 (25.9)	4927 (34.6)	93 (40.1)	4134 (33.8)	886 (39.5)
Dialysis	31 (0.9)	73 (0.7)	92 (0.7)	12 (0.8)	63 (1.2)	41 (0.5)	102 (0.7)	2 (0.6)	101 (0.7)	3 (1.3)	87 (0.7)	17 (0.8)
Respiratory[2]	351 (10.4)	1252 (11.3)	1445 (11.2)	158 (10.3)	564 (10.3)	1039 (11.6)	1577 (11.2)	26 (7.5)	1584 (11.1)	19 (8.2)	1332 (10.9)	271 (12.1)
Cancer	461 (13.7)	1546 (13.9)	1862 (14.4)	145 (9.4)	714 (13.0)	1293 (14.4)	2000 (14.2)	7 (2.0)	1980 (13.9)	27 (11.6)	1347 (11)	660 (29.4)
Psychiatric	216 (6.4)	859 (7.7)	976 (7.6)	99 (6.4)	386 (7.1)	689 (7.7)	1046 (7.4)	29 (8.3)	1065 (7.5)	10 (4.3)	902 (7.4)	173 (7.7)
Antidepressants use	232 (6.9)	636 (5.7)	755 (5.8)	113 (7.4)	348 (6.4)	520 (5.8)	850 (6.0)	18 (5.2)	856 (6.0)	12 (5.2)	729 (6.0)	139 (6.2)
Anxiolytics use	393 (11.7)	1097 (9.9)	1314 (10.2)	176 (11.5)	570 (10.4)	920 (10.2)	1464 (10.4)	26 (7.5)	1472 (10.3)	18 (7.8)	1222 (10.0)	268 (12.0)
**Number of consulations (6 months prior to index date)**												
0	253 (7.5)	982 (8.9)	1084 (8.4)	151 (9.8)	482 (8.8)	753 (8.4)	1201 (8.5)	34 (9.8)	1219 (8.6)	16 (6.9)	1142 (9.3)	93 (4.1)
1-3	1223 (36.3)	4282 (38.6)	4911 (38.0)	594 (38.6)	2062 (37.7)	3443 (38.3)	5387 (38.2)	118 (33.9)	5424 (38.1)	81 (34.9)	4786 (39.2)	719 (32.1)
4-6	912 (27.1)	3003 (27.1)	3522 (27.2)	393 (25.6)	1507 (27.5)	2408 (26.8)	3819 (27.1)	96 (27.6)	3838 (27.0)	77 (33.2)	3282 (26.9)	633 (28.2)
7 and up	982 (29.1)	2827 (25.5)	3410 (26.4)	399 (26.0)	1423 (26.0)	2386 (26.5)	3709 (26.3)	100 (28.7)	3751 (26.4)	58 (25.0)	3012 (24.6)	797 (35.5)
**Number of hospitalizations (6 months prior to index date)**												
0	2666 (79.1)	8336 (75.1)	9778 (75.6)	1224 (79.6)	4281 (78.2)	6721 (74.8)	10726 (76.0)	276 (79.3)	10827 (76.1)	175 (75.4)	9418 (77.1)	1584 (70.7)
1-3	633 (18.8)	2448 (22.1)	2796 (21.6)	285 (18.5)	1043 (19.1)	2038 (22.7)	3017 (21.4)	64 (18.4)	3030 (21.3)	51 (22.0)	2495 (20.4)	586 (26.1)
4-6	50 (1.5)	252 (2.3)	285 (2.2)	17 (1.1)	103 (1.9)	199 (2.2)	295 (2.1)	7 (2.0)	299 (2.1)	3 (1.3)	238 (1.9)	64 (2.9)
7 and up	21 (0.6)	58 (0.5)	68 (0.5)	11 (0.7)	47 (0.9)	32 (0.4)	78 (0.6)	1 (0.3)	76 (0.5)	3 (1.3)	71 (0.6)	8 (0.4)
**Age of Transplantation**												
Under 2 years	233 (6.9)	1967 (17.7)	2069 (16.0)	131 (8.5)	491 (9.0)	1709 (19.0)	2150 (15.2)	50 (14.4)	2190 (15.4)	10 (4.3)	1761 (14.4)	439 (19.6)
2-5 years	515 (15.3)	2768 (25.0)	3101 (24.0)	182 (11.8)	1015 (18.5)	2268 (25.2)	3196 (22.6)	87 (25.0)	3266 (22.9)	17 (7.3)	2669 (21.8)	614 (27.4)
6-10 years	1084 (32.2)	3168 (28.6)	3790 (29.3)	462 (30.1)	1635 (29.9)	2617 (29.1)	4156 (29.4)	96 (27.6)	4170 (29.2)	82 (35.3)	3573 (29.2)	679 (30.3)
10 years and up	1538 (45.6)	3191 (28.8)	3967 (30.7)	762 (49.6)	2333 (42.6)	2396 (26.7)	4614 (32.7)	115 (33.0)	4606 (32.4)	123 (53.0)	4219 (34.5)	510 (22.7)

[1]Except for heart transplantation
[2]Except for mucoviscidosis and lung transplantation

© 2023 Kolla E et al. *JAMA Network Open.*

eTable 7. Association between LTRs characteristics and risk of hospitalization for Covid-19 in univariate analysis.

Effective	Not hospitalized 13539	Hospitalized for Covid-19 925	Crude Odds Ratios OR [IC95%]
**Age groups**			
01-45	2719 (20.1)	130 (14.1)	1
46-65	6198 (45.8)	405 (43.8)	1.37 [1.12-1.67]
66 and up	4622 (34.1)	390 (42.2)	1.76 [1.44-2.16]
**Sex (Female)**	4436 (32.8)	287 (31.0)	0.92 [0.80-1.07]
**Social deprivation index**			
1 (least deprived)	2521 (18.6)	182 (19.7)	1
2	2696 (19.9)	182 (19.7)	0.94 [0.76-1.16]
3	2529 (18.7)	171 (18.5)	0.94 [0.75-1.16]
4	2704 (20.0)	172 (18.6)	0.88 [0.71-1.09]
5 (most deprived)	2715 (20.1)	181 (19.6)	0.92 [0.75-1.14]
**Comorbidities**			
Alcohol	7258 (53.6)	456 (49.3)	0.84 [0.74-0.96]
Smoking	3936 (29.1)	245 (26.5)	0.88 [0.76-1.02]
Obesity	3272 (24.2)	258 (27.9)	1.21 [1.05-1.41]
Hypertension	8383 (61.9)	674 (72.9)	1.65 [1.42-1.92]
Cardiovascular[1]	3201 (23.6)	337 (36.4)	1.85 [1.61-2.13]
Statin use	2928 (21.6)	286 (30.9)	1.62 [1.40-1.88]
Diabetes	4547 (33.6)	473 (51.1)	2.07 [1.81-2.37]
Dialysis	83 (0.6)	21 (2.3)	3.77 [2.32-6.11]
Respiratory[2]	1475 (10.9)	128 (13.8)	1.31 [1.08-1.60]
Cancer	1896 (14.0)	111 (12.0)	0.84 [0.68-1.03]
Psychiatric	1003 (7.4)	72 (7.8)	1.05 [0.82-1.35]
Antidepressants use	795 (5.9)	73 (7.9)	1.37 [1.07-1.76]
Anxiolytics use	1391 (10.3)	99 (10.7)	1.05 [0.84-1.30]
**Number of consulations (6 months prior to index date)**			
0	1170 (8.6)	65 (7.0)	1
1-3	5191 (38.3)	314 (33.9)	1.09 [0.83-1.43]
4-6	3683 (27.2)	232 (25.1)	1.13 [0.85-1.50]
7 and up	3495 (25.8)	314 (33.9)	1.62 [1.23-2.13]
**Number of hospitalizations (6 months prior to index date)**			
0	10379 (76.7)	623 (67.4)	1
1-3	2825 (20.9)	256 (27.7)	1.51 [1.30-1.76]
4-6	269 (2.0)	33 (3.6)	2.04 [1.41-2.96]
7 and up	66 (0.5)	13 (1.4)	3.28 [1.80-5.98]
**Age of Transplantation**			
Under 2 year	2050 (15.1)	150 (16.2)	1
2-5 years	3074 (22.7)	209 (22.6)	0.92 [0.74-1.15]
5-10 years	3989 (29.5)	263 (28.4)	0.90 [0.73-1.10]
10 years and up	4426 (32.7)	303 (32.8)	0.93 [0.76-1.14]
**Immunosuppressive drugs**			
Steroids	3793 (28.0)	372 (40.2)	1.73 [1.51-1.98]

© 2023 Kolla E et al. *JAMA Network Open.*

Effective	Not hospitalized 13539	Hospitalized for Covid-19 925	Crude Odds Ratios OR [IC95%]
Tacrolimus	10418 (76.9)	676 (73.1)	0.81 [0.70-0.95]
Ciclosporin	1438 (10.6)	99 (10.7)	1.01 [0.81-1.25]
Mycophenolic Acid	8321 (61.5)	669 (72.3)	1.64 [1.41-1.90]
Azathioprine	328 (2.4)	20 (2.2)	0.89 [0.56-1.40]
Sirolimus	209 (1.5)	23 (2.5)	1.63 [1.05-2.51]
Everolimus	2113 (15.6)	129 (13.9)	0.88 [0.72-1.06]
**Treatment regimen**			
Tacrolimus-Mycophenolic Acid	4172 (30.8)	268 (29.0)	1
Tacrolimus-Mycophenolic Acid-Steroids	1816 (13.4)	193 (20.9)	1.65 [1.36-2.01]
Ciclosporin-Mycophenolic Acid-Steroids	190 (1.4)	38 (4.1)	3.11 [2.15-4.50]
Ciclosporin-Mycophenolic Acid	506 (3.7)	24 (2.6)	0.73 [0.48-1.13]
Tacrolimus only	2441 (18.0)	86 (9.3)	0.54 [0.42-0.70]
Tacrolimus-Steroids	735 (5.4)	54 (5.8)	1.14 [0.84-1.54]
Tacrolimus-Sirolimus/Everolimus-Steroids	217 (1.6)	16 (1.7)	1.14 [0.68-1.93]
Tacrolimus-Azathioprine-Steroids	121 (0.9)	7 (0.8)	1.15 [0.41-1.94]
Ciclosporin-Steroids	139 (1.0)	6 (0.6)	0.67 [0.29-1.53]
Mycophenolic Acid-Steroids	89 (0.7)	14 (1.5)	2.44 [1.37-4.36]
Sirolimus/Everolimus-Mycophenolic Acid	636 (4.7)	46 (5.0)	1.12 [0.81-1.55]
Sirolimus/Everolimus-Mycophenolic Acid-Steroids	115 (0.8)	16 (1.7)	2.16 [1.26-3.70]
Others	2362 (17.4)	157 (17)	1.03 [0.84-1.26]
**Treatment regimen classes**			
CNI + Antimétabolites	4803 (35.5)	300 (32.4)	1
CNI + Antimétabolites + Steroids	2173 (16.0)	239 (25.8)	1.76 [1.47-2.10]
CNI + mTORi + Steroids	229 (1.7)	19 (2.1)	1.32 [0.82-2.15]
Antimetabolite + mTORi + Steroids	126 (0.9)	16 (1.7)	2.03 [1.19-3.46]
CNI + Antimetabolite + mTORi	238 (1.8)	12 (1.3)	0.80 [0.44-1.45]
CNI + mTORi	430 (3.2)	22 (2.4)	0.81 [0.52-1.27]
CNI + Steroids	877 (6.5)	60 (6.5)	1.09 [0.82-1.45]
Antimetabolite + Steroids	102 (0.8)	16 (1.7)	2.51 [1.46-4.31]
Antimetabolite + mTORi	641 (4.7)	46 (5.0)	1.14 [0.83-1.58]
mTORi + Steroids	110 (0.8)	10 (1.1)	1.45 [0.75-2.81]
CNI	2903 (21.4)	110 (11.9)	0.60 [0.48-075]
Antimetabolite	361 (2.7)	48 (5.2)	2.12 [1.54-2.94]
mTORi	370 (2.7)	15 (1.6)	0.64 [0.38-1.10]
Others	176 (1.3)	12 (1.3)	1.09 [0.60-1.98]
**Immunosuppression intensity (including steroids)**			
Double regimen	6963 (51.4)	454 (49.1)	1
Triple regimen	2942 (21.7)	298 (32.2)	1.55 [1.33-1.80]
Single regimen	3634 (26.8)	173 (18.7)	0.73 [0.61-0.87]

CNI: Calcineurin Inhibitors / mTORi: mammalian target of rapamycin inhibitors.
[1]Except for heart transplantation
[2]Except for mucoviscidosis and lung transplantation

© 2023 Kolla E et al. *JAMA Network Open.*

eTable 8. Association between immunosuppressive treatment regimens and risk of hospitalization for Covid-19 in a multivariate analysis among LTRs (change in reference class)

	Adjusted Odds Ratios aOR [IC95%]
**Treatment regimen[1]**	
Tacrolimus-Mycophenolic Acid	1
Tacrolimus-Mycophenolic Acid-Steroids	1.51 [1.23-1.86]
Ciclosporin-Mycophenolic Acid-Steroids	2.62 [1.77-3.86]
Ciclosporin-Mycophenolic Acid	0.73 [0.47-1.13]
Tacrolimus only	0.52 [0.40-0.68]
Tacrolimus-Steroids	1.03 [0.75-1.41]
Tacrolimus-Sirolimus/Everolimus-Steroids	1.12 [0.65-1.93]
Tacrolimus-Azathioprine-Steroids	0.83 [0.37-1.83]
Ciclosporin-Steroids	0.54 [0.23-1.27]
Mycophenolic Acid-Steroids	1.71 [0.94-3.11]
Sirolimus/Everolimus-Mycophenolic Acid	1.03 [0.73-1.44]
Sirolimus/Everolimus-Mycophenolic Acid-Steroids	1.71 [0.98-2.98]
Others	0.90 [0.73-1.11]
**Treatment regimen classes[2]**	
CNI + Antimétabolites	1
CNI + Antimétabolites + Steroids	1.60 [1.32-1.93]
CNI + mTORi + Steroids	1.29 [0.78-2.13]
Antimetabolite + m+ Steroids	1.61 [0.93-2.78]
CNI + Antimetabolite + mTORi	0.84 [0.45-1.53]
CNI + mTORi	0.75 [0.48-1.19]
CNI + Steroids	0.97 [0.72-1.31]
Antimetabolite + Steroids	1.85 [1.06-3.23]
Antimetabolite + mTORi	1.04 [0.74-1.45]
mTORi + Steroids	1.07 [0.54-2.10]
CNI	0.58 [0.46-0.73]
Antimetabolite	1.60 [1.13-2.25]
mTORi	0.52 [0.30-0.91]
Others	1.02 [0.55-1.89]
**Immunosuppression intensity (including steroids)[3]**	
Double regimen	1
Single regimen	0.68 [0.57-0.82]
Triple regimen	1.49 [1.27-1.76]

CNI: Calcineurin Inhibitors / mTORi: mammalian target of rapamycin inhibitors

[1]Model 1: Adjusted for age, sex, social deprivation index, each of the comorbidities, number of consultations, number of hospitalizations, age of transplantation, and region of residence.

[2]Model 2: Adjusted for age, sex, social deprivation index, each of the comorbidities, number of consultations, number of hospitalizations, age of transplantation, and region of residence.

[3]Model 3: Adjusted for age, sex, social deprivation index, each of the comorbidities, number of consultations, number of hospitalizations, age of transplantation, and region of residence.

© 2023 Kolla E et al. *JAMA Network Open.*

3- Heart transplant recipients (HTRs): N = 5327

eTable 9. Baseline characteristics of HTRs according to immunosuppressive drugs groups.

	Tacrolimus		Ciclosporin		Mycophenolic Acid		Azathioprine		Sirolimus		Everolimus	
	**No**	**Yes**	**No**	**Yes**	**No**	**Yes**	**No**	**Yes**	**No**	**Yes**	**No**	**Yes**
**Effective**	**2713**	**2614**	**2833**	**2494**	**1218**	**4109**	**5125**	**202**	**5292**	**35**	**3792**	**1535**
**Age mean (SD)**	58.9 (14.9)	51.3 (16.2)	52.5 (16.2)	58.3 (15.2)	57.7 (16.6)	54.5 (15.8)	55.3 (15.9)	53.4 (18.9)	55.2 (16.0)	59.6 (15.3)	55 (16.4)	55.6 (15.0)
**Age median (IQR)**	62 (51-69)	54 (41-64)	55 (42-65)	61 (50-69)	61 (48-70)	58 (45-66)	58 (46-67)	56 (37-69)	58 (46-67)	61 (53-70)	58 (46-67)	58 (47-67)
**Age groups**												
01-45	466 (17.2)	833 (31.9)	841 (29.7)	458 (18.4)	266 (21.8)	1033 (25.1)	1222 (23.8)	77 (38.1)	1294 (24.5)	5 (14.3)	941 (24.8)	358 (23.3)
46-65	1211 (44.6)	1254 (48.0)	1343 (47.4)	1122 (45.0)	480 (39.4)	1985 (48.3)	2406 (46.9)	59 (29.2)	2449 (46.3)	16 (45.7)	1745 (46.0)	720 (46.9)
66 and up	1036 (38.2)	527 (20.2)	649 (22.9)	914 (36.6)	472 (38.8)	1091 (26.6)	1497 (29.2)	66 (32.7)	1549 (29.3)	14 (40.0)	1106 (29.2)	457 (29.8)
**Age categories**												
01-17	34 (1.3)	101 (3.9)	98 (3.5)	37 (1.5)	25 (2.1)	110 (2.7)	130 (2.5)	5 (2.5)	135 (2.6)	0 (0.0)	113 (3.0)	22 (1.4)
18-29	102 (3.8)	191 (7.3)	196 (6.9)	97 (3.9)	64 (5.3)	229 (5.6)	273 (5.3)	20 (9.9)	291 (5.5)	2 (5.7)	206 (5.4)	87 (5.7)
30-49	482 (17.8)	735 (28.1)	748 (26.4)	469 (18.8)	248 (20.4)	969 (23.6)	1157 (22.6)	60 (29.7)	1211 (22.9)	6 (17.1)	864 (22.8)	353 (23.0)
50-59	570 (21.0)	639 (24.4)	669 (23.6)	540 (21.7)	230 (18.9)	979 (23.8)	1184 (23.1)	25 (12.4)	1203 (22.7)	6 (17.1)	858 (22.6)	351 (22.9)
60-69	861 (31.7)	676 (25.9)	768 (27.1)	769 (30.8)	334 (27.4)	1203 (29.3)	1494 (29.2)	43 (21.3)	1527 (28.9)	10 (28.6)	1063 (28.0)	474 (30.9)
70-79	534 (19.7)	253 (9.7)	316 (11.2)	471 (18.9)	249 (20.4)	538 (13.1)	754 (14.7)	33 (16.3)	778 (14.7)	9 (25.7)	565 (14.9)	222 (14.5)
80 and up	130 (4.8)	19 (0.7)	38 (1.3)	111 (4.5)	68 (5.6)	81 (2.0)	133 (2.6)	16 (7.9)	147 (2.8)	2 (5.7)	123 (3.2)	26 (1.7)
**Sex (Female)**	505 (18.6)	906 (34.7)	940 (33.2)	471 (18.9)	383 (31.4)	1028 (25.0)	1321 (25.8)	90 (44.6)	1405 (26.5)	6 (17.1)	1078 (28.4)	333 (21.7)
**Region of residence**												
Île-de-France	574 (21.2)	364 (13.9)	410 (14.5)	528 (21.2)	188 (15.4)	750 (18.3)	893 (17.4)	45 (22.3)	936 (17.7)	2 (5.7)	673 (17.7)	265 (17.3)
Northwest	639 (23.6)	654 (25.0)	678 (23.9)	615 (24.7)	240 (19.7)	1053 (25.6)	1264 (24.7)	29 (14.4)	1289 (24.4)	4 (11.4)	960 (25.3)	333 (21.7)
Northeast	644 (23.7)	526 (20.1)	654 (23.1)	516 (20.7)	315 (25.9)	855 (20.8)	1116 (21.8)	54 (26.7)	1154 (21.8)	16 (45.7)	725 (19.1)	445 (29.0)
Southeast	549 (20.2)	707 (27.0)	715 (25.2)	541 (21.7)	340 (27.9)	916 (22.3)	1197 (23.4)	59 (29.2)	1249 (23.6)	7 (20.0)	941 (24.8)	315 (20.5)
Southwest	307 (11.3)	363 (13.9)	376 (13.3)	294 (11.8)	135 (11.1)	535 (13.0)	655 (12.8)	15 (7.4)	664 (12.5)	6 (17.1)	493 (13.0)	177 (11.5)
**Social deprivation index**												
1 (least deprived)	522 (19.2)	468 (17.9)	510 (18.0)	480 (19.2)	225 (18.5)	765 (18.6)	951 (18.6)	39 (19.3)	987 (18.7)	3 (8.6)	709 (18.7)	281 (18.3)
2	496 (18.3)	487 (18.6)	531 (18.7)	452 (18.1)	237 (19.5)	746 (18.2)	946 (18.5)	37 (18.3)	977 (18.5)	6 (17.1)	687 (18.1)	296 (19.3)
3	515 (19.0)	482 (18.4)	523 (18.5)	474 (19.0)	224 (18.4)	773 (18.8)	960 (18.7)	37 (18.3)	992 (18.7)	5 (14.3)	685 (18.1)	312 (20.3)
4	547 (20.2)	550 (21.0)	599 (21.1)	498 (20.0)	238 (19.5)	859 (20.9)	1059 (20.7)	38 (18.8)	1084 (20.5)	13 (37.1)	782 (20.6)	315 (20.5)
5 (most deprived)	535 (19.7)	556 (21.3)	596 (21.0)	495 (19.8)	262 (21.5)	829 (20.2)	1043 (20.4)	48 (23.8)	1085 (20.5)	6 (17.1)	798 (21.0)	293 (19.1)
Unknown	98 (3.6)	71 (2.7)	74 (2.6)	95 (3.8)	32 (2.6)	137 (3.3)	166 (3.2)	3 (1.5)	167 (3.2)	2 (5.7)	131 (3.5)	38 (2.5)
**Comorbidities**												

© 2023 Kolla E et al. *JAMA Network Open.*

	Tacrolimus		Ciclosporin		Mycophenolic Acid		Azathioprine		Sirolimus		Everolimus	
	**No**	**Yes**	**No**	**Yes**	**No**	**Yes**	**No**	**Yes**	**No**	**Yes**	**No**	**Yes**
**Effective**	**2713**	**2614**	**2833**	**2494**	**1218**	**4109**	**5125**	**202**	**5292**	**35**	**3792**	**1535**
Alcohol	267 (9.8)	287 (11.0)	307 (10.8)	247 (9.9)	107 (8.8)	447 (10.9)	541 (10.6)	13 (6.4)	550 (10.4)	4 (11.4)	398 (10.5)	156 (10.2)
Smoking	782 (28.8)	874 (33.4)	940 (33.2)	716 (28.7)	325 (26.7)	1331 (32.4)	1612 (31.5)	44 (21.8)	1646 (31.1)	10 (28.6)	1122 (29.6)	534 (34.8)
Obesity	605 (22.3)	549 (21.0)	614 (21.7)	540 (21.7)	253 (20.8)	901 (21.9)	1120 (21.9)	34 (16.8)	1145 (21.6)	9 (25.7)	811 (21.4)	343 (22.3)
Hypertension	2297 (84.7)	1895 (72.5)	2093 (73.9)	2099 (84.2)	975 (80.0)	3217 (78.3)	4048 (79.0)	144 (71.3)	4158 (78.6)	34 (97.1)	2911 (76.8)	1281 (83.5)
Cardiovascular[1]	2387 (88.0)	2202 (84.2)	2392 (84.4)	2197 (88.1)	1016 (83.4)	3573 (87.0)	4437 (86.6)	152 (75.2)	4567 (86.3)	22 (62.9)	3211 (84.7)	1378 (89.8)
Statin use	1949 (71.8)	1754 (67.1)	1887 (66.6)	1816 (72.8)	787 (64.6)	2916 (71.0)	3579 (69.8)	124 (61.4)	3683 (69.6)	20 (57.1)	2598 (68.5)	1105 (72.0)
Diabetes	592 (21.8)	709 (27.1)	779 (27.5)	522 (20.9)	314 (25.8)	987 (24.0)	1242 (24.2)	59 (29.2)	1290 (24.4)	11 (31.4)	921 (24.3)	380 (24.8)
Dialysis	59 (2.2)	54 (2.1)	54 (1.9)	59 (2.4)	44 (3.6)	69 (1.7)	108 (2.1)	5 (2.5)	113 (2.1)	0 (0.0)	83 (2.2)	30 (2.0)
Respiratory[2]	323 (11.9)	371 (14.2)	400 (14.1)	294 (11.8)	153 (12.6)	541 (13.2)	666 (13.0)	28 (13.9)	690 (13.0)	4 (11.4)	513 (13.5)	181 (11.8)
Cancer	269 (9.9)	192 (7.3)	228 (8.0)	233 (9.3)	175 (14.4)	286 (7.0)	444 (8.7)	17 (8.4)	455 (8.6)	6 (17.1)	297 (7.8)	164 (10.7)
Psychiatric	179 (6.6)	243 (9.3)	246 (8.7)	176 (7.1)	92 (7.6)	330 (8.0)	404 (7.9)	18 (8.9)	420 (7.9)	2 (5.7)	312 (8.2)	110 (7.2)
Antidepressants use	219 (8.1)	192 (7.3)	203 (7.2)	208 (8.3)	100 (8.2)	311 (7.6)	399 (7.8)	12 (5.9)	407 (7.7)	4 (11.4)	302 (8.0)	109 (7.1)
Anxiolytics use	368 (13.6)	299 (11.4)	335 (11.8)	332 (13.3)	156 (12.8)	511 (12.4)	642 (12.5)	25 (12.4)	661 (12.5)	6 (17.1)	492 (13.0)	175 (11.4)
**Number of consulations (6 months prior to index date)**												
0	314 (11.6)	231 (8.8)	252 (8.9)	293 (11.7)	109 (8.9)	436 (10.6)	527 (10.3)	18 (8.9)	544 (10.3)	1 (2.9)	391 (10.3)	154 (10.0)
1-3	1119 (41.2)	1118 (42.8)	1220 (43.1)	1017 (40.8)	470 (38.6)	1767 (43.0)	2151 (42.0)	86 (42.6)	2222 (42.0)	15 (42.9)	1613 (42.5)	624 (40.7)
4-6	687 (25.3)	729 (27.9)	773 (27.3)	643 (25.8)	337 (27.7)	1079 (26.3)	1369 (26.7)	47 (23.3)	1407 (26.6)	9 (25.7)	998 (26.3)	418 (27.2)
7 and up	593 (21.9)	536 (20.5)	588 (20.8)	541 (21.7)	302 (24.8)	827 (20.1)	1078 (21.0)	51 (25.2)	1119 (21.1)	10 (28.6)	790 (20.8)	339 (22.1)
**Number of hospitalizations (6 months prior to index date)**												
0	1942 (71.6)	1807 (69.1)	1980 (69.9)	1769 (70.9)	833 (68.4)	2916 (71.0)	3610 (70.4)	139 (68.8)	3723 (70.4)	26 (74.3)	2683 (70.8)	1066 (69.4)
1-3	702 (25.9)	726 (27.8)	767 (27.1)	661 (26.5)	332 (27.3)	1096 (26.7)	1373 (26.8)	55 (27.2)	1420 (26.8)	8 (22.9)	997 (26.3)	431 (28.1)
4-6	46 (1.7)	60 (2.3)	63 (2.2)	43 (1.7)	33 (2.7)	73 (1.8)	101 (2.0)	5 (2.5)	106 (2.0)	0 (0.0)	80 (2.1)	26 (1.7)
7 and up	23 (0.8)	21 (0.8)	23 (0.8)	21 (0.8)	20 (1.6)	24 (0.6)	41 (0.8)	3 (1.5)	43 (0.8)	1 (2.9)	32 (0.8)	12 (0.8)
**Age of Transplantation**												
Under 2 year	243 (9.0)	384 (14.7)	366 (12.9)	261 (10.5)	67 (5.5)	560 (13.6)	615 (12.0)	12 (5.9)	625 (11.8)	2 (5.7)	473 (12.5)	154 (10.0)
2-5 years	466 (17.2)	631 (24.1)	660 (23.3)	437 (17.5)	191 (15.7)	906 (22.0)	1070 (20.9)	27 (13.4)	1092 (20.6)	5 (14.3)	759 (20.0)	338 (22.0)
5-10 years	673 (24.8)	807 (30.7)	860 (30.4)	620 (24.9)	317 (26.0)	1163 (28.3)	1429 (27.9)	51 (25.2)	1473 (27.8)	7 (20.0)	1027 (27.1)	453 (29.5)
10 years and up	1331 (49.1)	792 (30.3)	947 (33.4)	1176 (47.2)	643 (52.8)	1480 (36.0)	2011 (39.2)	112 (55.4)	2102 (39.7)	21 (60.0)	1533 (40.4)	590 (38.4)

[1]Except for heart transplantation
[2]Except for mucoviscidose and lung transplantation

© 2023 Kolla E et al. *JAMA Network Open.*

eTable 10. Association between HTRs characteristics and risk of hospitalization for Covid-19 in univariate analysis.

	Not hospitalized	Hospitalized for Covid-19	Crude Odds Ratios OR [IC95%]
**Effective**	**4640**	**687**	
**Age groups**			
01-45	1147 (24.7)	152 (22.1)	1
46-65	2148 (46.3)	317 (46.1)	1.11 [0.91-1.37]
66 and up	1345 (29.0)	218 (31.7)	1.22 [0.98-1.53]
**Sex (Female)**	1229 (26.5)	182 (26.5)	1.00 [0.83-1.20]
**Social deprivation index**			
1 (least deprived)	859 (18.5)	131 (19.1)	1
2	848 (18.3)	135 (19.7)	1.04 [0.81-1.35]
3	880 (19.0)	117 (17.0)	0.87 [0.67-1.14]
4	976 (21.0)	121 (17.6)	0.81 [0.62-1.06]
5 (most deprived)	942 (20.3)	149 (21.7)	1.04 [0.81-1.33]
**Comorbidities**			
Alcohol	480 (10.3)	74 (10.8)	1.05 [0.81-1.36]
Smoking	1431 (30.8)	225 (32.8)	1.09 [0.92-1.30]
Obesity	963 (20.8)	191 (27.8)	1.47 [1.23-1.76]
Hypertension	3631 (78.3)	561 (81.7)	1.24 [1.01-1.52]
Cardiovascular[1]	3974 (85.6)	615 (89.5)	1.43 [1.11-1.85]
Statin use	3189 (68.7)	514 (74.8)	1.35 [1.13-1.62]
Diabetes	1084 (23.4)	217 (31.6)	1.51 [1.27-1.80]
Dialysis	83 (1.8)	30 (4.4)	2.51 [1.64-3.84]
Respiratory[2]	586 (12.6)	108 (15.7)	1.29 [1.03-1.61]
Cancer	399 (8.6)	62 (9.0)	1.05 [0.80-1.40]
Psychiatric	368 (7.9)	54 (7.9)	0.99 [0.74-1.33]
Antidepressants use	357 (7.7)	54 (7.9)	1.02 [0.76-1.38]
Anxiolytics use	566 (12.2)	101 (14.7)	1.24 [0.99-1.56]
**Number of consulations (6 months prior to index date)**			
0	471 (10.2)	74 (10.8)	1
1-3	1964 (42.3)	273 (39.7)	0.88 [0.67-1.17]
4-6	1243 (26.8)	173 (25.2)	0.89 [0.66-1.19]
7 and up	962 (20.7)	167 (24.3)	1.10 [0.82-1.48]
**Number of hospitalizations (6 months prior to index date)**			
0	3318 (71.5)	431 (62.7)	1
1-3	1202 (25.9)	226 (32.9)	1.45 [1.22-1.72]
4-6	87 (1.9)	19 (2.8)	1.68 [1.01-2.79]
7 and up	33 (0.7)	11 (1.6)	2.57 [1.29-5.11]
**Age of Transplantation**			
Under 2 year	532 (11.5)	95 (13.8)	1
2-5 years	929 (20.0)	168 (24.5)	1.01 [0.77-1.33]
5-10 years	1307 (28.2)	173 (25.2)	0.74 [0.56-0.97]
10 years and up	1872 (40.3)	251 (36.5)	0.75 [0.58-0.96]
**Immunosuppressive drugs**			

© 2023 Kolla E et al. *JAMA Network Open.*

Effective	Not hospitalized<br>4640	Hospitalized for Covid-19<br>687	Crude Odds Ratios OR [IC95%]
Steroids	3634 (78.3)	588 (85.6)	1.64 [1.31-2.06]
Tacrolimus	2266 (48.8)	348 (50.7)	1.08 [0.92-1.26]
Ciclosporin	2204 (47.5)	290 (42.2)	0.81 [0.69-0.95]
Mycophenolic Acid	3566 (76.9)	543 (79.0)	1.14 [0.93-1.38]
Azathioprine	174 (3.8)	28 (4.1)	1.09 [0.73-1.64]
Sirolimus	25 (0.5)	10 (1.5)	2.73 [1.30-5.70]
Everolimus	1313 (28.3)	222 (32.3)	1.21 [1.02-1.44]
**Treatment regimen**			
Tacrolimus-Mycophenolic Acid-Steroids	1111 (23.9)	189 (27.5)	1
Tacrolimus-Mycophenolic Acid	305 (6.6)	25 (3.6)	0.48 [0.31-0.75]
Ciclosporin-Mycophenolic Acid-Steroids	1032 (22.2)	138 (20.1)	0.79 [0.62-0.99]
Ciclosporin-Mycophenolic Acid	276 (5.9)	28 (4.1)	0.60 [0.39-0.91]
Tacrolimus only	36 (0.8)	3 (0.4)	0.49 [0.15-1.61]
Tacrolimus-Steroids	147 (3.2)	17 (2.5)	0.68 [0.40-1.15]
Tacrolimus-Sirolimus/Everolimus-Steroids	198 (4.3)	43 (6.3)	1.28 [0.89-1.84]
Tacrolimus-Azathioprine-Steroids	59 (1.3)	11 (1.6)	1.10 [0.57-2.12]
Ciclosporin-Steroids	161 (3.5)	20 (2.9)	0.73 [0.45-1.19]
Mycophenolic Acid-Steroids	12 (0.3)	9 (1.3)	4.41 [1.83-10.61]
Sirolimus/Everolimus-Mycophenolic Acid	59 (1.3)	9 (1.3)	0.90 [0.44-1.84]
Sirolimus/Everolimus-Mycophenolic Acid-Steroids	113 (2.4)	32 (4.7)	1.66 [1.09-2.54]
Others	1131 (24.4)	163 (23.7)	0.85 [0.68-1.06]
**Treatment regimen classes**			
CNI + Antimétabolites + Steroids	2289 (49.3)	347 (50.5)	1
CNI + mTORi + Steroids	386 (8.3)	62 (9.0)	1.06 [0.79-1.41]
Antimetabolite + mTORi + Steroids	117 (2.5)	34 (4.9)	1.91 [1.28-2.85]
CNI + Antimetabolite + mTORi	131 (2.8)	17 (2.5)	0.85 [0.51-1.43]
CNI + Antimétabolites	622 (13.4)	58 (8.4)	0.61 [0.45-0.82]
CNI + mTORi	118 (2.5)	10 (1.5)	0.55 [0.29-1.07]
CNI + Steroids	311 (6.7)	37 (5.4)	0.78 [0.54-1.12]
Antimetabolite + Steroids	15 (0.3)	9 (1.3)	3.95 [1.71-9.11]
Antimetabolite + mTORi	59 (1.3)	9 (1.3)	1.01 [0.49-2.04]
mTORi + Steroids	17 (0.4)	2 (0.3)	0.77 [0.17-3.37]
CNI	63 (1.4)	4 (0.6)	0.41 [0.15-1.15]
Antimetabolite	2 (0.0)	0 (0.0)	-
mTORi	11 (0.2)	1 (0.1)	0.60 [0.07-4.65]
Others	499 (10.8)	97 (14.1)	1.28 [1.00-1.63]
**Immunosuppression intensity (including steroids)**			
Triple regimen	3422 (73.8)	557 (81.1)	1
Double regimen	1142 (24.6)	125 (18.2)	0.67 [0.54-0.82]
Single regimen	76 (1.6)	5 (0.7)	0.40 [0.16-1.00]

CNI: Calcineurin Inhibitors / mTORi: mammalian target of rapamycin inhibitors.
[1]Except for heart transplantation
[2]Except for mucoviscidosis and lung transplantation

© 2023 Kolla E et al. *JAMA Network Open.*

4- Lung transplant recipients: N = 2881

eTable 11. Baseline characteristics of lung transplant recipients according to immunosuppressive drugs groups.

	Tacrolimus		Ciclosporin		Mycophenolic Acid		Azathioprine		Sirolimus		Everolimus	
	No	Yes	No	Yes	No	Yes	No	Yes	No	Yes	No	Yes
**Effective**	**385**	**2438**	**2471**	**352**	**788**	**2035**	**2564**	**259**	**2799**	**24**	**2297**	**526**
**Age mean (SD)**	55 (13.5)	49.6 (15.0)	49.8 (15.0)	54.1 (13.6)	50.5 (14.8)	50.3 (15.0)	50.5 (14.9)	48.5 (15.4)	50.3 (15.0)	54.2 (11.8)	50.1 (15.1)	51.4 (14.3)
**Age median (IQR)**	57 (45-66)	53 (37-62)	53 (37-62)	56 (45-64)	53 (39-63)	53 (38-63)	54 (39-63)	49 (35-63)	53 (38-63)	53 (48-64)	53 (38-63)	55 (41-63)
**Age groups**												
01-45	99 (25.7)	926 (38)	929 (37.6)	96 (27.3)	285 (36.2)	740 (36.4)	910 (35.5)	115 (44.4)	1020 (36.4)	5 (20.8)	854 (37.2)	171 (32.5)
46-65	189 (49.1)	1153 (47.3)	1164 (47.1)	178 (50.6)	378 (48.0)	964 (47.4)	1236 (48.2)	106 (40.9)	1327 (47.4)	15 (62.5)	1069 (46.5)	273 (51.9)
66 and up	97 (25.2)	359 (14.7)	378 (15.3)	78 (22.2)	125 (15.9)	331 (16.3)	418 (16.3)	38 (14.7)	452 (16.1)	4 (16.7)	374 (16.3)	82 (15.6)
**Age categories**												
01-17	4 (1.0)	32 (1.3)	32 (1.3)	4 (1.1)	12 (1.5)	24 (1.2)	33 (1.3)	3 (1.2)	36 (1.3)	0 (0.0)	27 (1.2)	9 (1.7)
18-29	10 (2.6)	259 (10.6)	258 (10.4)	11 (3.1)	59 (7.5)	210 (10.3)	246 (9.6)	23 (8.9)	269 (9.6)	0 (0.0)	235 (10.2)	34 (6.5)
30-49	111 (28.8)	800 (32.8)	804 (32.5)	107 (30.4)	270 (34.3)	641 (31.5)	807 (31.5)	104 (40.2)	904 (32.3)	7 (29.2)	747 (32.5)	164 (31.2)
50-59	96 (24.9)	567 (23.3)	572 (23.1)	91 (25.9)	185 (23.5)	478 (23.5)	617 (24.1)	46 (17.8)	655 (23.4)	8 (33.3)	524 (22.8)	139 (26.4)
60-69	115 (29.9)	640 (26.3)	656 (26.5)	99 (28.1)	199 (25.3)	556 (27.3)	692 (27.0)	63 (24.3)	748 (26.7)	7 (29.2)	611 (26.6)	144 (27.4)
70-79	45 (11.7)	133 (5.5)	141 (5.7)	37 (10.5)	59 (7.5)	119 (5.8)	160 (6.2)	18 (6.9)	176 (6.3)	2 (8.3)	143 (6.2)	35 (6.7)
80 and up	4 (1.0)	7 (0.3)	8 (0.3)	3 (0.9)	4 (0.5)	7 (0.3)	9 (0.4)	2 (0.8)	11 (0.4)	0 (0.0)	10 (0.4)	1 (0.2)
**Sex (Female)**	182 (47.3)	1155 (47.4)	1169 (47.3)	168 (47.7)	442 (56.1)	895 (44.0)	1165 (45.4)	172 (66.4)	1323 (47.3)	14 (58.3)	1085 (47.2)	252 (47.9)
**Region of residence**												
Île-de-France	59 (15.3)	437 (17.9)	447 (18.1)	49 (13.9)	134 (17.0)	362 (17.8)	445 (17.4)	51 (19.7)	490 (17.5)	6 (25.0)	410 (17.8)	86 (16.3)
Northwest	110 (28.6)	464 (19.0)	472 (19.1)	102 (29.0)	226 (28.7)	348 (17.1)	532 (20.7)	42 (16.2)	569 (20.3)	5 (20.8)	459 (20.0)	115 (21.9)
Northeast	79 (20.5)	665 (27.3)	681 (27.6)	63 (17.9)	167 (21.2)	577 (28.4)	665 (25.9)	79 (30.5)	740 (26.4)	4 (16.7)	632 (27.5)	112 (21.3)
Southeast	50 (13.0)	615 (25.2)	617 (25.0)	48 (13.6)	186 (23.6)	479 (23.5)	590 (23.0)	75 (29.0)	656 (23.4)	9 (37.5)	537 (23.4)	128 (24.3)
Southwest	87 (22.6)	257 (10.5)	254 (10.3)	90 (25.6)	75 (9.5)	269 (13.2)	332 (12.9)	12 (4.6)	344 (12.3)	0 (0.0)	259 (11.3)	85 (16.2)
**Social deprivation index**												
1 (least deprived)	61 (15.8)	490 (20.1)	489 (19.8)	62 (17.6)	165 (20.9)	386 (19.0)	492 (19.2)	59 (22.8)	545 (19.5)	6 (25.0)	450 (19.6)	101 (19.2)
2	80 (20.8)	487 (20.0)	494 (20.0)	73 (20.7)	151 (19.2)	416 (20.4)	515 (20.1)	52 (20.1)	563 (20.1)	4 (16.7)	468 (20.4)	99 (18.8)
3	88 (22.9)	456 (18.7)	466 (18.9)	78 (22.2)	155 (19.7)	389 (19.1)	496 (19.3)	48 (18.5)	539 (19.3)	5 (20.8)	441 (19.2)	103 (19.6)
4	84 (21.8)	476 (19.5)	482 (19.5)	78 (22.2)	162 (20.6)	398 (19.6)	513 (20.0)	47 (18.1)	553 (19.8)	7 (29.2)	444 (19.3)	116 (22.1)
5 (most deprived)	64 (16.6)	485 (19.9)	495 (20.0)	54 (15.3)	149 (18.9)	400 (19.7)	498 (19.4)	51 (19.7)	548 (19.6)	1 (4.2)	450 (19.6)	99 (18.8)
Unknown	8 (2.1)	44 (1.8)	45 (1.8)	7 (2.0)	6 (0.8)	46 (2.3)	50 (2.0)	2 (0.8)	51 (1.8)	1 (4.2)	44 (1.9)	8 (1.5)
**Comorbidities**												

© 2023 Kolla E et al. *JAMA Network Open.*

	Tacrolimus		Ciclosporin		Mycophenolic Acid		Azathioprine		Sirolimus		Everolimus	
	**No**	**Yes**	**No**	**Yes**	**No**	**Yes**	**No**	**Yes**	**No**	**Yes**	**No**	**Yes**
**Effective**	**385**	**2438**	**2471**	**352**	**788**	**2035**	**2564**	**259**	**2799**	**24**	**2297**	**526**
Alcohol	46 (11.9)	230 (9.4)	235 (9.5)	41 (11.6)	75 (9.5)	201 (9.9)	256 (10.0)	20 (7.7)	272 (9.7)	4 (16.7)	230 (10.0)	46 (8.7)
Smoking	154 (40.0)	1060 (43.5)	1067 (43.2)	147 (41.8)	308 (39.1)	906 (44.5)	1115 (43.5)	99 (38.2)	1209 (43.2)	5 (20.8)	976 (42.5)	238 (45.2)
Obesity	63 (16.4)	334 (13.7)	339 (13.7)	58 (16.5)	109 (13.8)	288 (14.2)	366 (14.3)	31 (12.0)	393 (14.0)	4 (16.7)	311 (13.5)	86 (16.3)
Hypertension	287 (74.5)	1404 (57.6)	1431 (57.9)	260 (73.9)	534 (67.8)	1157 (56.9)	1534 (59.8)	157 (60.6)	1674 (59.8)	17 (70.8)	1311 (57.1)	380 (72.2)
Cardiovascular[1]	197 (51.2)	814 (33.4)	823 (33.3)	188 (53.4)	267 (33.9)	744 (36.6)	934 (36.4)	77 (29.7)	1008 (36.0)	3 (12.5)	797 (34.7)	214 (40.7)
Statin use	149 (38.7)	636 (26.1)	654 (26.5)	131 (37.2)	218 (27.7)	567 (27.9)	733 (28.6)	52 (20.1)	775 (27.7)	10 (41.7)	591 (25.7)	194 (36.9)
Diabetes	90 (23.4)	964 (39.5)	979 (39.6)	75 (21.3)	292 (37.1)	762 (37.4)	960 (37.4)	94 (36.3)	1049 (37.5)	5 (20.8)	859 (37.4)	195 (37.1)
Dialysis	11 (2.9)	23 (0.9)	24 (1.0)	10 (2.8)	18 (2.3)	16 (0.8)	28 (1.1)	6 (2.3)	33 (1.2)	1 (4.2)	25 (1.1)	9 (1.7)
Cancer	47 (12.2)	223 (9.1)	234 (9.5)	36 (10.2)	141 (17.9)	129 (6.3)	250 (9.8)	20 (7.7)	262 (9.4)	8 (33.3)	169 (7.4)	101 (19.2)
Psychiatric	34 (8.8)	247 (10.1)	244 (9.9)	37 (10.5)	94 (11.9)	187 (9.2)	251 (9.8)	30 (11.6)	276 (9.9)	5 (20.8)	232 (10.1)	49 (9.3)
Antidepressants use	39 (10.1)	266 (10.9)	269 (10.9)	36 (10.2)	92 (11.7)	213 (10.5)	276 (10.8)	29 (11.2)	301 (10.8)	4 (16.7)	235 (10.2)	70 (13.3)
Anxiolytics use	55 (14.3)	313 (12.8)	316 (12.8)	52 (14.8)	109 (13.8)	259 (12.7)	336 (13.1)	32 (12.4)	368 (13.1)	0 (0.0)	284 (12.4)	84 (16.0)
**Number of consulations (6 months prior to index date)**												
0	41 (10.6)	255 (10.5)	263 (10.6)	33 (9.4)	67 (8.5)	229 (11.3)	278 (10.8)	18 (6.9)	292 (10.4)	4 (16.7)	250 (10.9)	46 (8.7)
1-3	144 (37.4)	1070 (43.9)	1083 (43.8)	131 (37.2)	331 (42.0)	883 (43.4)	1106 (43.1)	108 (41.7)	1204 (43.0)	10 (41.7)	995 (43.3)	219 (41.6)
4-6	112 (29.1)	668 (27.4)	676 (27.4)	104 (29.5)	222 (28.2)	558 (27.4)	692 (27.0)	88 (34.0)	775 (27.7)	5 (20.8)	620 (27.0)	160 (30.4)
7 and up	88 (22.9)	445 (18.3)	449 (18.2)	84 (23.9)	168 (21.3)	365 (17.9)	488 (19.0)	45 (17.4)	528 (18.9)	5 (20.8)	432 (18.8)	101 (19.2)
**Number of hospitalizations (6 months prior to index date)**												
0	245 (63.6)	1401 (57.5)	1428 (57.8)	218 (61.9)	466 (59.1)	1180 (58.0)	1486 (58.0)	160 (61.8)	1633 (58.3)	13 (54.2)	1360 (59.2)	286 (54.4)
1-3	124 (32.2)	860 (35.3)	868 (35.1)	116 (33.0)	262 (33.2)	722 (35.5)	904 (35.3)	80 (30.9)	977 (34.9)	7 (29.2)	791 (34.4)	193 (36.7)
4-6	11 (2.9)	144 (5.9)	143 (5.8)	12 (3.4)	45 (5.7)	110 (5.4)	141 (5.5)	14 (5.4)	153 (5.5)	2 (8.3)	116 (5.1)	39 (7.4)
7 and up	5 (1.3)	33 (1.4)	32 (1.3)	6 (1.7)	15 (1.9)	23 (1.1)	33 (1.3)	5 (1.9)	36 (1.3)	2 (8.3)	30 (1.3)	8 (1.5)
**Age of Transplantation**												
Under 2 year	56 (14.5)	501 (20.2)	494 (20.0)	63 (17.9)	78 (9.9)	479 (23.5)	532 (20.7)	25 (9.7)	555 (19.8)	2 (8.3)	502 (21.9)	55 (10.5)
2-5 years	89 (23.1)	722 (29.6)	736 (29.8)	75 (21.3)	230 (29.2)	581 (28.6)	744 (29.0)	67 (25.9)	806 (28.8)	5 (20.8)	649 (28.3)	162 (30.8)
5-10 years	120 (31.2)	757 (31.1)	769 (31.1)	108 (30.7)	275 (34.9)	602 (29.6)	777 (30.3)	100 (38.6)	873 (31.2)	4 (16.7)	693 (30.2)	186 (35.0)
10 years and up	120 (31.2)	458 (18.8)	472 (19.1)	106 (30.1)	205 (26.0)	373 (18.3)	511 (19.9)	67 (25.9)	565 (20.2)	13 (54.2)	453 (19.7)	125 (23.8)

[1]Except for heart transplantation

© 2023 Kolla E et al. *JAMA Network Open.*

eTable 12. Association between lung transplant recipients characteristics and risk of hospitalization for Covid-19 in univariate analysis.

Effective	Not hospitalized 2312	Hospitalized for Covid-19 511	Crude Odds Ratios OR [IC95%]
**Age groups**			
01-45	851 (36.8)	174 (34.1)	1
46-65	1082 (46.8)	260 (50.9)	1.18 [0.95-1.45]
66 and up	379 (16.4)	77 (15.1)	0.99 [0.74-1.33]
**Sex (Female)**	1099 (47.5)	238 (46.6)	0.96 [0.79-1.17]
**Social deprivation index**			
1 (least deprived)	444 (19.2)	107 (20.9)	1
2	473 (20.5)	94 (18.4)	0.82 [0.61-1.12]
3	460 (19.9)	84 (16.4)	0.76 [0.55-1.04]
4	472 (20.4)	88 (17.2)	0.77 [0.57-1.06]
5 (most deprived)	424 (18.3)	125 (24.5)	1.22 [0.91-1.64]
**Comorbidities**			
Alcohol	224 (9.7)	52 (10.2)	1.06 [0.77-1.45]
Smoking	964 (41.7)	250 (48.9)	1.34 [1.11-1.62]
Obesity	305 (13.2)	92 (18.0)	1.44 [1.12-1.87]
Hypertension	1390 (60.1)	301 (58.9)	0.95 [0.78-1.16]
Cardiovascular[1]	811 (35.1)	200 (39.1)	1.19 [0.98-1.45]
Statin use	631 (27.3)	154 (30.1)	1.15 [0.93-1.42]
Diabetes	840 (36.3)	214 (41.9)	1.26 [1.04-1.53]
Dialysis	25 (1.1)	9 (1.8)	1.64 [0.76-3.54]
Cancer	228 (9.9)	42 (8.2)	0.82 [0.58-1.15]
Psychiatric	230 (9.9)	51 (10.0)	1.00 [0.73-1.38]
Antidepressants use	242 (10.5)	63 (12.3)	1.20 [0.90-1.62]
Anxiolytics use	283 (12.2)	85 (16.6)	1.43 [1.10-1.86]
**Number of consulations (6 months prior to index date)**			
0	255 (11.0)	41 (8.0)	1
1-3	1010 (43.7)	204 (39.9)	1.26 [0.87-1.81]
4-6	636 (27.5)	144 (28.2)	1.41 [0.97-2.05]
7 and up	411 (17.8)	122 (23.9)	1.85 [1.25-2.72]
**Number of hospitalizations (6 months prior to index date)**			
0	1371 (59.3)	275 (53.8)	1
1-3	792 (34.3)	192 (37.6)	1.21 [0.99-1.48]
4-6	121 (5.2)	34 (6.7)	1.40 [0.94-2.09]
7 and up	28 (1.2)	10 (2.0)	1.78 [0.85-3.71]
**Age of Transplantation**			
Under 2 year	434 (18.8)	123 (24.1)	1
2-5 years	647 (28.0)	164 (32.1)	0.89 [0.68-1.16]
5-10 years	741 (32.1)	136 (26.6)	0.64 [0.49-0.84]
10 years and up	490 (21.2)	88 (17.2)	0.63 [0.46-0.85]
**Immunosuppressive drugs**			
Steroids	1966 (85.0)	471 (92.2)	2.07 [1.47-2.92]
Tacrolimus	1980 (85.6)	458 (89.6)	1.45 [1.07-1.97]
Ciclosporin	307 (13.3)	45 (8.8)	0.63 [0.45-0.88]

© 2023 Kolla E et al. *JAMA Network Open.*

Effective	Not hospitalized<br>2312	Hospitalized for Covid-19<br>511	Crude Odds Ratios OR [IC95%]
Mycophenolic Acid	1640 (70.9)	395 (77.3)	1.40 [1.11-1.75]
Azathioprine	225 (9.7)	34 (6.7)	0.66 [0.45-0.96]
Sirolimus	18 (0.8)	6 (1.2)	1.51 [0.60-3.83]
Everolimus	429 (18.6)	97 (19.0)	1.03 [0.81-1.31]
**Treatment regimen**			
Tacrolimus-Mycophenolic Acid-Steroids	1071 (46.3)	303 (59.3)	1
Tacrolimus-Mycophenolic Acid	187 (8.1)	20 (3.9)	0.38 [0.23-0.61]
Ciclosporin-Mycophenolic Acid-Steroids	156 (6.7)	27 (5.3)	0.61 [0.40-0.94]
Ciclosporin-Mycophenolic Acid	28 (1.2)	4 (0.8)	0.50 [0.18-1.45]
Tacrolimus only	33 (1.4)	2 (0.4)	0.21 [0.05-0.90]
Tacrolimus-Steroids	124 (5.4)	15 (2.9)	0.43 [0.25-0.74]
Tacrolimus-Sirolimus/Everolimus-Steroids	211 (9.1)	53 (10.4)	0.89 [0.64-1.23]
Tacrolimus-Azathioprine-Steroids	138 (6.0)	23 (4.5)	0.59 [0.37-0.93]
Ciclosporin-Steroids	31 (1.3)	4 (0.8)	0.46 [0.16-1.30]
Mycophenolic Acid-Steroids	7 (0.3)	1 (0.2)	0.50 [0.06-4.12]
Sirolimus/Everolimus-Mycophenolic Acid	3 (0.1)	1 (0.2)	1.18 [0.12-11.37]
Sirolimus/Everolimus-Mycophenolic Acid-Steroids	15 (0.6)	3 (0.6)	0.71 [0.20-2.46]
Others	308 (13.3)	55 (10.8)	0.63 [0.46-0.86]
**Treatment regimen classes**			
CNI + Antimétabolites + Steroids	1413 (61.1)	358 (70.1)	1
CNI + mTORi + Steroids	234 (10.1)	57 (11.2)	0.96 [0.70-1.31]
Antimetabolite + mTORi + Steroids	17 (0.7)	3 (0.6)	0.69 [0.20-2.39]
CNI + Antimetabolite + mTORi	32 (1.4)	4 (0.8)	0.49 [0.17-1.40]
CNI + Antimétabolites	245 (10.6)	26 (5.1)	0.41 [0.27-0.63]
CNI + mTORi	20 (0.9)	5 (1.0)	0.98 [0.36-2.64]
CNI + Steroids	155 (6.7)	19 (3.7)	0.48 [0.29-0.79]
Antimetabolite + Steroids	9 (0.4)	2 (0.4)	0.87 [0.18-4.07]
Antimetabolite + mTORi	3 (0.1)	1 (0.2)	1.31 [0.13-12.68]
MTORi + Steroids	16 (0.7)	1 (0.2)	0.24 [0.03-1.86
CNI	42 (1.8)	2 (0.4)	0.18 [0.04-0.78]
Antimetabolite	2 (0.1)	1 (0.2)	1.97 [0.17-21.82]
mTORi	2 (0.1)	1 (0.2)	1.97 [0.17-21.82]
Others	122 (5.3)	31 (6.1)	1.00 [0.66-1.51]
**Immunosuppression intensity**			
Triple regimen	1818 (78.6)	453 (88.6)	1
Double regimen	448 (19.4)	54 (10.6)	0.48 [0.35-0.65]
Single regimen	46 (2.0)	4 (0.8)	0.34 [0.12-0.97]

CNI: Calcineurin Inhibitors / mTORi: mammalian target of rapamycin inhibitors.
[1]Except for heart transplantation

© 2023 Kolla E et al. *JAMA Network Open.*

eTable 13. Factors associated with hospitalization for Covid-19 in the multivariable model (sensitivity analysis: exclusion of patients with more than one transplant)

	Adjusted Odds Ratios aOR [IC95%][1]			
	**Kidney**	**Liver**	**Heart**	**Lung**
**Number of hospitalizations cases/Effective**	**4880/38422**	**646/12152**	**533/4299**	**401/2127**
**Age groups**				
01-45	1	1	1	1
46-65	1.36 [1.24-1.49]	1.34 [0.99-1.81]	0.94 [0.73-1.22]	0.95 [0.69-1.32]
66 and up	1.47 [1.32-1.62]	1.71 [1.25-2.34]	1.17 [0.87-1.57]	0.86 [0.56-1.33]
**Sex**				
Male	1	1	1	1
Female	1.04 [0.98-1.11]	0.85 [0.70-1.02]	1.09 [0.86-1.37]	0.97 [0.77-1.23]
**Social deprivation index**				
1 (least deprived)	1	1	1	1
2	1.05 [0.94-1.17]	0.91 [0.69-1.19]	1.26 [0.93-1.71]	0.90 [0.62-1.32]
3	1.11 [0.99-1.23]	1.13 [0.86-1.49]	1.11 [0.80-1.55]	0.85 [0.57-1.27]
4	1.20 [1.08-1.34]	1.00 [0.76-1.32]	0.95 [0.68-1.32]	0.86 [0.57-1.27]
5 (most deprived)	1.34 [1.21-1.49]	1.02 [0.77-1.35]	1.03 [0.75-1.43]	1.26 [0.86-1.85]
**Comorbidities**				
Alcohol	0.97 [0.83-1.13]	0.86 [0.71-1.05]	0.93 [0.67-1.30]	0.91 [0.62-1.35]
Smoking	0.83 [0.76-0.91]	0.70 [0.57-0.86]	0.95 [0.77-1.18]	1.09 [0.82-1.44]
Obesity	1.21 [1.12-1.31]	0.96 [0.79-1.16]	1.34 [1.07-1.68]	1.29 [0.94-1.77]
Hypertension	1.13 [1.03-1.24]	1.14 [0.94-1.39]	1.17 [0.90-1.51]	0.98 [0.76-1.25]
Cardiovascular[2]	1.21 [1.12-1.30]	1.25 [1.03-1.51]	1.34 [0.91-1.97]	1.09 [0.85-1.41]
Statin use	0.97 [0.91-1.04]	1.13 [0.93-1.39]	1.27 [1.01-1.59]	1.10 [0.83-1.46]
Diabetes	1.43 [1.34-1.54]	1.61 [1.34-1.92]	1.22 [0.97-1.52]	1.13 [0.89-1.44]
Dialysis	-	2.90 [1.63-5.17]	2.15 [1.29-3.57]	1.62 [0.61-4.33]
Respiratory[3]	1.29 [1.16-1.43]	1.10 [0.86-1.41]	1.10 [0.82-1.47]	1.23 [0.89-1.69]
Cancer	0.91 [0.80-1.03]	0.77 [0.60-0.99]	1.11 [0.78-1.57]	0.82 [0.52-1.28]
Psychiatric	1.21 [1.04-1.40]	1.02 [0.74-1.40]	0.96 [0.67-1.37]	1.06 [0.72-1.56]
Antidepressants use	1.09 [0.96-1.25]	1.31 [0.96-1.79]	0.88 [0.61-1.27]	1.25 [0.87-1.78]
Anxiolytics use	1.06 [0.95-1.19]	0.95 [0.72-1.25]	1.33 [1.01-1.76]	1.29 [0.93-1.79]
**Age of Transplantation**				
Under 2 year	1	1	1	1
2-5 years	1.06 [0.96-1.18]	1.17 [0.87-1.56]	1.07 [0.78-1.47]	1.12 [0.82-1.54]
5-10 years	1.00 [0.91-1.11]	1.11 [0.83-1.48]	0.86 [0.62-1.19]	0.93 [0.65-1.32]
10 years and up	0.86 [0.78-0.96]	1.17 [0.87-1.57]	0.88 [0.63-1.23]	0.83 [0.53-1.30]
**Immunosuppressive drugs**				
Steroids	1.63 [1.51-1.77]	1.48 [1.22-1.79]	1.30 [0.99-1.72]	1.64 [1.05-2.56]
Tacrolimus	1.00 [0.89-1.13]	0.87 [0.66-1.15]	0.74 [0.49-1.12]	0.81 [0.38-1.70]
Ciclosporin	1.03 [0.91-1.17]	0.73 [0.50-1.07]	0.60 [0.40-0.89]	0.63 [0.28-1.38]
Mycophenolic Acid	1.38 [1.25-1.52]	1.55 [1.28-1.87]	1.29 [0.98-1.70]	1.41 [0.96-2.07]
Azathioprine	0.91 [0.78-1.05]	1.36 [0.73-2.51]	0.98 [0.53-1.82]	0.63 [0.36-1.08]
Sirolimus	0.78 [0.62-0.99]	1.31 [0.73-2.34]	1.91 [0.62-5.88]	1.74 [0.54-5.60]
Everolimus	0.94 [0.82-1.07]	0.82 [0.62-1.09]	1.33 [1.07-1.66]	1.13 [0.78-1.63]

[1]Adjusted on region of residence, number of consultations and number of hospitalizations
[2]Except for heart transplantation
[3]Except for mucoviscidosis and lung transplantation

© 2023 Kolla E et al. *JAMA Network Open.*

eTable 14.1 Factors associated with hospitalization for Covid-19 in the multivariable model (sensitivity analysis of the largest epidemic wave November 2021-February 2022)

	Adjusted Odds Ratios aOR [IC95%][1]			
	**Kidney**	**Liver**	**Heart**	**Lung**
**Number of hospitalizations cases/Effective**	**1565/38777**	**263/13722**	**216/5021**	**171/2447**
**Age groups**				
01-45	1	1	1	1
46-65	1.19 [1.03-1.38]	0.91 [0.61-1.36]	1.22 [0.84-1.76]	1.20 [0.76-1.89]
66 and up	1.24 [1.05-1.47]	1.25 [0.82-1.93]	0.93 [0.59-1.45]	1.18 [0.66-2.09]
**Sex**				
Male	1	1	1	1
Female	1.14 [1.02-1.27]	1.02 [0.77-1.35]	0.97 [0.70-1.35]	1.02 [0.73-1.42]
**Social deprivation index**				
1 (least deprived)	1	1	1	1
2	1.07 [0.9-1.27]	1.53 [1.01-2.32]	1.87 [1.19-2.95]	1.43 [0.81-2.52]
3	1.05 [0.87-1.25]	1.41 [0.91-2.20]	1.23 [0.74-2.05]	1.50 [0.82-2.74]
4	1.12 [0.94-1.34]	1.14 [0.72-1.80]	1.07 [0.64-1.78]	1.11 [0.60-2.05]
5([most deprived)	1.15 [0.97-1.37]	1.03 [0.64-1.66]	1.34 [0.82-2.18]	1.88 [1.06-3.34]
**Comorbidities**				
Alcohol	0.83 [0.66-1.05]	0.73 [0.55-0.97]	0.98 [0.61-1.57]	0.68 [0.37-1.24]
Smoking	0.93 [0.81-1.06]	0.88 [0.65-1.19]	0.83 [0.60-1.15]	1.22 [0.82-1.82]
Obesity	1.21 [1.07-1.38]	0.89 [0.66-1.20]	1.00 [0.71-1.42]	1.14 [0.74-1.77]
Hypertension	1.23 [1.06-1.44]	1.79 [1.29-2.48]	0.92 [0.64-1.30]	0.89 [0.63-1.26]
Cardiovascular[2]	1.22 [1.08-1.37]	1.46 [1.10-1.95]	1.65 [0.98-2.79]	1.09 [0.76-1.56]
Statin use	1.00 [0.90-1.12]	1.10 [0.82-1.49]	1.24 [0.89-1.72]	0.97 [0.67-1.42]
Diabetes	1.46 [1.30-1.63]	1.48 [1.13-1.94]	1.50 [1.09-2.05]	1.24 [0.89-1.74]
Dialysis	-	2.52 [0.95-6.68]	1.54 [0.69-3.41]	1.08 [0.28-4.11]
Respiratory[3]	1.43 [1.21-1.68]	1.22 [0.85-1.76]	1.03 [0.68-1.55]	1.33 [0.86-2.04]
Cancer	0.89 [0.72-1.10]	0.73 [0.49-1.10]	0.73 [0.42-1.28]	0.70 [0.37-1.32]
Psychiatric	0.94 [0.72-1.23]	0.84 [0.50-1.40]	0.91 [0.54-1.53]	0.81 [0.46-1.42]
Antidepressants use	1.16 [0.94-1.43]	0.59 [0.31-1.09]	0.66 [0.37-1.17]	1.00 [0.60-1.67]
Anxiolytics use	1.02 [0.85-1.22]	1.34 [0.91-1.96]	1.31 [0.87-1.98]	0.97 [0.60-1.59]
**Age of Transplantation**				
Under 2 year	1	1	1	1
2-5 years	0.96 [0.82-1.13]	1.26 [0.82-1.93]	1.52 [0.94-2.46]	0.97 [0.61-1.53]
5-10 years	0.91 [0.78-1.07]	1.25 [0.82-1.91]	1.16 [0.71-1.91]	0.76 [0.46-1.27]
10 years and up	0.75 [0.63-0.88]	0.98 [0.63-1.52]	1.07 [0.65-1.76]	0.96 [0.54-1.72]
**Immunosuppressive drugs**				
Steroids	1.63 [1.43-1.86]	1.57 [1.20-2.06]	1.63 [1.05-2.53]	3.56 [1.53-8.30]
Tacrolimus	1.16 [0.94-1.42]	0.97 [0.62-1.53]	1.16 [0.61-2.21]	1.39 [0.50-3.86]
Ciclosporin	1.06 [0.85-1.33]	0.74 [0.41-1.34]	0.78 [0.42-1.47]	1.17 [0.41-3.33]
Mycophenolic Acid	1.61 [1.36-1.90]	1.87 [1.37-2.54]	1.09 [0.73-1.61]	1.34 [0.79-2.27]
Azathioprine	0.98 [0.76-1.26]	0.71 [0.26-1.99]	1.42 [0.67-2.99]	0.90 [0.43-1.88]
Sirolimus	0.90 [0.61-1.35]	0.41 [0.10-1.72]	0.99 [0.12-8.05]	1.33 [0.26-6.88]
Everolimus	0.92 [0.74-1.16]	0.74 [0.47-1.17]	1.35 [0.97-1.88]	1.14 [0.69-1.88]

[1]Adjusted on region of residence, number of consultations and number of hospitalizations
[2]Except for heart transplantation
[3]Except for mucoviscidosis and lung transplantation

© 2023 Kolla E et al. *JAMA Network Open.*

eTable 14.2 Factors associated with hospitalization for Covid-19 in the multivariable model (sensitivity analysis of the period before the largest epidemic wave)

	Adjusted Odds Ratios aOR [IC95%][1]			
	**Kidney**	**Liver**	**Heart**	**Lung**
**Number of hospitalizations cases/Effective**	**2686/41463**	**479/14464**	**306/5327**	**205/2823**
**Age groups**				
01-45	1	1	1	1
46-65	1.39 [1.23-1.56]	1.42 [1.03-1.95]	0.91 [0.65-1.26]	0.74 [0.50-1.09]
66 and up	1.41 [1.23-1.61]	1.57 [1.11-2.21]	1.31 [0.91-1.88]	0.60 [0.35-1.01]
**Sex**				
Male	1	1	1	1
Female	0.92 [0.84-1.00]	0.88 [0.71-1.09]	1.02 [0.76-1.36]	0.97 [0.71-1.31]
**Social deprivation index**				
1 (least deprived)	1	1	1	1
2	1.13 [0.99-1.30]	0.89 [0.65-1.21]	0.95 [0.65-1.41]	0.83 [0.51-1.35]
3	1.16 [1.01-1.34]	1.00 [0.72-1.38]	0.99 [0.65-1.51]	0.89 [0.53-1.50]
4	1.37 [1.19-1.57]	1.15 [0.84-1.57]	1.00 [0.67-1.50]	1.18 [0.72-1.93]
5 (most deprived)	1.70 [1.49-1.94]	1.24 [0.91-1.68]	1.07 [0.72-1.58]	1.29 [0.80-2.09]
**Comorbidities**				
Alcohol	0.88 [0.74-1.05]	0.76 [0.61-0.94]	0.98 [0.66-1.46]	0.89 [0.53-1.50]
Smoking	0.79 [0.71-0.88]	0.76 [0.60-0.95]	0.99 [0.75-1.30]	1.13 [0.78-1.63]
Obesity	1.23 [1.11-1.35]	0.98 [0.79-1.23]	1.56 [1.18-2.06]	1.12 [0.74-1.69]
Hypertension	1.10 [0.98-1.24]	0.99 [0.79-1.24]	1.15 [0.83-1.59]	1.22 [0.89-1.69]
Cardiovascular[2]	1.21 [1.10-1.33]	1.44 [1.16-1.79]	0.92 [0.64-1.33]	1.08 [0.78-1.50]
Statin use	0.92 [0.85-1.01]	1.07 [0.85-1.33]	1.17 [0.89-1.55]	1.12 [0.79-1.60]
Diabetes	1.52 [1.39-1.66]	2.03 [1.65-2.49]	1.16 [0.89-1.53]	0.99 [0.73-1.35]
Dialysis	-	3.32 [1.76-6.28]	1.85 [0.96-3.55]	1.24 [0.34-4.54]
Respiratory[3]	1.23 [1.08-1.40]	1.17 [0.89-1.55]	0.93 [0.66-1.32]	1.30 [0.88-1.92]
Cancer	0.83 [0.70-0.97]	0.64 [0.47-0.88]	1.08 [0.71-1.64]	1.00 [0.59-1.69]
Psychiatric	1.23 [1.02-1.49]	1.05 [0.74-1.51]	0.99 [0.63-1.57]	0.79 [0.46-1.36]
Antidepressants use	1.04 [0.87-1.24]	1.27 [0.89-1.81]	0.88 [0.56-1.39]	0.72 [0.42-1.21]
Anxiolytics use	1.00 [0.86-1.16]	0.87 [0.63-1.20]	1.47 [1.05-2.06]	1.22 [0.79-1.88]
**Age of Transplantation**				
Under 2 year	1	1	1	1
2-5 years	1.15 [1.01-1.31]	1.20 [0.87-1.66]	0.83 [0.54-1.26]	1.12 [0.74-1.72]
5-10 years	1.08 [0.95-1.23]	0.93 [0.67-1.29]	0.87 [0.57-1.31]	0.96 [0.60-1.52]
10 years and up	0.93 [0.81-1.06]	1.16 [0.84-1.61]	0.81 [0.54-1.22]	0.68 [0.39-1.20]
**Immunosuppressive drugs**				
Steroids	1.57 [1.42-1.74]	1.66 [1.35-2.03]	1.38 [0.97-1.97]	0.69 [0.29-1.64]
Tacrolimus	0.86 [0.75-1.00]	0.70 [0.51-0.96]	0.52 [0.33-0.84]	0.37 [0.14-0.99]
Ciclosporin	0.97 [0.82-1.14]	0.86 [0.58-1.29]	0.51 [0.32-0.80]	1.38 [0.87-2.19]
Mycophenolic Acid	1.20 [1.06-1.35]	1.48 [1.18-1.85]	1.19 [0.85-1.68]	0.78 [0.40-1.53]
Azathioprine	0.76 [0.62-0.91]	0.92 [0.47-1.80]	1.00 [0.50-2.00]	1.20 [0.24-6.03]
Sirolimus	0.93 [0.70-1.24]	1.26 [0.68-2.32]	2.74 [1.01-7.42]	1.18 [0.76-1.84]
Everolimus	0.98 [0.83-1.16]	0.88 [0.64-1.23]	1.04 [0.77-1.40]	1.14 [0.69-1.88]

[1]Adjusted on region of residence, number of consultations and number of hospitalizations
[2]Except for heart transplantation
[3]Except for mucoviscidosis and lung transplantation

© 2023 Kolla E et al. *JAMA Network Open.*

eTable 14.3 Factors associated with hospitalization for Covid-19 in the multivariable model (sensitivity analysis of the period after the largest epidemic wave)

	Adjusted Odds Ratios aOR [IC95%][1]			
	**Kidney**	**Liver**	**Heart**	**Lung**
**Number of hospitalizations cases/Effective**	**1028/37212**	**183/13722**	**165/4805**	**135/2447**
**Age groups**				
01-45	1	1	1	1
46-65	1.26 [1.04-1.51]	1.18 [0.73-1.92]	0.81 [0.53-1.24]	0.77 [0.47-1.26]
66 and up	1.58 [1.29-1.94]	1.33 [0.79-2.23]	1.19 [0.74-1.93]	0.63 [0.32-1.24]
**Sex**				
Male	1	1	1	1
Female	1.22 [1.06-1.39]	1.04 [0.74-1.44]	1.25 [0.86-1.83]	0.89 [0.61-1.31]
**Social deprivation index**				
1 (least deprived)	1	1	1	1
2	0.85 [0.68-1.05]	0.76 [0.47-1.24]	0.83 [0.47-1.46]	0.73 [0.40-1.32]
3	0.96 [0.77-1.19]	0.88 [0.54-1.44]	1.06 [0.62-1.83]	0.61 [0.32-1.15]
4	0.91 [0.73-1.13]	0.67 [0.41-1.12]	0.82 [0.47-1.43]	0.52 [0.28-0.99]
5(most deprived)	0.88 [0.71-1.09]	0.71 [0.43-1.18]	0.70 [0.40-1.24]	0.78 [0.43-1.43]
**Comorbidities**				
Alcohol	1.12 [0.86-1.45]	0.66 [0.47-0.93]	0.91 [0.52-1.57]	1.35 [0.77-2.37]
Smoking	0.90 [0.76-1.07]	0.80 [0.56-1.16]	1.03 [0.72-1.49]	1.11 [0.71-1.76]
Obesity	1.03 [0.88-1.21]	1.13 [0.80-1.60]	1.10 [0.75-1.62]	1.38 [0.86-2.24]
Hypertension	1.07 [0.89-1.28]	1.16 [0.81-1.66]	1.50 [0.95-2.37]	0.82 [0.56-1.22]
Cardiovascular[2]	1.17 [1.01-1.35]	1.09 [0.77-1.55]	1.32 [0.76-2.28]	0.98 [0.65-1.47]
Statin use	1.06 [0.93-1.22]	1.57 [1.11-2.23]	1.29 [0.89-1.87]	1.15 [0.76-1.73]
Diabetes	1.21 [1.05-1.40]	1.26 [0.91-1.75]	1.17 [0.81-1.71]	1.22 [0.83-1.78]
Dialysis	-	1.52 [0.34-6.81]	3.05 [1.37-6.77]	2.46 [0.65-9.34]
Respiratory[3]	1.12 [0.91-1.39]	0.94 [0.58-1.51]	1.52 [1.00-2.31]	1.22 [0.75-1.99]
Cancer	1.13 [0.90-1.42]	0.98 [0.63-1.53]	1.28 [0.75-2.19]	0.80 [0.41-1.56]
Psychiatric	1.45 [1.10-1.90]	1.53 [0.92-2.56]	0.78 [0.42-1.47]	1.19 [0.64-2.22]
Antidepressants use	1.04 [0.80-1.35]	2.27 [1.42-3.63]	1.12 [0.64-1.97]	1.59 [0.95-2.65]
Anxiolytics use	1.24 [1.00-1.53]	0.81 [0.48-1.35]	0.86 [0.52-1.42]	1.74 [1.08-2.80]
**Age of Transplantation**				
Under 2 year	1	1	1	1
2-5 years	1.05 [0.85-1.29]	0.64 [0.39-1.07]	1.05 [0.62-1.79]	0.82 [0.49-1.39]
5-10 years	0.96 [0.78-1.18]	0.91 [0.57-1.45]	0.60 [0.34-1.06]	0.67 [0.38-1.18]
10 years and up	0.93 [0.76-1.14]	0.63 [0.39-1.03]	0.86 [0.50-1.48]	0.99 [0.53-1.86]
**Immunosuppressive drugs**				
Steroids	1.49 [1.28-1.74]	1.43 [1.04-1.98]	1.22 [0.77-1.92]	1.99 [0.96-4.10]
Tacrolimus	1.01 [0.79-1.30]	0.79 [0.47-1.32]	1.47 [0.74-2.91]	0.83 [0.28-2.44]
Ciclosporin	1.07 [0.82-1.39]	0.92 [0.48-1.75]	1.01 [0.52-1.97]	0.74 [0.24-2.30]
Mycophenolic Acid	1.46 [1.20-1.77]	1.58 [1.11-2.25]	1.79 [1.10-2.91]	1.17 [0.66-2.08]
Azathioprine	1.22 [0.93-1.61]	1.57 [0.66-3.74]	1.90 [0.78-4.62]	0.75 [0.33-1.71]
Sirolimus	0.50 [0.28-0.88]	1.88 [0.78-4.51]	3.8 [0.99-14.61]	2.87 [0.56-14.69]
Everolimus	0.86 [0.66-1.13]	0.83 [0.50-1.38]	1.41 [0.97-2.05]	1.06 [0.60-1.88]

[1]Adjusted on region of residence, number of consultations and number of hospitalizations
[2]Except for heart transplantation
[3]Except for mucoviscidosis and lung transplantation

© 2023 Kolla E et al. *JAMA Network Open.*

eTable 15. Comparison of risk factors associated with hospitalization for Covid-19 during the period before the start of vaccination (15/02/2020-31/12/2020) and the period after the start of vaccination (01/04/2021*-31/07/2022) in the multivariable model (sensitivity analysis)

*corresponds to the period when more than 50% of patients had received at least one dose of covid-19 vaccine (in order to limit the number of patients hospitalized before being vaccinated)

	Adjusted Odds Ratios aOR [IC95%][1]							
	Kidney		Liver		Heart		Lung	
	Before the start of vaccination	After the start of vaccination	Before the start of vaccination	After the start of vaccination	Before the start of vaccination	After the start of vaccination	Before the start of vaccination	After the start of vaccination
**Number of hospitalizations cases/Effective**	**1301/41463**	**3458/39642**	**250/14464**	**583/14122**	**152/5327**	**481/5121**	**95/2823**	**376/2688**
**Age groups**								
01-45	1	1	1	1	1	1	1	1
46-65	1.75 [1.46-2.08]	1.17 [1.06-1.30]	1.30 [0.82-2.07]	1.04 [0.79-1.36]	1.10 [0.68-1.78]	0.93 [0.72-1.21]	1.09 [0.60-1.97]	0.87 [0.64-1.18]
66 and up	1.88 [1.54-2.28]	1.26 [1.12-1.41]	1.61 [0.99-2.63]	1.24 [0.93-1.67]	1.72 [1.02-2.90]	1.01 [0.75-1.36]	0.93 [0.43-1.99]	0.76 [0.50-1.15]
**Sex**								
Male	1	1	1	1	1	1	1	1
Female	0.80 [0.71-0.91]	1.17 [1.08-1.26]	0.62 [0.45-0.85]	1.11 [0.91-1.34]	1.13 [0.76-1.68]	1.05 [0.83-1.32]	0.95 [0.61-1.49]	0.96 [0.76-1.22]
**Social deprivation index**								
1 (least deprived)	1	1	1	1	1	1	1	1
2	1.13 [0.94-1.37]	1.01 [0.89-1.14]	0.87 [0.56-1.34]	1.12 [0.85-1.47]	0.74 [0.43-1.28]	1.29 [0.93-1.77]	0.79 [0.38-1.65]	0.98 [0.67-1.43]
3	1.20 [0.99-1.47]	1.06 [0.93-1.20]	1.04 [0.67-1.64]	1.05 [0.79-1.41]	0.77 [0.42-1.41]	1.17 [0.83-1.64]	1.35 [0.67-2.71]	0.89 [0.59-1.34]
4	1.40 [1.15-1.70]	1.11 [0.98-1.26]	1.31 [0.86-1.99]	0.93 [0.69-1.25]	1.11 [0.66-1.89]	0.94 [0.67-1.32]	1.35 [0.66-2.77]	0.79 [0.52-1.18]
5 (most deprived)	1.68 [1.41-2.01]	1.18 [1.04-1.33]	1.29 [0.85-1.95]	0.98 [0.73-1.32]	1.11 [0.66-1.84]	0.98 [0.70-1.37]	1.10 [0.53-2.28]	1.28 [0.87-1.88]
**Comorbidities**								
Alcohol	0.88 [0.69-1.12]	0.94 [0.81-1.10]	0.79 [0.59-1.05]	0.74 [0.61-0.90]	0.66 [0.35-1.25]	0.99 [0.71-1.37]	0.94 [0.46-1.93]	0.91 [0.62-1.35]
Smoking	0.83 [0.71-0.97]	0.88 [0.80-0.97]	0.74 [0.54-1.01]	0.81 [0.66-1.00]	1.03 [0.71-1.51]	0.95 [0.76-1.18]	0.84 [0.49-1.42]	1.16 [0.87-1.55]
Obesity	1.23 [1.07-1.40]	1.12 [1.03-1.22]	1.07 [0.80-1.44]	0.92 [0.75-1.13]	1.41 [0.96-2.07]	1.14 [0.90-1.45]	0.95 [0.53-1.72]	1.33 [0.98-1.82]
Hypertension	1.09 [0.91-1.29]	1.11 [1.00-1.23]	1.15 [0.83-1.58]	1.32 [1.07-1.62]	1.06 [0.68-1.67]	1.11 [0.86-1.43]	1.12 [0.70-1.78]	0.93 [0.72-1.18]
Cardiovascular[2]	1.20 [1.06-1.37]	1.19 [1.10-1.30]	1.36 [1.02-1.83]	1.38 [1.13-1.68]	1.13 [0.65-1.97]	1.17 [0.85-1.60]	1.11 [0.70-1.77]	1.04 [0.81-1.34]
Statin use	0.87 [0.77-0.98]	1.03 [0.95-1.11]	1.17 [0.87-1.57]	1.19 [0.97-1.46]	1.20 [0.81-1.78]	1.23 [0.98-1.54]	1.77 [1.09-2.88]	0.96 [0.74-1.26]
Diabetes	1.55 [1.37-1.76]	1.41 [1.30-1.53]	2.01 [1.51-2.66]	1.54 [1.28-1.85]	1.38 [0.95-1.99]	1.28 [1.02-1.60]	1.32 [0.85-2.06]	1.17 [0.92-1.48]
Dialysis	-	-	2.99 [1.35-6.61]	2.70 [1.36-5.35]	1.78 [0.74-4.26]	2.37 [1.4-4.00]	1.77 [0.43-7.29]	1.48 [0.55-3.95]
Respiratory[3]	1.06 [0.88-1.29]	1.33 [1.18-1.49]	1.20 [0.82-1.74]	1.08 [0.83-1.40]	0.90 [0.55-1.48]	1.16 [0.88-1.52]	1.39 [0.79-2.46]	1.24 [0.92-1.68]
Cancer	0.97 [0.78-1.20]	0.91 [0.79-1.06]	0.73 [0.48-1.10]	0.79 [0.60-1.04]	1.19 [0.68-2.08]	0.95 [0.66-1.36]	0.84 [0.40-1.77]	0.84 [0.55-1.29]
Psychiatric	1.24 [0.95-1.61]	1.11 [0.93-1.32]	1.27 [0.79-2.03]	1.04 [0.75-1.44]	1.06 [0.56-2.00]	0.86 [0.60-1.25]	1.14 [0.57-2.29]	0.90 [0.60-1.35]
Antidepressants use	1.01 [0.79-1.28]	1.13 [0.97-1.30]	1.36 [0.86-2.15]	1.27 [0.92-1.76]	0.83 [0.43-1.60]	0.91 [0.63-1.30]	0.69 [0.32-1.49]	1.16 [0.82-1.66]

© 2023 Kolla E et al. *JAMA Network Open.*

	Adjusted Odds Ratios aOR [IC95%][1]							
	Kidney		Liver		Heart		Lung	
	Before the start of vaccination	After the start of vaccination	Before the start of vaccination	After the start of vaccination	Before the start of vaccination	After the start of vaccination	Before the start of vaccination	After the start of vaccination
**Number of hospitalizations cases/Effective**	**1301/41463**	**3458/39642**	**250/14464**	**583/14122**	**152/5327**	**481/5121**	**95/2823**	**376/2688**
Anxiolytics use	1.07 [0.87-1.31]	1.05 [0.93-1.19]	1.21 [0.81-1.80]	0.96 [0.72-1.28]	1.46 [0.91-2.35]	1.14 [0.85-1.52]	1.12 [0.59-2.14]	1.40 [1.02-1.93]
**Age of Transplantation**								
Under 2 year	1	1	1	1	1	1	1	1
2-5 years	1.18 [0.98-1.42]	1.04 [0.93-1.17]	1.39 [0.88-2.20]	1.13 [0.84-1.51]	1.07 [0.58-1.96]	1.13 [0.82-1.56]	1.57 [0.84-2.93]	0.83 [0.60-1.14]
5-10 years	1.08 [0.90-1.30]	0.96 [0.86-1.08]	1.06 [0.66-1.68]	1.20 [0.90-1.60]	1.08 [0.60-1.95]	0.81 [0.58-1.13]	1.21 [0.61-2.38]	0.63 [0.44-0.90]
10 years and up	0.92 [0.76-1.11]	0.84 [0.74-0.94]	1.28 [0.81-2.04]	0.97 [0.72-1.31]	0.91 [0.50-1.66]	0.83 [0.59-1.15]	0.66 [0.28-1.56]	0.85 [0.57-1.27]
**Immunosuppressive drugs**								
Steroids	1.55 [1.34-1.79]	1.60 [1.46-1.75]	1.58 [1.19-2.09]	1.60 [1.33-1.92]	1.18 [0.73-1.92]	1.50 [1.12-2.00]	1.01 [0.51-1.99]	1.94 [1.24-3.03]
Tacrolimus	0.80 [0.66-0.97]	1.03 [0.89-1.18]	0.77 [0.50-1.18]	0.85 [0.63-1.16]	0.64 [0.34-1.23]	1.00 [0.66-1.53]	0.68 [0.20-2.29]	1.03 [0.51-2.07]
Ciclosporin	1.02 [0.82-1.27]	0.98 [0.85-1.15]	1.17 [0.70-1.97]	0.79 [0.53-1.17]	0.51 [0.27-0.97]	0.73 [0.48-1.11]	0.17 [0.03-0.87]	0.85 [0.41-1.76]
Mycophenolic Acid	1.07 [0.91-1.26]	1.54 [1.38-1.73]	1.18 [0.88-1.58]	1.85 [1.50-2.27]	1.18 [0.74-1.89]	1.28 [0.96-1.69]	1.25 [0.66-2.36]	1.38 [0.96-2.00]
Azathioprine	0.68 [0.51-0.89]	1.02 [0.87-1.21]	1.09 [0.46-2.58]	1.03 [0.57-1.85]	1.46 [0.62-3.39]	1.43 [0.83-2.44]	1.47 [0.64-3.37]	0.81 [0.48-1.36]
Sirolimus	1.09 [0.76-1.58]	0.69 [0.52-0.93]	1.30 [0.58-2.90]	1.09 [0.57-2.07]	2.83 [0.72-11.11]	2.31 [0.86-6.16]	0.70 [0.07-7.31]	2.21 [0.73-6.72]
Everolimus	1.10 [0.88-1.36]	0.83 [0.71-0.97]	1.02 [0.66-1.56]	0.76 [0.55-1.03]	1.09 [0.73-1.64]	1.27 [1.01-1.60]	1.34 [0.73-2.46]	1.12 [0.78-1.60]

[1]Adjusted on region of residence, number of consultations and number of hospitalizations

[2]Except for heart transplantation

[3]Except for mucoviscidosis and lung transplantation

© 2023 Kolla E et al. *JAMA Network Open.*

eTable 16. Comparison of risk factors associated with hospitalization for Covid-19 according to the time after transplant in the multivariable model (sensitivity analysis)

	Adjusted Odds Ratios aOR [IC95%][1]							
	Kidney		Liver		Heart		Lung	
	< 2 years since transplantation	≥ 2 years since transplantation	< 2 years since transplantation	≥ 2 years since transplantation	< 2 years since transplantation	≥ 2 years since transplantation	< 2 years since transplantation	≥ 2 years since transplantation
**Number of hospitalizations cases/Effective**	**929/6014**	**4350/35449**	**150/2200**	**775/12264**	**95/627**	**592/4700**	**123/557**	**388/2266**
**Age groups**								
01-45	1	1	1	1	1	1	1	1
46-65	1.38 [1.14-1.68]	1.27 [1.15-1.4]	1.33 [0.8-2.23]	1.18 [0.91-1.52]	0.96 [0.54-1.68]	0.96 [0.75-1.24]	0.68 [0.36-1.31]	0.92 [0.68-1.25]
66 and up	1.42 [1.14-1.78]	1.37 [1.23-1.53]	1.32 [0.71-2.48]	1.40 [1.07-1.83]	1.06 [0.44-2.58]	1.14 [0.87-1.50]	0.36 [0.12-1.03]	0.85 [0.57-1.25]
**Sex**								
Male	1	1	1	1	1	1	1	1
Female	0.98 [0.84-1.15]	1.06 [0.99-1.14]	0.94 [0.63-1.4]	0.95 [0.80-1.12]	0.86 [0.48-1.52]	1.12 [0.90-1.39]	0.73 [0.45-1.17]	1.02 [0.80-1.30]
**Social deprivation index**								
1 (least deprived)	1	1	1	1	1	1	1	1
2	1.03 [0.80-1.33]	1.07 [0.96-1.20]	1.79 [1.02-3.12]	0.88 [0.69-1.13]	2.08 [0.91-4.75]	1.06 [0.79-1.42]	1.30 [0.63-2.68]	0.87 [0.59-1.29]
3	1.21 [0.94-1.57]	1.08 [0.97-1.22]	1.23 [0.64-2.33]	1.06 [0.82-1.37]	3.64 [1.49-8.85]	0.95 [0.70-1.30]	0.81 [0.36-1.81]	0.98 [0.66-1.47]
4	1.32 [1.02-1.71]	1.18 [1.05-1.32]	1.12 [0.59-2.13]	1.00 [0.77-1.28]	1.42 [0.56-3.64]	0.92 [0.68-1.25]	0.88 [0.40-1.96]	0.95 [0.63-1.42]
5 (most deprived)	1.52 [1.19-1.94]	1.32 [1.18-1.47]	1.09 [0.56-2.11]	1.04 [0.81-1.34]	1.29 [0.52-3.16]	1.00 [0.74-1.35]	1.14 [0.52-2.48]	1.30 [0.89-1.90]
**Comorbidities**								
Alcohol	0.84 [0.62-1.13]	0.94 [0.81-1.08]	0.64 [0.42-0.99]	0.76 [0.64-0.90]	0.96 [0.45-2.07]	0.99 [0.73-1.35]	1.18 [0.58-2.40]	0.85 [0.56-1.28]
Smoking	0.75 [0.62-0.91]	0.89 [0.81-0.97]	0.88 [0.59-1.31]	0.77 [0.64-0.93]	1.03 [0.60-1.78]	0.95 [0.77-1.17]	0.86 [0.49-1.48]	1.26 [0.95-1.67]
Obesity	1.25 [1.05-1.48]	1.19 [1.10-1.29]	1.06 [0.70-1.61]	0.97 [0.81-1.16]	1.81 [0.96-3.39]	1.22 [0.98-1.51]	2.15 [1.16-3.97]	1.04 [0.76-1.44]
Hypertension	0.98 [0.80-1.19]	1.18 [1.07-1.30]	0.97 [0.64-1.45]	1.28 [1.07-1.55]	1.17 [0.62-2.21]	1.11 [0.87-1.41]	1.02 [0.63-1.64]	0.94 [0.74-1.21]
Cardiovascular[2]	1.25 [1.07-1.47]	1.21 [1.12-1.31]	1.29 [0.87-1.90]	1.36 [1.14-1.63]	2.22 [0.56-8.83]	1.18 [0.89-1.56]	1.54 [0.93-2.53]	0.98 [0.76-1.26]
Statin use	0.99 [0.84-1.16]	0.96 [0.90-1.03]	1.08 [0.65-1.79]	1.17 [0.98-1.39]	1.49 [0.79-2.82]	1.20 [0.98-1.47]	0.87 [0.47-1.60]	1.09 [0.84-1.40]
Diabetes	1.50 [1.28-1.76]	1.46 [1.36-1.57]	2.40 [1.64-3.52]	1.62 [1.37-1.90]	0.85 [0.46-1.59]	1.36 [1.11-1.67]	1.02 [0.63-1.64]	1.18 [0.92-1.50]
Dialysis	-	-	3.32 [0.74-14.98]	2.77 [1.56-4.93]	3.22 [0.83-12.42]	2.01 [1.21-3.33]	1.31 [0.06-26.57]	1.31 [0.52-3.28]
Respiratory[3]	1.38 [1.10-1.73]	1.26 [1.13-1.41]	0.77 [0.47-1.25]	1.27 [1.01-1.59]	0.67 [0.36-1.25]	1.21 [0.93-1.57]	2.12 [1.09-4.12]	1.21 [0.90-1.62]
Cancer	0.74 [0.51-1.08]	0.92 [0.81-1.04]	0.67 [0.43-1.02]	0.77 [0.59-1.00]	0.22 [0.03-1.89]	1.06 [0.78-1.44]	1.13 [0.46-2.75]	0.74 [0.49-1.12]
Psychiatric	1.44 [1.03-2.02]	1.15 [0.98-1.35]	0.98 [0.55-1.75]	1.10 [0.82-1.48]	1.38 [0.72-2.64]	0.81 [0.55-1.19]	0.52 [0.26-1.05]	1.26 [0.83-1.91]
Antidepressants use	1.01 [0.73-1.40]	1.09 [0.95-1.25]	1.41 [0.68-2.93]	1.21 [0.91-1.61]	0.52 [0.19-1.39]	0.91 [0.65-1.28]	1.19 [0.54-2.60]	1.04 [0.72-1.49]
Anxiolytics use	1.07 [0.83-1.38]	1.06 [0.94-1.19]	0.88 [0.48-1.6]	0.99 [0.77-1.27]	1.39 [0.66-2.93]	1.21 [0.93-1.58]	1.77 [0.96-3.28]	1.18 [0.84-1.65]
**Immunosuppressive drugs**								

© 2023 Kolla E et al. *JAMA Network Open.*

	Adjusted Odds Ratios aOR [IC95%][1]							
	Kidney		Liver		Heart		Lung	
	< 2 years since transplantation	≥ 2 years since transplantation	< 2 years since transplantation	≥ 2 years since transplantation	< 2 years since transplantation	≥ 2 years since transplantation	< 2 years since transplantation	≥ 2 years since transplantation
**Number of hospitalizations cases/Effective**	**929/6014**	**4350/35449**	**150/2200**	**775/12264**	**95/627**	**592/4700**	**123/557**	**388/2266**
Steroids	1.61 [1.29-2.02]	1.64 [1.52-1.77]	1.68 [1.15-2.47]	1.63 [1.38-1.92]	5.51 [0.67-45.17]	1.41 [1.11-1.80]	0.46 [0.10-2.00]	1.79 [1.21-2.64]
Tacrolimus	0.87 [0.65-1.16]	1.01 [0.90-1.15]	0.37 [0.18-0.73]	0.88 [0.68-1.14]	1.21 [0.44-3.33]	0.81 [0.56-1.18]	0.18 [0.04-0.78]	1.21 [0.56-2.61]
Ciclosporin	1.06 [0.78-1.45]	1.00 [0.87-1.14]	0.70 [0.29-1.67]	0.87 [0.62-1.20]	0.43 [0.16-1.16]	0.67 [0.47-0.97]	1.96 [0.81-4.72]	1.01 [0.45-2.30]
Mycophenolic Acid	1.04 [0.80-1.34]	1.47 [1.33-1.63]	1.05 [0.66-1.66]	1.77 [1.48-2.10]	2.40 [0.78-7.38]	1.27 [0.99-1.63]	0.89 [0.22-3.61]	1.38 [0.97-1.95]
Azathioprine	0.90 [0.62-1.30]	0.91 [0.78-1.06]	0.80 [0.25-2.55]	0.99 [0.58-1.69]	0.91 [0.09-9.21]	1.31 [0.81-2.12]	1.61 [0.03-80.52]	0.81 [0.49-1.34]
Sirolimus	0.76 [0.22-2.62]	0.79 [0.63-1.00]	1.15 [0.12-11.58]	1.23 [0.74-2.03]	8.78 [0.38-205.31]	2.36 [1.01-5.53]	0.60 [0.22-1.59]	1.56 [0.50-4.83]
Everolimus	0.99 [0.76-1.29]	0.91 [0.79-1.06]	0.86 [0.51-1.44]	0.83 [0.63-1.09]	1.23 [0.66-2.30]	1.26 [1.02-1.57]	1.05 [0.30-3.66]	1.35 [0.96-1.90]

[1]Adjusted on region of residence, number of consultations and number of hospitalizations

[2]Except for heart transplantation

[3]Except for mucoviscidosis and lung transplantation

© 2023 Kolla E et al. *JAMA Network Open.*

eTable 17. Association between immunosuppressive treatment regimens and risk of hospitalization for Covid-19 in multivariate analysis among SOTRs (Exploratory analysis)

	Adjusted Odds Ratios aOR [IC95%]				
	**Whole Cohort**	**Kidney transplant**	**Liver transplant**	**Heart transplant**	**Lung transplant**
**Treatment regimen[1]**					
Tacrolimus-Mycophenolic Acid-Steroids	1	1	1	1	1
Tacrolimus-Mycophenolic Acid	0.54 [0.50-0.59]	0.61 [0.55-0.67]	0.65 [0.53-0.80]	0.58 [0.37-0.91]	0.48 [0.29-0.79]
Ciclosporin-Mycophenolic Acid-Steroids	0.94 [0.86-1.03]	1.05 [0.95-1.17]	1.72 [1.16-2.56]	0.70 [0.54-0.90]	0.70 [0.44-1.11]
Ciclosporin-Mycophenolic Acid	0.66 [0.57-0.75]	0.73 [0.63-0.84]	0.48 [0.30-0.75]	0.74 [0.48-1.15]	0.60 [0.20-1.79]
Tacrolimus only	0.29 [0.24-0.35]	0.40 [0.29-0.54]	0.34 [0.26-0.45]	0.61 [0.18-2.05]	0.27 [0.06-1.18]
Tacrolimus-Steroids	0.70 [0.62-0.79]	0.75 [0.65-0.87]	0.67 [0.49-0.93]	0.61 [0.35-1.05]	0.54 [0.30-0.96]
Tacrolimus-Sirolimus/Everolimus-Steroids	0.83 [0.71-0.96]	0.74 [0.61-0.89]	0.74 [0.43-1.27]	1.22 [0.83-1.78]	0.91 [0.64-1.30]
Tacrolimus-Azathioprine-Steroids	0.67 [0.57-0.80]	0.66 [0.55-0.80]	0.54 [0.24-1.20]	1.08 [0.55-2.13]	0.61 [0.38-0.99]
Ciclosporin-Steroids	0.56 [0.46-0.68]	0.60 [0.49-0.74]	0.36 [0.15-0.84]	0.67 [0.40-1.12]	0.56 [0.19-1.67]
Mycophenolic Acid-Steroids	1.10 [0.94-1.29]	1.10 [0.94-1.30]	1.13 [0.61-2.06]	3.10 [1.20-7.96]	0.58 [0.06-5.03]
Sirolimus/Everolimus-Mycophenolic Acid	0.45 [0.35-0.58]	0.42 [0.28-0.61]	0.67 [0.47-0.96]	0.79 [0.37-1.67]	2.13 [0.20-21.83]
Sirolimus/Everolimus-Mycophenolic Acid-Steroids	0.94 [0.78-1.12]	0.87 [0.70-1.07]	1.13 [0.64-1.97]	1.62 [1.03-2.55]	0.76 [0.21-2.79]
Others	0.59 [0.54-0.65]	0.62 [0.56-0.69]	0.59 [0.47-0.75]	0.80 [0.63-1.01]	0.68 [0.49-0.95]
**Treatment regimen classes[2]**					
CNI + Antimétabolites + Steroids	1	1	1	1	1
CNI + mTORi + Steroids	0.83 [0.72-0.95]	0.75 [0.64-0.90]	0.80 [0.49-1.33]	1.07 [0.79-1.45]	1.00 [0.72-1.41]
Antimetabolite + mTORi + Steroids	0.93 [0.78-1.11]	0.84 [0.69-1.03]	1.00 [0.57-1.74]	1.98 [1.29-3.04]	0.70 [0.19-2.51]
CNI + Antimetabolite + mTORi	0.47 [0.33-0.67]	0.38 [0.17-0.82]	0.52 [0.28-0.96]	0.93 [0.54-1.58]	0.56 [0.19-1.65]
CNI + Antimétabolites	0.58 [0.54-0.62]	0.63 [0.58-0.68]	0.62 [0.51-0.75]	0.77 [0.56-1.05]	0.52 [0.33-0.81]
CNI + mTORi	0.47 [0.37-0.61]	0.55 [0.40-0.77]	0.47 [0.29-0.74]	0.62 [0.31-1.21]	1.04 [0.37-2.95]
CNI + Steroids	0.68 [0.61-0.76]	0.72 [0.63-0.81]	0.60 [0.44-0.82]	0.74 [0.51-1.09]	0.60 [0.36-1.02]
Antimetabolite + Steroids	1.04 [0.90-1.20]	1.02 [0.88-1.18]	1.15 [0.65-2.02]	2.72 [1.12-6.61]	0.85 [0.17-4.15]
Antimetabolite + mTORi	0.47 [0.37-0.60]	0.46 [0.32-0.65]	0.64 [0.45-0.91]	0.93 [0.44-1.96]	2.26 [0.22-23.20]
mTORi + Steroids	0.70 [0.54-0.92]	0.77 [0.57-1.02]	0.66 [0.33-1.32]	0.78 [0.17-3.49]	0.27 [0.03-2.10]
CNI	0.31 [0.26-0.36]	0.38 [0.30-0.50]	0.36 [0.28-0.46]	0.50 [0.17-1.40]	0.24 [0.05-1.01]
Antimetabolite	0.72 [0.56-0.92]	0.69 [0.49-0.97]	1.00 [0.70-1.42]	-	1.89 [0.16-21.92]
mTORi	0.26 [0.16-0.43]	0.25 [0.07-0.80]	0.33 [0.19-0.57]	0.47 [0.06-3.76]	2.56 [0.20-32.46]
Others	0.94 [0.80-1.10]	0.78 [0.60-1.01]	0.63 [0.34-1.17]	1.23 [0.95-1.58]	1.07 [0.69-1.66]
**Immunosuppression intensity (including steroids)[3]**					
Triple regimen	1	1	1	1	1
Double regimen	0.65 [0.61-0.68]	0.71 [0.66-0.76]	0.66 [0.56-0.78]		
Single regimen	0.38 [0.33-0.43]	0.47 [0.38-0.57]	0.45 [0.37-0.56]	0.72 [0.58-0.90]	0.58 [0.42-0.79]

CNI: Calcineurin Inhibitors / mTORi: mammalian target of rapamycin inhibitors
[1]Model 1: Adjusted for age, sex, social deprivation index, each of the comorbidities, number of consultations, number of hospitalizations, age of transplantation, and region of residence.
[2]Model 2: Adjusted for age, sex, social deprivation index, each of the comorbidities, number of consultations, number of hospitalizations, age of transplantation, and region of residence.
[3]Model 3: Adjusted for age, sex, social deprivation index, each of the comorbidities, number of consultations, number of hospitalizations, age of transplantation, and region of residence.
-Estimation was not possible

© 2023 Kolla E et al. *JAMA Network Open.*

eFigure 1. Kinetics of hospitalizations for Covid-19 among SOTRs

(Red: whole transplant population; Green: Kidney; Blue: Liver; Dark yellow: Heart; Pink: Lung)

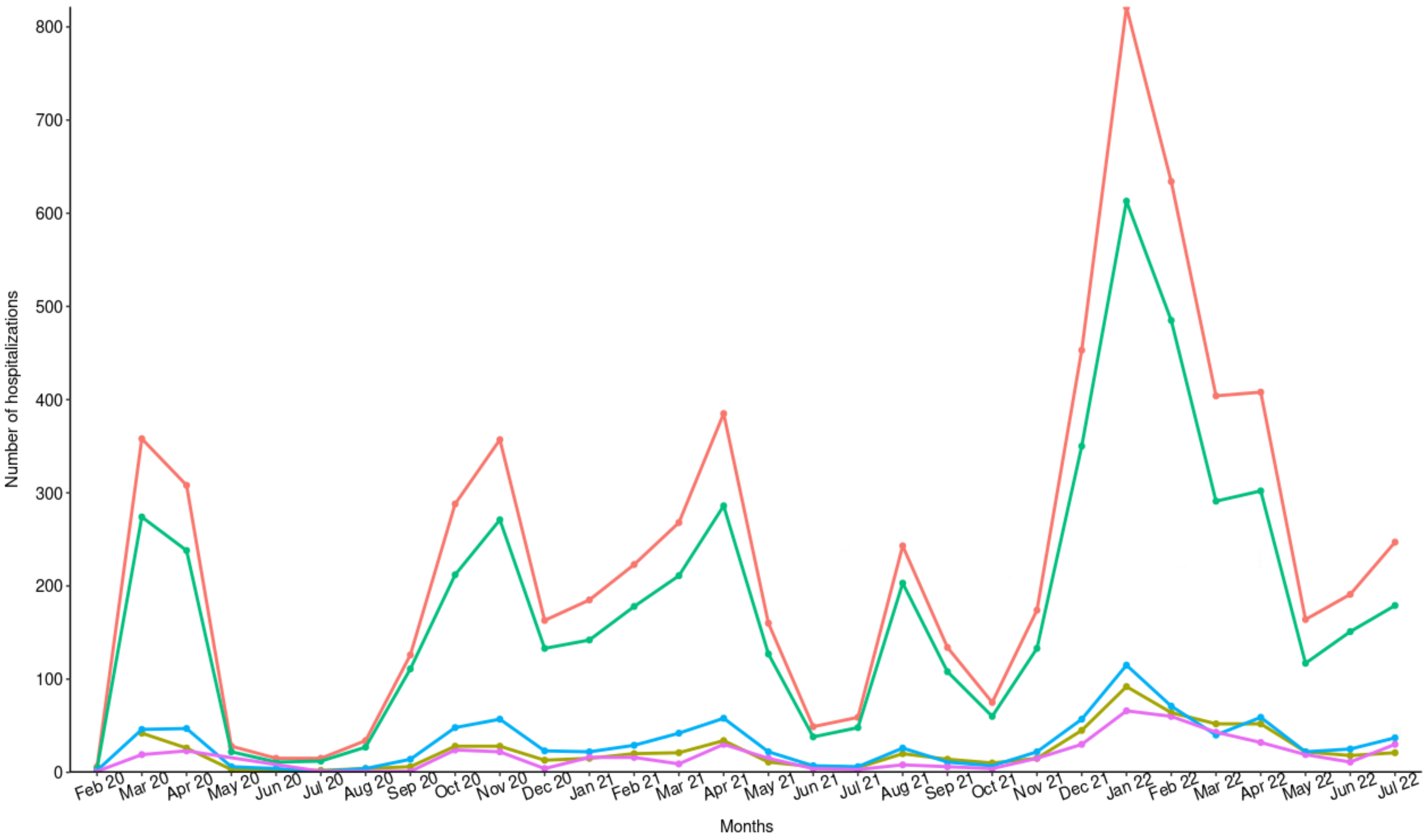

© 2023 Kolla E et al. *JAMA Network Open.*

eFigure 2. Immunosuppressive drugs use according to the time since transplant in KTRs.

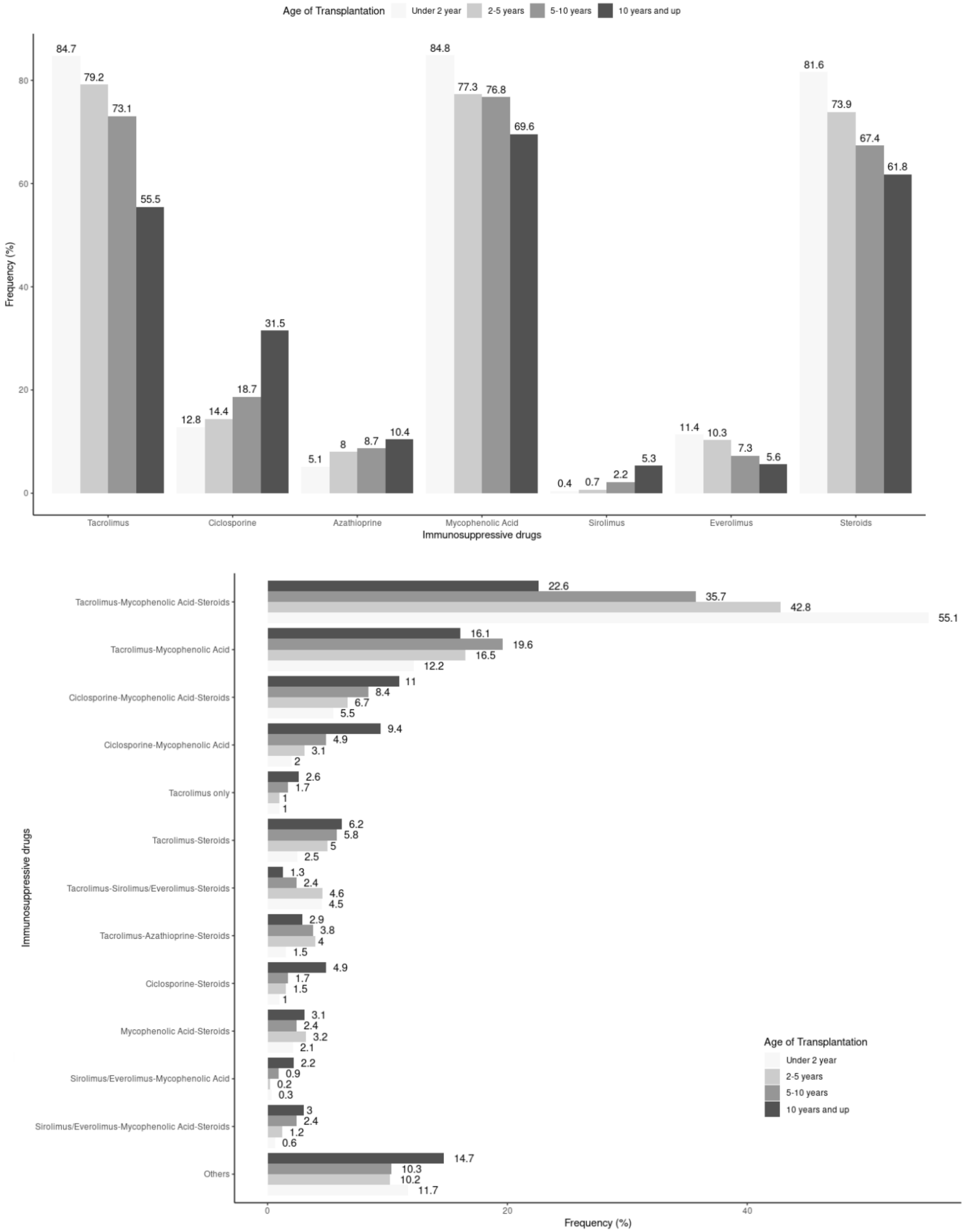

© 2023 Kolla E et al. *JAMA Network Open.*

eFigure 3. Immunosuppressive drugs use according to the time since transplant in LTRs.

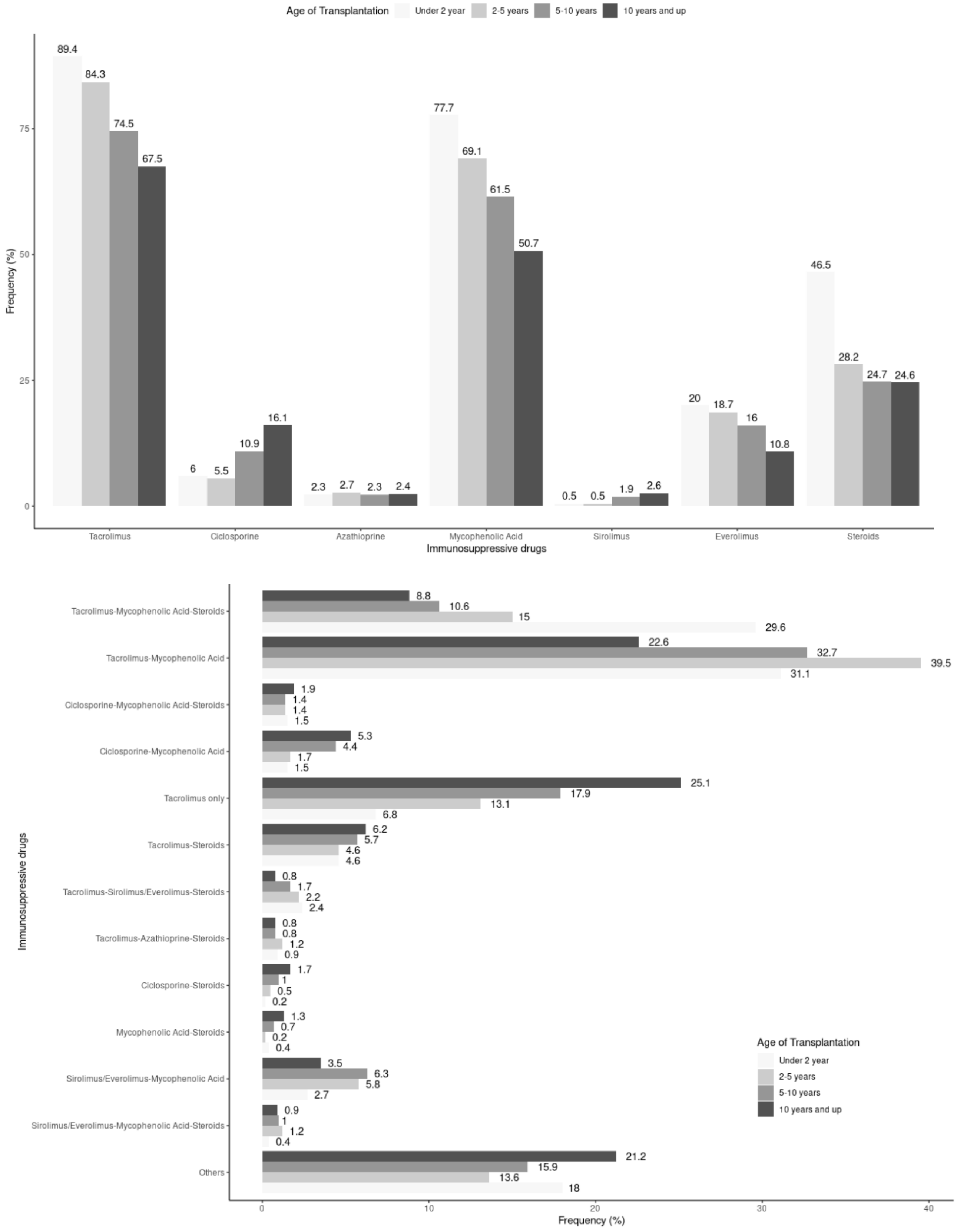

© 2023 Kolla E et al. *JAMA Network Open.*

eFigure 4. Immunosuppressive drugs use according to the time since transplant in HTRs.

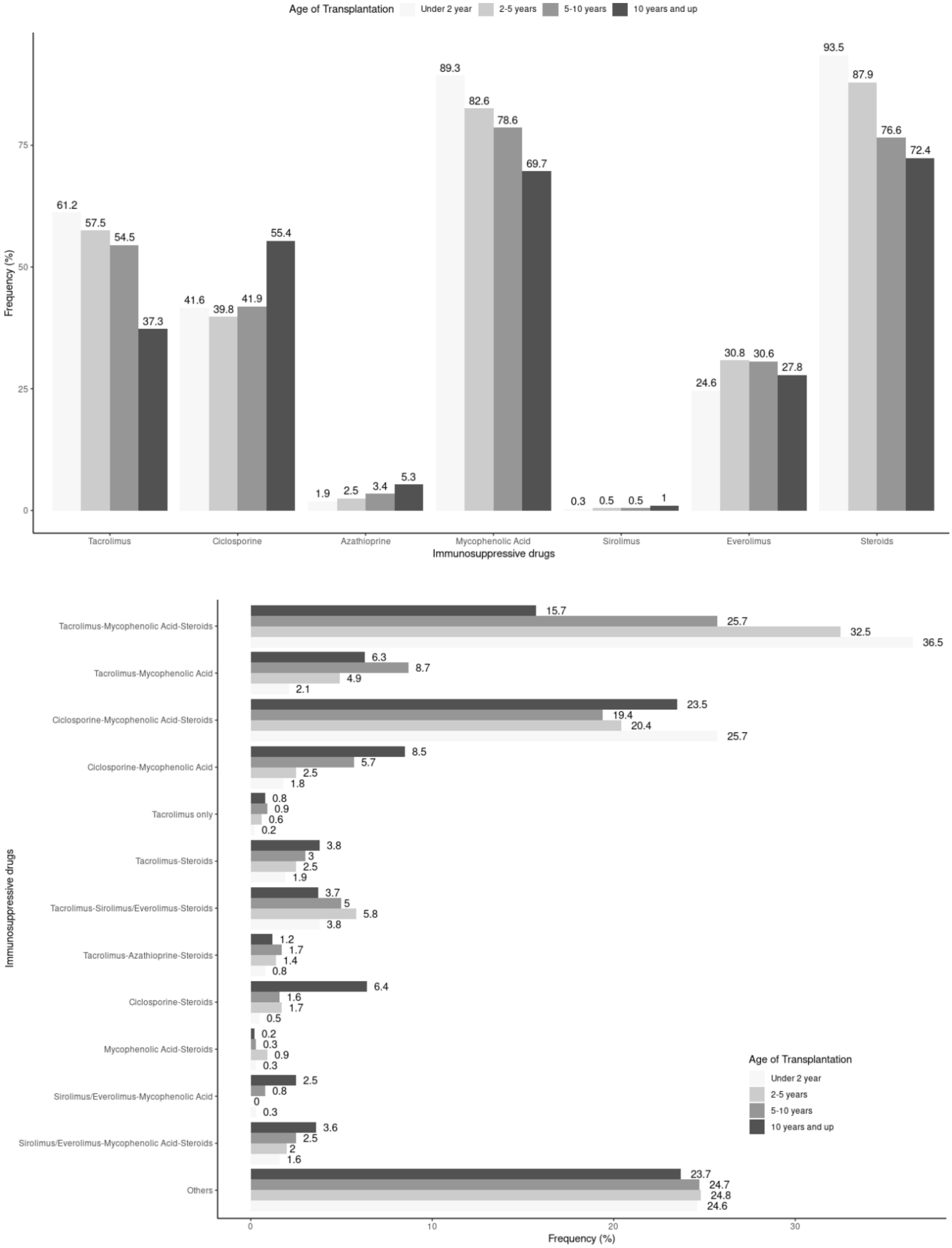

© 2023 Kolla E et al. *JAMA Network Open.*

eFigure 5. Immunosuppressive drugs use according to the time since transplant in lung transplant recipients.

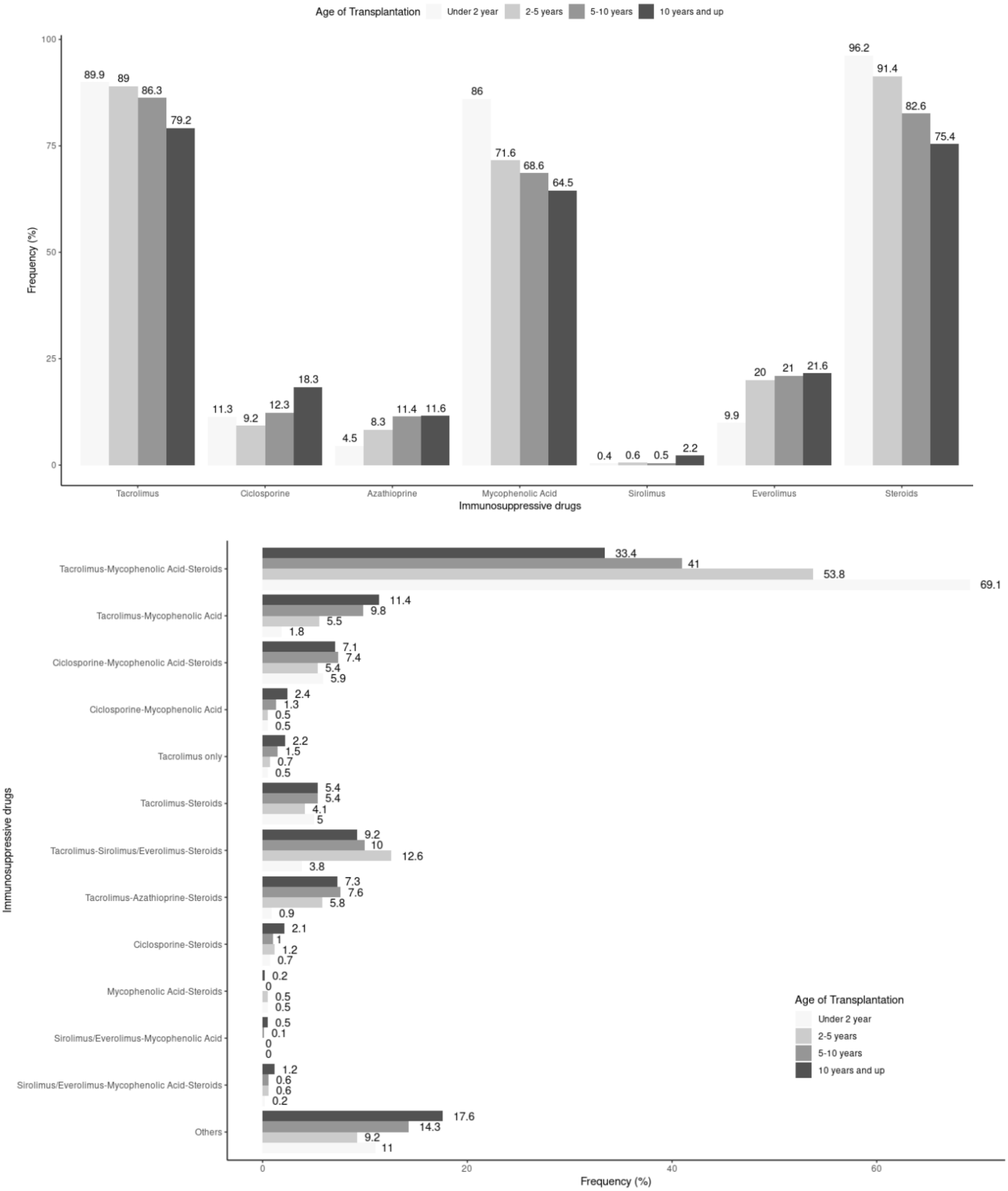

© 2023 Kolla E et al. *JAMA Network Open.*

eFigure 6. Description of treatment regimens in SOTRs (Whole population N=60456)

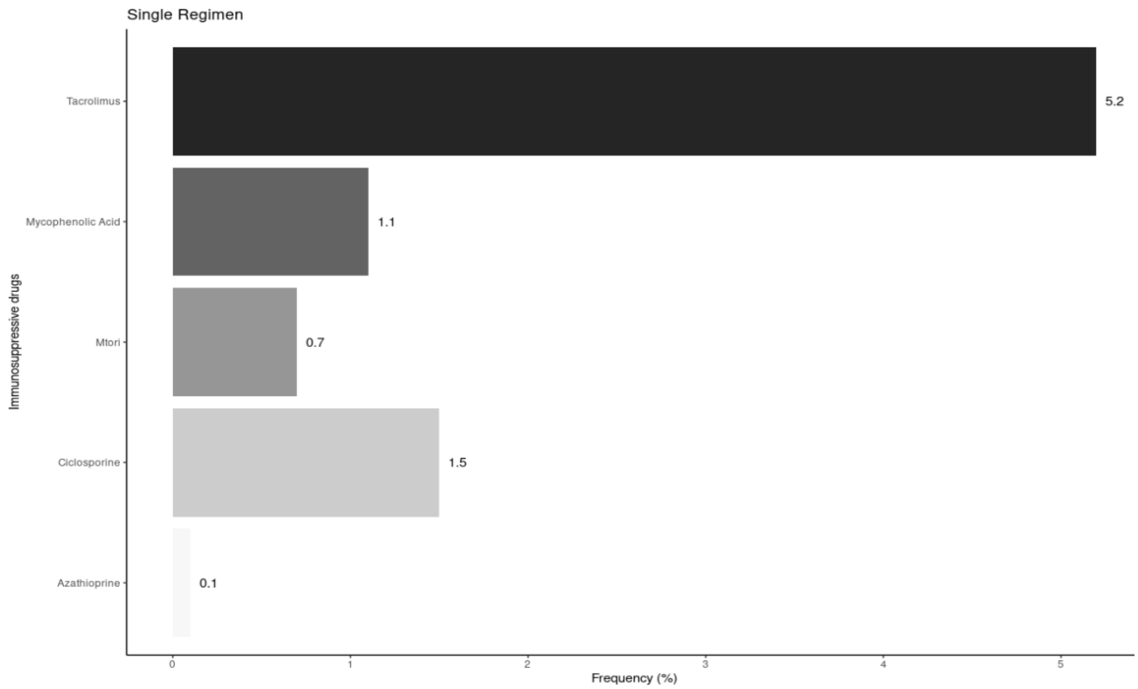

© 2023 Kolla E et al. *JAMA Network Open.*

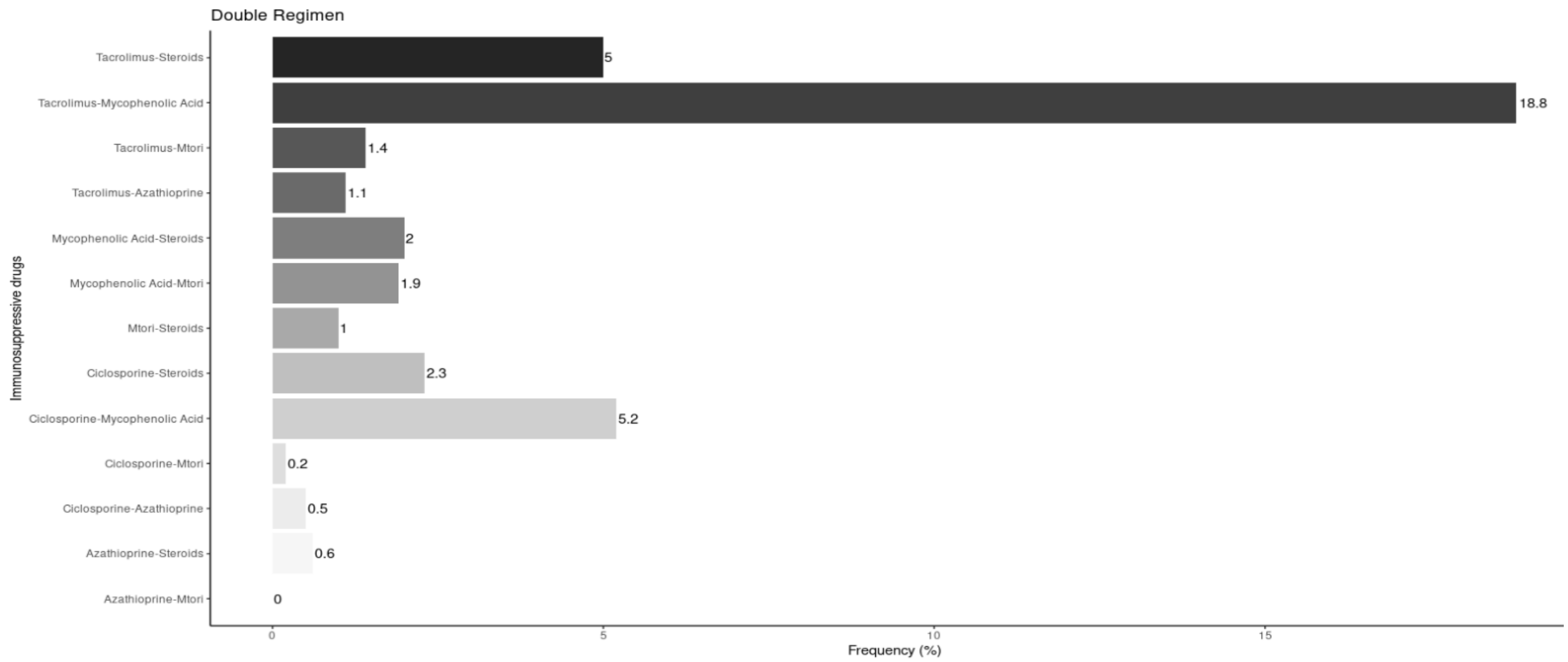

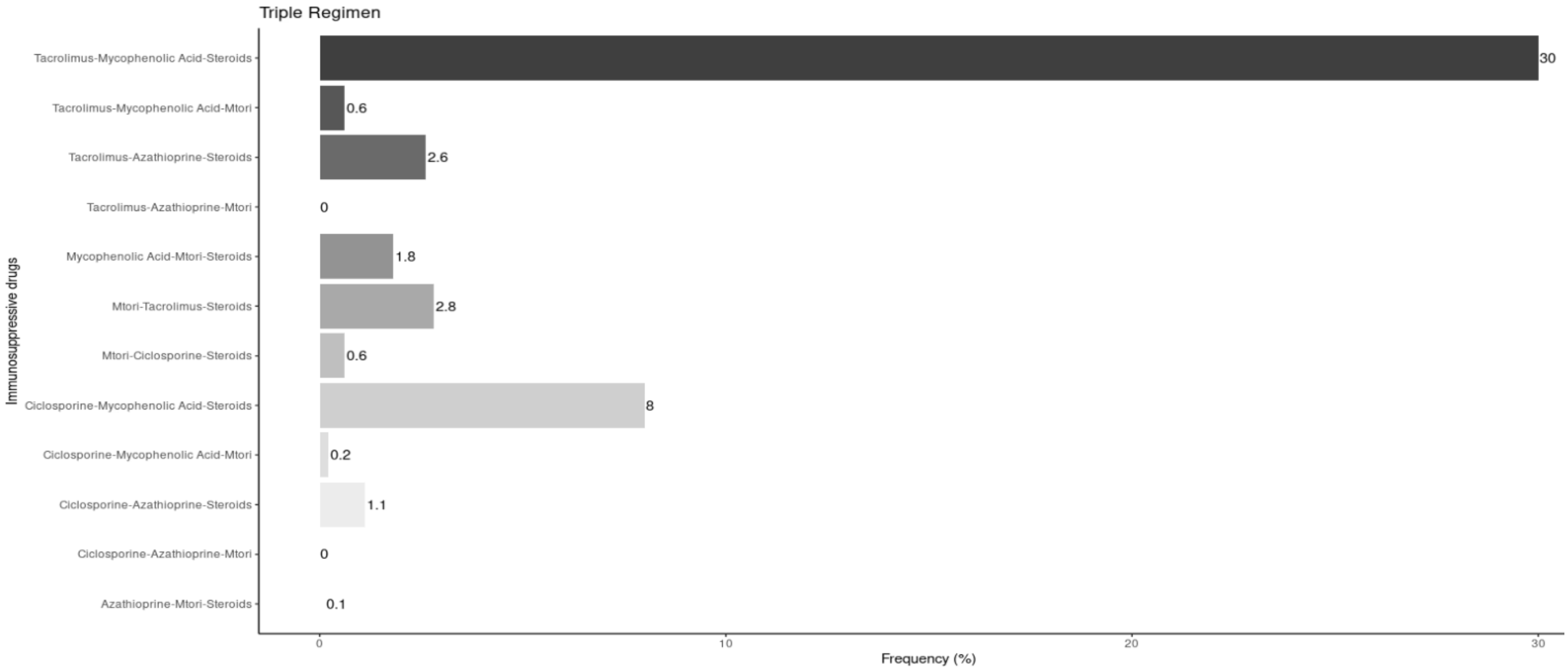

© 2023 Kolla E et al. *JAMA Network Open.*
