# Supplementary material for: COVID-19 Hospitalization in Solid Organ Transplant Recipients on Immunosuppressive Therapy
Source: JAMA Netw Open. 2023 Nov 7;6(11):e2342006. doi: 10.1001/jamanetworkopen.2023.42006 (PMC10630896; doi:10.1001/jamanetworkopen.2023.42006)
Supplement: Supplement 2. — Data Sharing Statement [file jamanetwopen-e2342006-s002.pdf]

Data Sharing Statement

Kolla. COVID-19 Hospitalization in Solid Organ Transplant Recipients on Immunosuppressive Therapy. *JAMA Netw Open*. Published November 08, 2023.
doi:10.1001/jamanetworkopen.2023.42006

Data
Data available: No

Additional Information
Explanation for why data not available: According to data protection and French regulations, the authors cannot publicly release the data from the French National Health Data System (SNDS). However, any person or organisation (public or private; for-profit or non-profit), can access anonymised SNDS data to do a study, research, or an evaluation of public interest, upon authorisation from the French Data Protection Office (See https://www.snds.gouv.fr/SNDS/Processus-d-acces-aux-donnees and https://documentation-snds. health-data-hub.fr/introduction/03-acces-snds.html).
